# Synthesis of eunicellane-type bicycles embedding a 1,3-cyclohexadiene moiety

**DOI:** 10.3762/bjoc.14.222

**Published:** 2018-09-20

**Authors:** Alex Frichert, Peter G Jones, Thomas Lindel

**Affiliations:** 1 Institute of Organic Chemistry, TU Braunschweig, Hagenring 30, 38106 Braunschweig, Germany, Fax: (+49) 531-391-7744; 2 Institute of Inorganic and Analytical Chemistry, TU Braunschweig, Hagenring 30, 38106 Braunschweig, Germany

**Keywords:** conformational analysis, low valent titanium, marine natural products, pinacol coupling, ten-membered rings

## Abstract

The first synthesis of diterpenoid eunicellane skeletons incorporating a 1,3-cyclohexadiene moiety is presented. Key step is a low-valent titanium-induced pinacol cyclization that proved to be perfectly diastereoselective. Determination of the relative configuration of the diol was aided by the conversion to the diastereomer by oxidation and reduction. Conformational analysis of some of the resulting diols obtained under McMurry conditions was complicated by the presence of several conformers of similar energy. The pinacol coupling appears to start at the ketone, as indicated by the selective reduction of non-cyclizing cyclohexane systems that were synthesized from limonene oxide. The title compounds and their synthetic precursors are prone to aromatization on contact with air oxygen. Attempted synthesis of cyclohexene-containing eunicellane bicycles by elimination of water from tertiary alkynyl carbinols afforded novel allene systems. Our study may be of help towards the total synthesis of solenopodin or klysimplexin derivatives.

## Introduction

**Figure 1:**
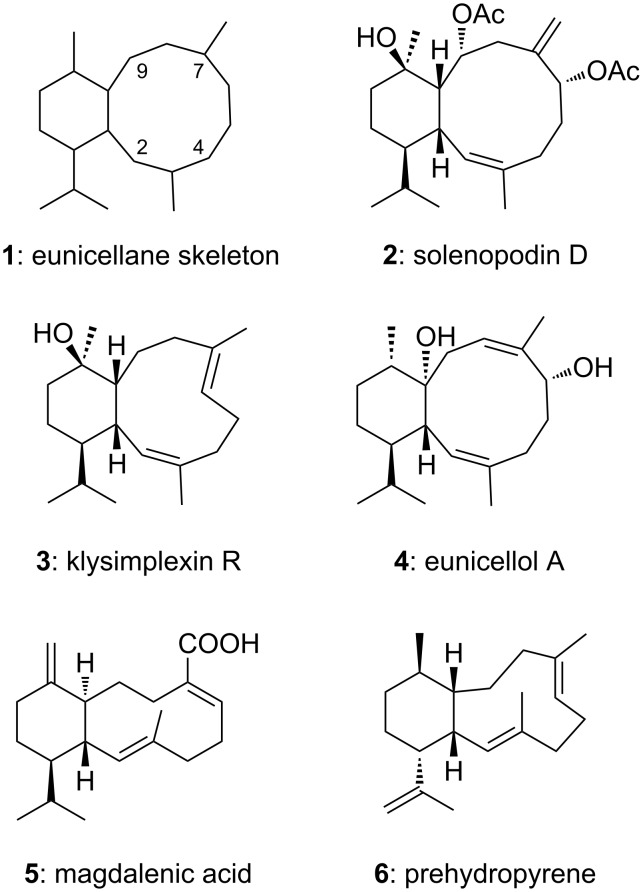
Bicyclic eunicellane-type diterpenes.

Eunicellane-type diterpenoids share an [8.4.0] bicyclic skeleton (**1**, Figure 1). In many cases, an additional oxygen bridge is present between positions 4 and 7, or 2 and 9, but there are also interesting eunicellanes without oxygen bridge, the majority of which has been isolated from marine corals. These comprise the solenopodins A–D (**2**, solenopodin D) from *Solenopodium stechei* [1], an unnamed eunicellane [2] and the klysimplexins Q and R (**3**, klysimplexin R) [3] from *Eunicella labiata*, cladieunicelline F from *Cladiella* sp. [4] and eunicellol A (**4**) from the soft coral *Gersemia fruticose* [5]. Magdalenic acid (**5**) was isolated from the plant *Vellozia magdalenae* [6]. Recently, prehydropyrene (**6**) was discovered as biosynthetic intermediate towards the diterpene hydropyrene from the Gram-positive bacterium *Streptomyces clavuligerus* [7]. The six- and ten-membered rings of eunicellane diterpenoids can be either *cis* or *trans* fused, and the ten-membered ring may contain (*Z*)- or (*E*)-double bonds. None of them has been synthesized.

**Figure 2:**
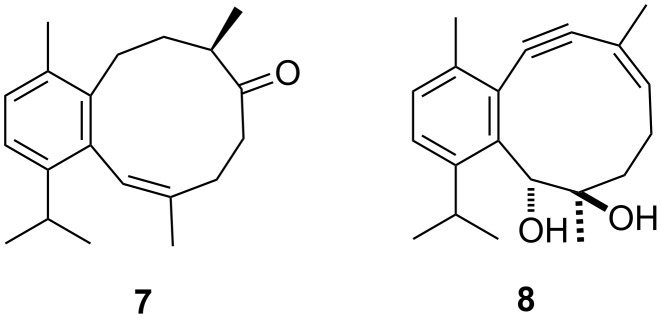
Synthetic eunicellane-type compounds with benzene partial structure.

A series of eunicellane-type bicycles containing a cyclohexene partial structure served as intermediates of the total syntheses of the sarcodictyins, the eleuthesides, and of eleutherobin [8–9]. The benzene-containing eunicellane derivative **7** (Figure 2) was obtained synthetically starting from the cembranoid sarcophytoxide from the soft coral *Sarcophytum glaucum* [10]*.*

We have shown that eunicellane **8** containing a benzene partial structure can be accessed efficiently via pinacol cyclization of a ketoaldehyde precursor [11]. However, compound **8** proved to be very resistant towards any attempt of partial hydrogenation of the benzene moiety. Systems embedding a cyclohexadiene ring should be more versatile, e.g., by allowing regio- and stereoselective hydrogenation and oxygenation towards partial structures present in compounds **2**–**6**. In addition, we expected that the higher hydrogen count and the non-planarity of the envisaged 1,3-cyclohexadiene ring would allow the determination of the diastereoselectivity of the coupling step more precisely than in the case of aromatic **8** that exists as mixture of two conformers in CDCl_3_.

Thus, it was to be explored how open-ring cyclohexadiene precursors would be synthesized and behave under McMurry conditions, and how stable the resulting [8.4.0]bicycles would be. Normally, McMurry conditions lead to the formation of alkenes, but medium-sized ring 1,2-diols have also been obtained (TiCl_4_/Zn [12–17] or TiCl_3_/Zn-Cu [18–20]), often as mixture of diastereomers. The use of samarium diiodide to achieve the pinacol coupling was not advised, since we had observed that intially formed ketyl radicals would add to the alkyne moiety [11], even if there are examples, where this was not the case [21–22]. Access to partially unsaturated eunicellane systems could also be of interest for studies on biosynthesis and chemical interconversion [7,10].

## Results and Discussion

**Scheme 1:**
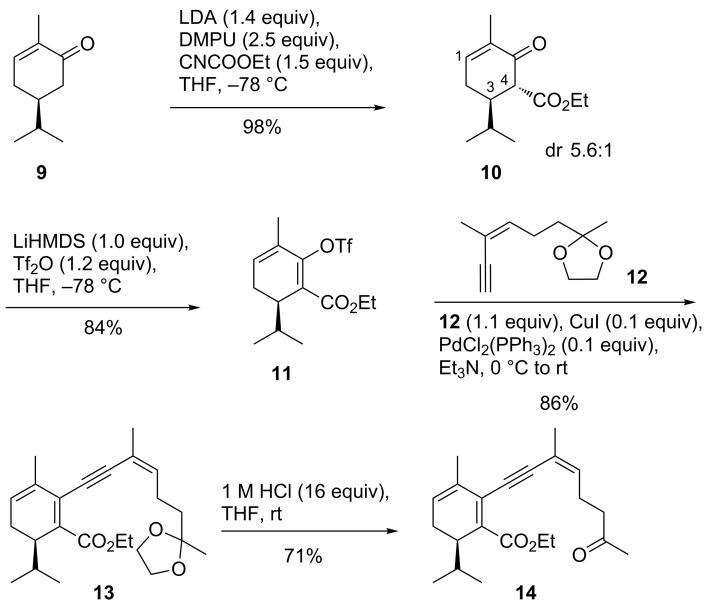
Access to ketoester **14** that did not cyclize to the ethyl vinyl ether under McMurry conditions.

Dihydrocarvone **9** was converted to the enolate and quenched with ethyl cyanoformate in the presence of DMPU affording an inconsequential 5.6:1 mixture of diastereomers favoring **10** (^3^*J*_3H-4H_ 12.2 Hz vs 4.8 Hz, Scheme 1). The cyclohexadiene system of **11** was formed after deprotonation of **10** (LiHMDS) and quenching with triflic anhydride. All compounds carrying the 1,3-cyclohexadiene motif were prone to aromatization and had to be protected from contact with air and higher temperatures. Removal of solvents was performed below 21 °C and all compounds were stored under argon at −18 °C. Sonogashira coupling (PdCl_2_(PPh_3_)_2_) of dienol triflate **11** with alkyne **12** [11] provided C_20_ ester **13** in a good yield of 86%. However, after hydrolysis of the 1,3-dioxolane moiety to ketoester **14** it proved to be impossible to induce any McMurry cyclization involving the two carbonyl groups of the system. Only starting material was isolated. We had hoped that the ester might participate in the cyclization, since there is precedence of accessing medium-sized ring ethyl vinyl ethers when employing TiCl_3_/LiAlH_4_/NEt_3_ in DME instead of TiCl_4_/Zn/pyridine [23].

**Scheme 2:**
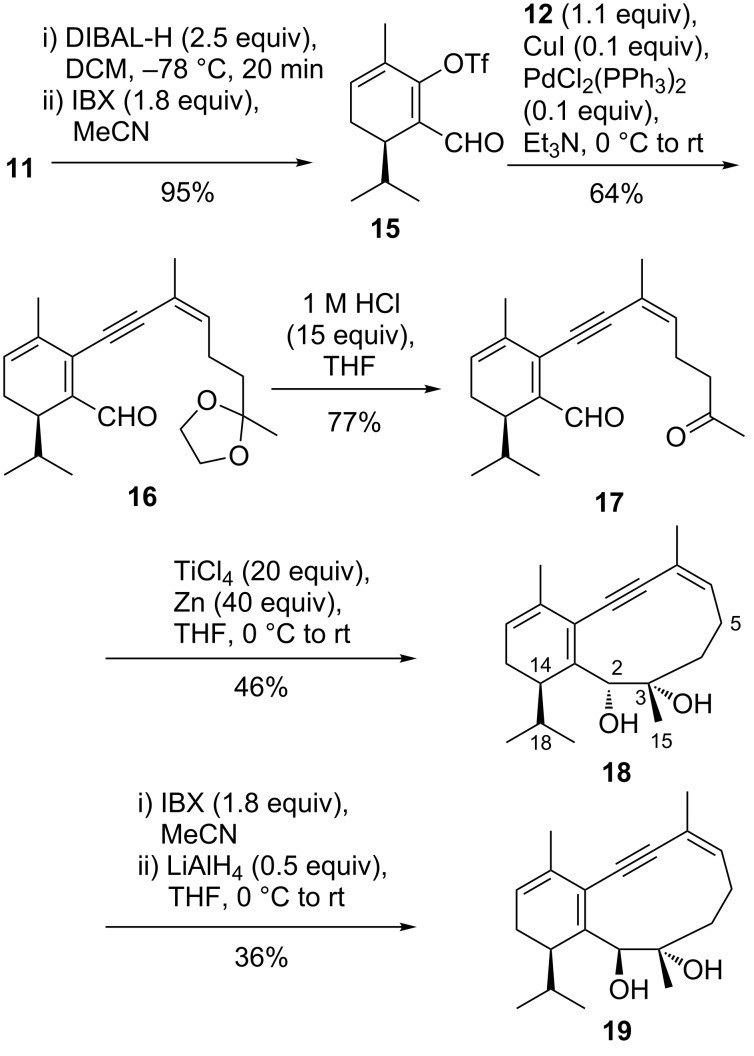
Synthesis of the 1,3-cyclohexadiene-containing eunicellane-type [8.4.0]bicycle **18** by McMurry coupling to the diol, followed by two-step epimerization at C2.

Direct reduction of ester **14** to the aldehyde proved to be surprisingly difficult with either DIBAL-H or LiAlH_4_, when the alkynyl side chain was in place. Thus, we reduced the ester function of dienol triflate **11** to the alcohol (DIBAL-H, DCM, −78 °C), followed by oxidation to aldehyde **15** (IBX, Scheme 2). Fortunately, the cyclohexadiene moiety survived the oxidation conditions, which was not the case when using PCC or MnO_2_. Subsequent Sonogashira coupling to **16** and deprotection to **17** worked satisfyingly. As in the case of a benzene partial structure (**8**), it was a diol that was formed from **17** under McMurry conditions (20 equiv TiCl_4_, 40 equiv Zn, THF, rt). No alkene was detected.

**Figure 3:**
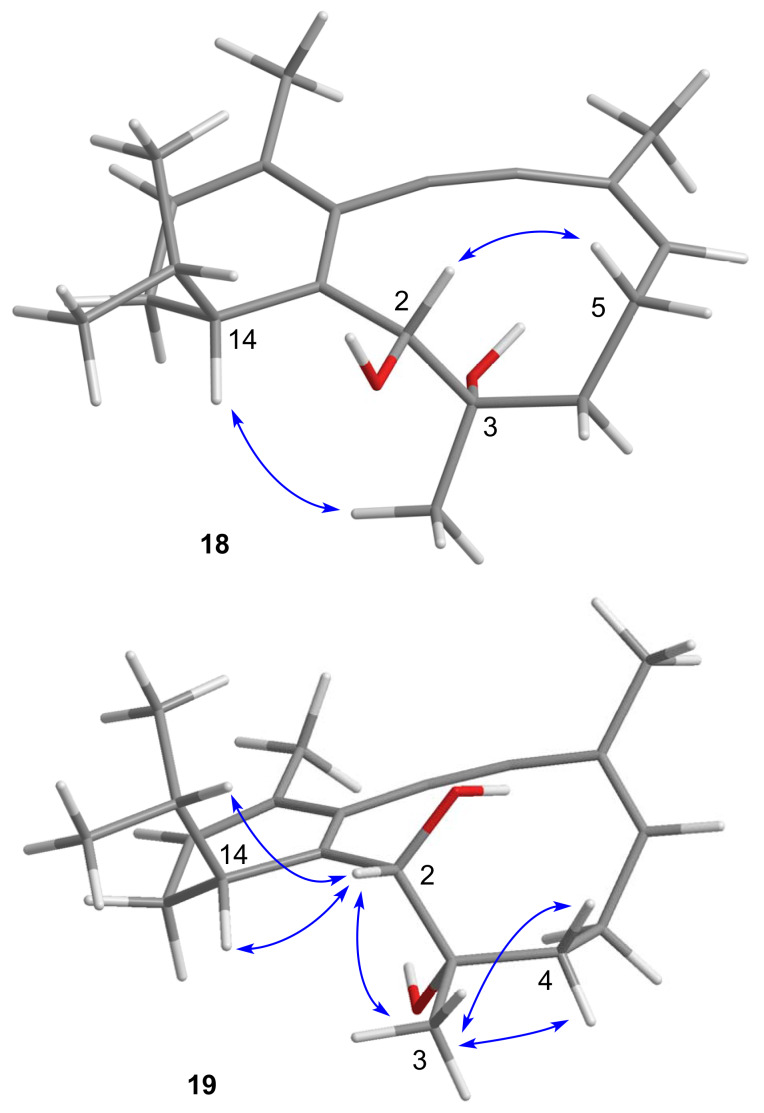
Preferred conformations of diastereomeric diols **18** and **19** including decisive NOESY correlations.

McMurry-type pinacol cyclizations have shown varying degrees of diastereoselectivity, which means that every single example has to be explored independently. The assignment of the configuration and preferred conformation of product **18** was conducted on the basis of NOESY NMR experiments. Our analysis was aided by the observation that the 2-epimer **19** was accessible by oxidation of **18** to the acyloin (IBX, MeCN) and subsequent reduction (LiAlH_4_, THF, Scheme 2). Diastereomers **18** and **19** show distinct sets of NMR signals. The largest chemical shift differences are observed for the secondary carbinol group (δ_2-H_ 5.18, δ_C2_ 73.1 for **18** vs δ_2-H_ 4.14, δ_C2_ 87.2 for **19**). For diol **18**, we observed key NOESY correlations between carbinol 2-H (δ 5.18) and one of the C5 methylene hydrogens (δ 2.37) and between 14-H (δ 2.43) and 3-CH_3_ (δ 1.20, Figure 3). For each of the four diastereomers with 14*R* configuration we found one conformation (MM2) placing 2-H and one 5-H in proximity. Moreover, those conformations are within 10 kJ/mol range of each other. However, only one of those conformations shows the required short distance between 14-H and 3-CH_3_, making the configuration (2*R*,3*S*,14*R*) probable for diastereomer **18**. In the preferred conformation of **18**, all non-sp^3^-hybridized carbon atoms of the ring system and the adjacent atoms are located almost in plane, whereas the isopropyl group and ring carbons C3 and C4 are located on opposite sides of that plane. Since the oxidation/reduction sequence has affected the configuration at C2, diastereomer **19** is assigned the configuration (2*S*,3*S*,14*R*). For diastereomer **19**, 2-H did not show a NOESY correlation to 5-Hβ, but instead a correlation to 14-H, to the isopropyl methine hydrogen, and to 3-CH_3_. In addition, 3-CH_3_ correlates with both methylene hydrogens at C4. We found only two conformers of the (2*S*,3*S*,14*R*) diastereomer that meet those constraints. One of them is shown in Figure 3.

**Scheme 3:**
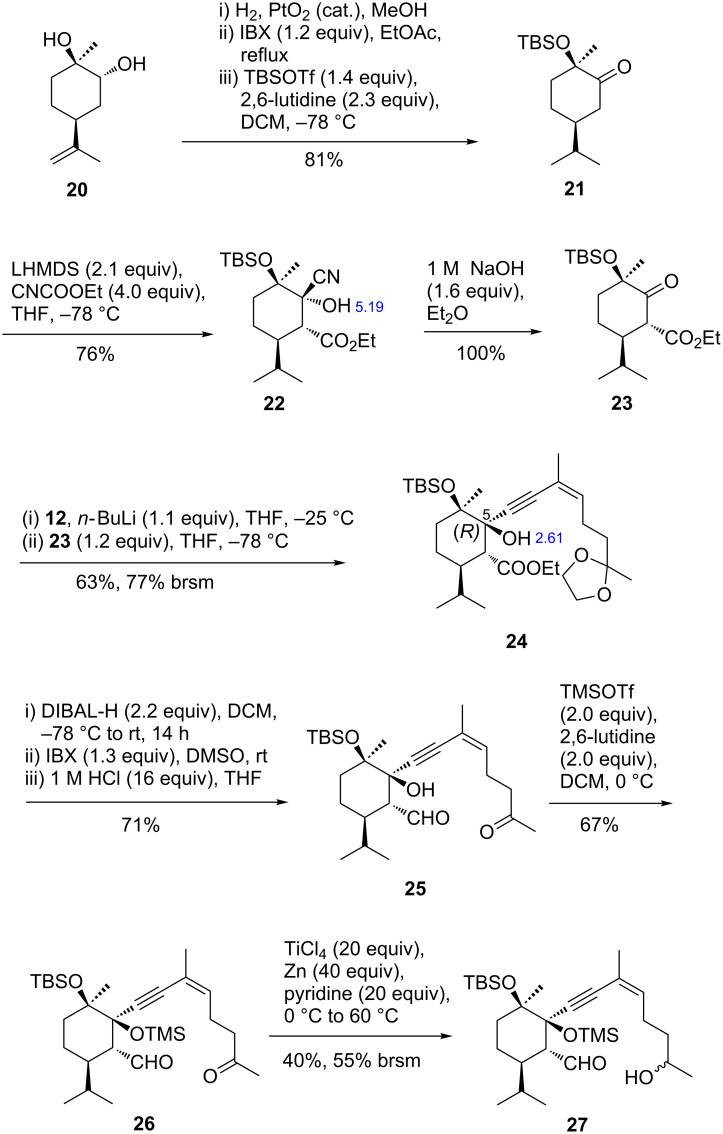
Assembly of the envisaged cyclization precursor **27**.

The transfer of the pinacol McMurry route to the formation of eunicellane-type bicycles with two sp^3^ centers as bridgeheads will not be straight-forward. This became clear on our attempts to cyclize model compound **25** that was synthesized within nine steps (Scheme 3). For the synthesis of **25**, we started from the known limonene oxide-derived diol **20** [24] that was hydrogenated, oxidized, and silylated at the tertiary alcohol moiety (81%). Reaction of deprotonated **21** with ethyl cyanoformate afforded cyanohydrin **22** by attack of liberated cyanide at the carbonyl carbon following the ethoxycarbonylation. The ^3^*J*_HH_ coupling constant proved that the isopropyl and ethoxycarbonyl groups both assume an equatorial position in a chair conformation.

**Scheme 4:**
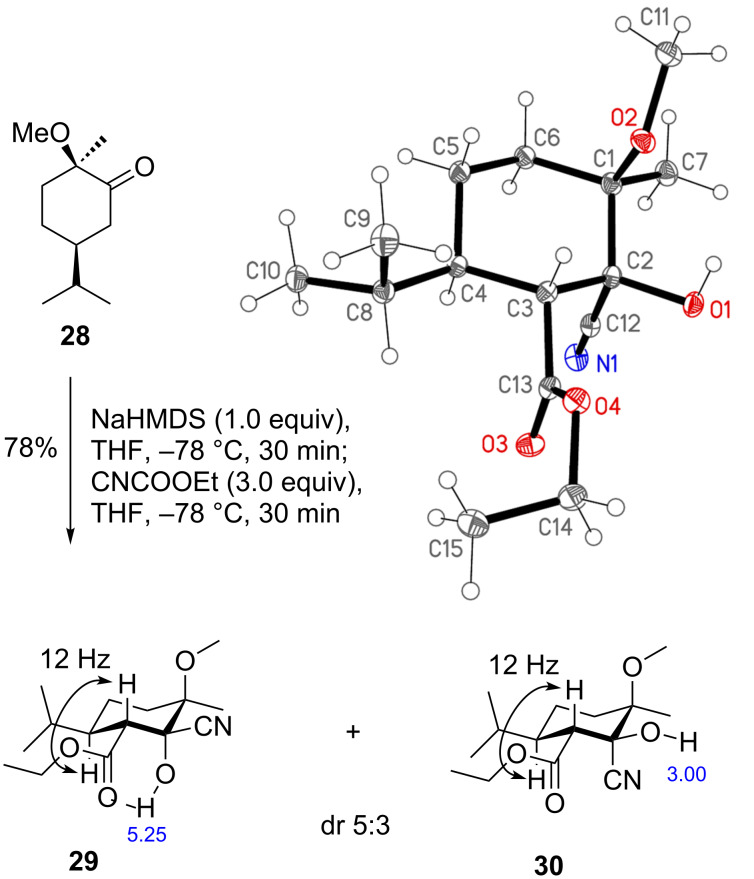
Structure analysis of diastereomeric cyanohydrins **29** and **30**.

We were not able to obtain an X-ray analysis of cyanohydrin **22**, but of one diastereomer (**30**) of an analog where the OTBS was replaced by a methoxy group (Scheme 4, obtained by ethoxycarbonylation of **28**) [25]. In agreement with the NMR data, cyanohydrin **30** adopts a chair conformation in the crystal. The ^1^H NMR spectra of diastereomers **29** and **30** differ characteristically regarding the chemical shift of the hydroxy proton which appeared as a sharp signal at 5.25 ppm (CDCl_3_) for **29** and as a broad peak at 3.00 ppm for **30**. This can be explained by the presence of an intramolecular hydrogen bond that is possible only in the case of **29**. Since the ^1^H NMR spectrum of cyanohydrin **22** exhibits a sharp hydroxy peak at 5.29 ppm, we derive the relative configuration shown in Scheme 4.

From cyanohydrin **22** HCN was eliminated by treatment with diluted NaOH (100%, Scheme 3). The resulting ketone **23** reacted with lithiated alkyne **12** affording diastereomerically pure tertiary alcohol **24** (63%) that showed a broad hydroxy signal in the ^1^H NMR spectrum at 2.61 ppm. This indicates that the alkynyl side chain had been introduced from the same side as the ethoxycarbonyl group. By conversion of the ester to an aldehyde group and deprotection we obtained the subject of study, the putative cyclization precursor **25** (71%). In an orienting reaction, treatment of **25** with TiCl_4_/Zn did not lead to pinacol cyclization and we have evidence that the aldehyde group stayed in place and the keto group had been reduced. Installation of a TMS group at the tertiary alcohol moiety of **25** (TMSOTf, 2,6-lutidine) formed **26**, which was simply reduced at the keto function on reaction with TiCl_4_/Zn/pyridine (**27**, Scheme 3) without cyclization. As before, the aldehyde had stayed in place. Interestingly, treatment of **25** with samarium diiodide afforded the primary alcohol and left the keto group unchanged. Still, no cyclization took place.

**Scheme 5:**
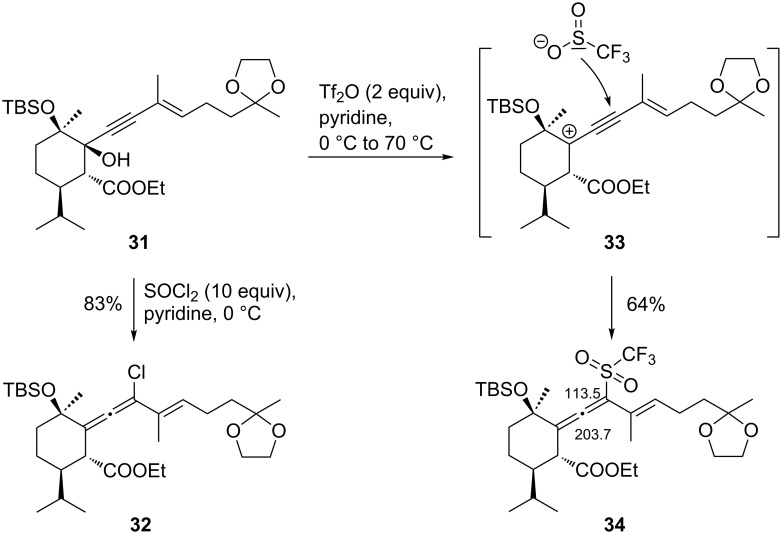
Formation of allenes **32** and **34** from sterically crowded propargylic alcohol **31**.

One could think that endocyclic elimination of water from **24** to the α,β-unsaturated ester would afford a cyclohexene system that would regain the ability of undergoing pinacol cyclization, because the bridge of the [8.4.0] system would become a double bond. However, standard protocols (MsCl/Et_3_N or *p*-TsOH) failed. We also synthesized the (*E*)-isomer of **24**, compound **31**, via Sonogashira coupling with the (*E*)-isomer [11] of **12**. Chlorinated allene **32** was formed from **31** as the only product on treatment with SOCl_2_/pyridine, presumably after chlorosulfonation, followed by chlorine transfer and loss of SO_2_ (Scheme 5). There is precedence that elimination towards the cyclohexene can be a competing process [26]. The configuration of the allene moieties could not be elucidated.

Interestingly, treatment of **31** with Tf_2_O/pyridine afforded allenyl triflone **34**, with ^13^C NMR signals of the allene center and the triflyl-substituted carbon at δ 203.7 and 113.5 ppm, respectively. The sequence probably commences with pyridine-assisted conversion of propargylic alcohol **31** to the propargyl triflate, which looses triflate, forming propargyl cation **33**. Since a triflone is formed rather than a triflate, reduction of the incoming nucleophile must have taken place, probably before incorporation. Corey and Tian reported the formation of 4-substituted *N*-triflyldihydropyridine derivatives on reaction of pyridine (4 equiv) with Tf_2_O (1.5 equiv) in the presence of aryl nucleophiles that occurred already at −30 °C within 30 min. Conversion to the corresponding pyridine derivatives was possible on treatment with KO*t*-Bu at −23 °C, presumably with formation of potassium triflinate [27]. In our case, pyridine was used as solvent and the reaction mixture was heated up to 70 °C. This could allow pyridine itself taking the double role of nucleophile and base, leading to the formation of pyridinium triflinate. It has also been reported that the triflinate anion can be generated from 2,6-lutidine/Tf_2_O [28] or Et_3_N/Tf_2_O [29]. Allenyl triflone **34** could be formed by attack of triflinate as S-nucleophile [30] at the chain carbon of propargyl cation **33**. An alternative would be the attack of triflinate as O-nucleophile at the cyclohexane carbon, followed by [2,3]-sigmatropic rearrangement [31].

## Conclusion

With the synthesis of the [8.4.0]bicycles **18** and **19** that contain a 1,3-cyclohexadiene partial structure, we have made progress towards the synthesis of a small group of bicyclic diterpenoids sharing the eunicellane skeleton. Closure of the ten-membered ring by pinacol cyclization proved to be possible, if the six-membered ring is either aromatic or a 1,3-cyclohexadiene, but failed for systems with two sp^3^ centers as bridgeheads. The ten-membered ring of benzene-containing **8** adopts two distinct major conformations in CDCl_3_, whereas diastereomeric diols **18** and **19** prefer only one, which were elucidated by NOESY spectroscopy. In upcoming studies we will address the synthesis of systems that contain a cyclohexene ring keeping the sp^2^–sp^2^ bridge of the product from the beginning, and of precursors in which one of the centers will be sp^2^- and the other sp^3^-hybridized. Examples of the latter have already been obtained in form of allenes **32** and **34** that will now have to be elaborated further.

## Supporting Information

File 1 Experimental procedures, spectroscopical data, X-ray analysis of **30**, and NMR spectra plots.
