# Supplementary material for: Synthesis of eunicellane-type bicycles embedding a 1,3-cyclohexadiene moiety
Source: Beilstein J Org Chem. 2018 Sep 20;14:2461–7. doi: 10.3762/bjoc.14.222 (PMC6176824; doi:10.3762/bjoc.14.222)
Supplement: File 1 — Experimental procedures, spectroscopical data, X-ray analysis of 30, and NMR spectra plots. [file Beilstein_J_Org_Chem-14-2461-s001.pdf]

Supporting Information

for

Synthesis of eunicellane-type bicycles embedding a 1,3-cyclohexadiene moiety

Alex Frichert,[1] Peter G. Jones,[2] and Thomas Lindel*,§,1

Address: [1]Institute of Organic Chemistry, TU Braunschweig, Hagenring 30, 38106 Braunschweig, Germany, Fax: (+49) 531-391-7744 and [2]Institute of Inorganic and Analytical Chemistry, TU Braunschweig, Hagenring 30, 38106 Braunschweig, Germany

Email: Thomas Lindel - th.lindel@tu-braunschweig.de

*Corresponding author

§www.oc.tu-bs.de/lindel

Experimental procedures, spectroscopical data, X-ray analysis of 30, and NMR spectra plots

Materials and methods

Chemicals were purchased and, if not stated otherwise, used without further purification. Solvents were distilled prior to use or purchased. For reactions under an argon atmosphere, solvents were dried and distilled under argon using a Solvent Purification System. Yields that are given for each step refer to purified compounds. Optical rotatory power was recorded with a Dr. Kernchen Propol automatic polarimeter at the given concentration [$[\alpha]_D$: deg·cm^3·g^{-1}·dm^{-1}, c: g/100 cm^3]. Solvents are given in the procedures. NMR spectra were recorded with a Bruker AVII-300 (300 MHz for ^{1}H, 75.5 MHz for ^{13}C, T = 297 K), a Bruker AVIIIHD-300N (300 MHz for ^{1}H, 75.5 MHz for ^{13}C, T = 298 K), a Bruker DRX400 (400 MHz for ^{1}H, 100.6 MHz for ^{13}C, T = 298 K), a Bruker AVIII-400 (400 MHz for ^{1}H, 100.6 MHz for ^{13}C, T = 297 K), a Bruker AVIII-HD500, (500 MHz for ^{1}H, 125.8 MHz for ^{13}C, T = 298 K) and a Bruker AVII-600 (600 MHz for ^{1}H, 150.9 MHz for ^{13}C, T = 303 K). Chemical shifts (δ) are given in ppm as referenced to tetramethylsilane (TMS). Signals were assigned with the aid of ^{1}H,^{1}H-COSY,

$^{1}H,^{13}C$-HSQC, $^{1}H,^{13}C$-HMBC, and $^{1}H,^{1}H$-NOESY experiments. Mass spectra were recorded with EI and ESI ionization methodology. For EI measurements, a Finnigan MAT 95 XL (Thermofinnigan MAT) or a GC-EIMS system consisting of an Agilent 6890 gas chromatograph (column: Phenomenex ZB5-MS, 30 m length × 0.25 mm internal diameter, 0.25 µm phase density) and a JMST100GC (GCAccuTOF, JEOL, Japan) mass spectrometer were used. For ESI measurements, a LTQ Orbitrap Velos (ThermoFisher Scientific) was employed. IR spectra were recorded with a Bruker Tensor 27 spectrometer. UV-vis spectra were recorded with a Varian Cary 100 Bio UV/Vis-spectrometer. Solvents are given in the procedures. Melting points were determined with a Büchi 530 or a Büchi M-560 device and are uncorrected. Thin layer chromatography (TLC) was performed on Merck silica 60 F_{254} aluminum sheets. Column chromatography was performed using Merck Geduran® silica 40-63 µm at elevated pressure. Eluents are given in the procedures.

Ethyl (1*R*,6*R*)-6-isopropyl-3-methyl-2-oxocyclohex-3-ene-1-carboxylate (10)

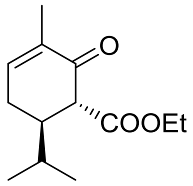

10

At −78 °C, a solution of *n*-BuLi in hexanes (1.6 M, 34.5 mL, 55.18 mmol) was added dropwise to a solution of diisopropylamine (8.3 mL, 59.12 mmol) in dry THF (100 mL). The resulting solution was stirred for 30 min at −78 °C, before a solution of dihydrocarvone **9** (6.00 g, 39.41 mmol) and DMPU (12 mL, 99.24 mmol) in dry THF (50 mL) was added dropwise. After 1 h ethyl cyanoformate (5.858 g, 59.12 mmol) was added in one portion and the mixture was stirred for 5 min at −78 °C. The reaction was quenched by addition of saturated aqueous NH_4Cl (50 mL) at –78 °C, and the reaction mixture was allowed to warm to rt. The aqueous layer was extracted with DCM (4 × 150 mL), the organic phase was dried over $MgSO_4$ and the solvent was removed under reduced pressure. The residue was purified by column chromatography (silica, petroleum ether/EtOAc, 10:1), to give a 5.6:1 mixture of diastereomers **10a,b** (8.69 g, 38.74 mmol, 98%) as colorless oil. – R_f (petroleum ether/EtOAc, 5:1) = 0.34. – $[\alpha]_D^{21}$ = –6.2 (*c* = 1.0, $CHCl_3$). – ^{1}H NMR (400 MHz, $CDCl_3$, **10a**): δ = 6.77 (qdd, *J* = 1.4, 2.7, 5.5 Hz, 1H, C_qCHCH_2), 4.24 (m, 2H, CH_2CH_3), 3.32 (d, *J* = 12.2 Hz, 1H, CHC_qOOEt), 2.45 (m, 1H, $(CH_3)_2CHCH$), 2.35 (m, 1H, CHC*H*HCH), 2.17 (m, 1H, CHCH*H*CH), 1.79 (td, *J* = 1.4, 2.7 Hz, 3H, CH_3C_q), 1.72 (m, 1H, $(CH_3)_2CH$), 1.29 (t, *J* = 7.2 Hz, 3H, CH_3CH_2), 0.96 (d, *J* = 6.9 Hz, 3H, CH_3CHCH_3), 0.88 (d, *J* = 6.9 Hz, 3H, CH_3CHCH_3). – ^{13}C NMR (100 MHz, $CDCl_3$, **10a**): δ = 195.5 (1C, C_qC_q=O), 170.5 (1C, C_qOOEt), 144.9 (1C, CH_3C_qCH), 134.7 (1C, CH_3C_q), 60.9 (1C, CH_2CH_3), 58.6 (1C, CHC_qOOEt), 43.1 (1C, $(CH_3)_2CHCH$), 29.0 (1C, $(CH_3)_2CH$), 24.6 (1C, $CHCH_2CH$), 20.4 (1C, CH_3CHCH_3), 16.4 (1C, CH_3CHCH_3), 15.7 (1C, CH_3C_q), 14.2 (1C, CH_3CH_2). – IR (ATR): $\tilde{\nu}$ = 2961 (w), 1737 (s), 1072 (w), 1671 (s), 1369 (m), 1255 (m), 1153 (m), 1026 (m), 568 (w) cm^{-1}. – ^{1}H NMR (400 MHz, $CDCl_3$, **10b**): δ = 6.84 (m, 1H, C_qCHCH_2), 4.14 (m, 2H, CH_2CH_3), 3.64 (d, *J* = 4.8 Hz, 1H, CHC_qOOEt), 2.44 (m, 2H, $CHCH_2CH$), 1.84 (m, 1H, $(CH_3)_2CHCH$), 1.81 (dd, *J* = 1.8, 3.5 Hz, 3H, CH_3C_q), 1.67 (m, 1H, $(CH_3)_2CH$), 1.27 (t, *J* = 7.1 Hz, 3H, CH_3CH_2), 1.00 (d, *J* = 6.6 Hz, 3H, CH_3CHCH_3), 0.96 (d, *J* = 6.6 Hz, 3H, CH_3CHCH_3). – ^{13}C NMR (100 MHz, $CDCl_3$, **10b**): δ = 195.0 (1C, C_qC_q=O), 168.7 (1C, C_qOOEt), 146.9 (1C, CH_3C_qCH), 133.9 (1C, CH_3C_q), 60.3 (1C, CH_2CH_3), 55.5 (1C, CHC_qOOEt), 44.9 (1C, $(CH_3)_2CHCH$), 30.2 (1C, $(CH_3)_2CH$), 28.1 (1C, $CHCH_2CH$), 20.5 (1C, CH_3CHCH_3), 20.4 (1C, CH_3CHCH_3), 15.9 (1C, CH_3C_q), 14.6 (1C, CH_3CH_2). – MS (GC-MS, EI): *m/z* (%) = 224 (4) $[M]^+$, 181 (29), 135 (17), 109 (100), 91 (17), 82 (49), 79 (17), 54 (16), 41 (26). – UV/Vis

(CH_2Cl_2): λ_{max} (lg ε) = 237 (3.93), 320 (2.35) nm. – HREIMS: calcd for $C_{13}H_{20}O_3$ $[M]^{\cdot+}$: 224.14070, found 224.14148.

Ethyl (*R*)-6-isopropyl-3-methyl-2-(((trifluoromethyl)sulfonyl)oxy)cyclohexa-1,3-diene-1-carboxylate (11)

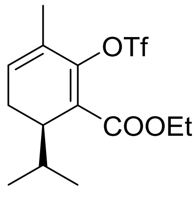

11

At −78 °C, a solution of LiHMDS in THF (1.0 M, 40.3 mL, 40.3 mmol) was added dropwise to a solution of **10** (9.023 g, 40.23 mmol) in dry THF (100 mL). It was stirred for 1.5 h at the same temperature, before Tf_2O (8.2 mL, 48.83 mmol) was added dropwise at −78 °C. After 5 min the reaction was quenched by addition of saturated aqueous NH_4Cl (50 mL) at –78 °C, and the reaction mixture was allowed to warm to room temperature. The aqueous layer was extracted with EtOAc (4 × 150 mL), the organic phase was dried over $MgSO_4$ and the solvent was removed under reduced pressure. After purification by column chromatography (silica, petroleum ether/EtOAc, 40:1), triflate **11** (12.01 g, 33.73 mmol, 84 %) was obtained as colorless oil. – R_f (petroleum ether/EtOAc, 20:1) = 0.40. – $[\alpha]_D^{22}$ = –3.3 (*c* = 1.0, $CHCl_3$). – ^1H NMR (400 MHz, $CDCl_3$): δ = 5.86 (m, 1H, CH_3C_qCH), 4.34 (qd, *J* = 7.2, 10.9 Hz, 1H, $CHHCH_3$), 4.21 (qd, *J* = 7.2, 10.9 Hz, 1H, $CHHCH_3$), 2.76 (tdd, *J* = 1.5, 7.1, 8.7 Hz, 1H, $(CH_3)_2CHCH$), 2.48 (m, 1H, CHC*H*HCH), 2.35 (dd, *J* = 6.5, 18.2 Hz, 1H, CHCH*H*CH), 1.84 (m, 3H, CH_3C_q), 1.83 (sept, *J* = 6.8 Hz, 1H, $(CH_3)_2CH$), 1.34 (t, *J* = 7.2 Hz, 3H, CH_3CH_2), 0.87 (d, *J* = 6.8 Hz, 3H, CH_3CHCH_3), 0.86 (d, *J* = 6.7 Hz, 3H, CH_3CHCH_3). – ^{13}C NMR (100 MHz, $CDCl_3$): δ = 165.8 (1C, C_qOOEt), 148.4 (1C, C_qOTf), 131.0 (1C, CH_3C_qCH), 127.8 (1C, CH_3C_q), 123.3 (1C, C_qC_qOOEt), 118.3 (q, 1C, $J(^{19}F,^{13}C)$ = 320.2 Hz, CF_3), 61.7 (1C, CH_2CH_3), 40.5 (1C, $(CH_3)_2CHCH$), 29.5 (1C, $(CH_3)_2CH$), 24.6 (1C, $CHCH_2CH$), 20.4 (1C, CH_3CHCH_3), 20.1 (1C, CH_3CHCH_3), 16.2 (1C, CH_3C_q), 13.8 (1C, CH_3CH_2). – IR (ATR): $\tilde{\nu}$ = 2965 (w), 1717 (m), 1424 (m), 1251 (m), 1203 (s), 1139 (s), 1024 (m), 931 (w), 860 (s), 672 (w), 606 (s) cm^{-1}. – UV/Vis (CH_2Cl_2): λ_{max} (lg ε) = 203 (3.83), 293 (3.75) nm. – MS (GC-MS, EI): *m/z* (%) = 356 (5) $[M]^+$, 311 (23), 267 (45), 241 (35), 181 (36), 134 (83), 91 (100), 77 (46), 53 (15). – HREIMS: calcd for $C_{14}H_{19}F_3O_5S$ $[M]^{\cdot+}$: 356.08998, found 356.08922.

(*R*)-2-Formyl-3-isopropyl-6-methylcyclohexa-1,5-dien-1-yl trifluoromethanesulfonate (15)

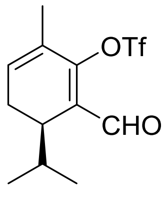

15

To a solution of **11** (2 g, 6.404 mmol) in DCM (40 mL), a solution of DIBAL-H in toluene (1.2 M, 13.3 mL, 15.96 mmol) was dropwise added at −78 °C and the resulting solution was stirred for 20 min at the same temperature. Water (3 mL) was added carefully and the reaction mixture was allowed to warm to rt. Na_2SO_4 (8 g) was added, and the reaction stirred for another 30 min. The reaction mixture was filtered and the filtrate was concentrated. The crude alcohol was dissolved in EtOAc (40 mL), IBX (3.228 g, 11.53 mmol) was added and resulting mixture was refluxed for 7 h. The mixture was cooled to room temperature, filtered and the filtrate concentrated under reduced pressure. Purification of the residue by column chromatography (silica, petroleum ether/EtOAc, 60:1) afforded **15** (1.894 g, 6.065 mmol, 95%) as yellow oil. R_f (petroleum ether/EtOAc, 20:1) = 0.42. – $[\alpha]_D^{23}$ = –2.3 (*c* = 0.9, $CHCl_3$). – ^1H NMR (400 MHz, $CDCl_3$): δ = 10.01 (s, 1H, CHO), 6.13

(m, 1H, CH_3C_qCH), 2.81 (m, 1H, $(CH_3)_2CHCH$), 2.44 (m, 1H, $CHCH_2CH$), 1.93 (ddd, J = 1.5, 1.5, 3.9 Hz, 3H, CH_3C_q), 1.81 (qd, J = 6.8, 13.6 Hz, 1H, $(CH_3)_2CH$), 1.34 (t, J = 7.2 Hz, 3H, CH_3CH_2), 0.85 (d, J = 6.8 Hz, 3H, CH_3CHCH_3), 0.83 (d, J = 6.9 Hz, 3H, CH_3CHCH_3). – ^{13}C NMR (100 MHz, $CDCl_3$): δ = 187.5 (1C, CHO), 155.9 (1C, C_qOTf), 136.6 (1C, CH_3C_qCH), 129.4 (1C, C_qCHO), 128.1 (1C, CH_3C_q), 118.5 (q, 1C, $J(^{19}F,^{13}C)$ = 320.4 Hz, CF_3), 36.2 (1C, $(CH_3)_2CHCH$), 29.4 (1C, $(CH_3)_2CH$), 24.8 (1C, $CHCH_2CH$), 20.6 (1C, CH_3CHCH_3), 19.6 (1C, CH_3CHCH_3), 16.2 (1C, CH_3C_q). – IR (ATR): $\tilde{\nu}$ = 3390 (br, w), 2965 (w), 1701 (w), 1405 (m), 1206 (s), 1135 (s), 1026 (m), 873 (m), 800 (w), 603 (m) cm^{-1}. – UV/Vis (CH_2Cl_2): λ_{max} (lg ε) = 223 (3.48), 244 (3.59), 290 (3.12), 356 (2.51) nm. – MS (GC-MS, EI): m/z (%) = 312 (5) $[M]^+$, 269 (28), 241 (8), 136 (45), 109 (80), 91 (100), 77 (25), 69 (30), 53 (12). – HREIMS: calcd for $C_{12}H_{15}F_3O_4S$ $[M]^{\cdot+}$: 312.06377, found 312.06483.

Ethyl (*R*,*Z*)-6-isopropyl-3-methyl-2-(3-methyl-6-(2-methyl-1,3-dioxolan-2-yl)hex-3-en-1-yn-1-yl)cyclohexa-1,3-diene-1-carboxylate (13)

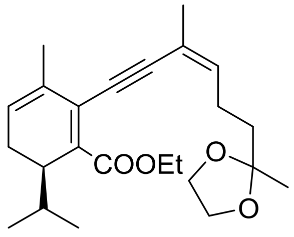

13

Under argon, triflate **11** (60 mg, 0.168 mmol), CuI (3.2 mg, 0.0168 mmol), and $PdCl_2(PPh_3)_2$ (12 mg, 0.0168 mmol) were dissolved in degassed Et_3N (2 mL). A solution of alkyne **12** (34 mg, 0.185 mmol) in degassed Et_3N (2 mL) was added dropwise at 0 °C. After 14 h at rt it was filtered over Celite and the solvent was removed under reduced pressure at 21 °C. After column chromatography (silica, petroleum ether/EtOAc, 20:1 to 10:1) product **13** (56 mg, 0.145 mmol, 86%) was obtained as yellow oil. – R_f (petroleum ether/EtOAc, 10:1) = 0.27. – $[\alpha]_D^{22}$ = –6.3 (c = 1.6, $CHCl_3$). – ^{1}H NMR (400 MHz, $CDCl_3$): δ = 5.72 (overlapping, 1H, C_qCHCH_2CH), 5.72 (overlapping, 1H, $CHCH_2CH_2$), 4.26 (m, 2H, CH_2CH_3), 3.93 (m, 4H, OCH_2CH_2O), 2.62 (dt, J = 4.0, 6.9 Hz, 1H, $(CH_3)_2CHCH$), 2.43 (m, 2H, $CHCH_2CH_2$), 2.31 (m, 2H, $CHCH_2CH$), 1.96 (dd, J = 1.8, 3.7 Hz, 3H, $CH_3C_qCHCH_2CH$), 1.91 (ddd, J = 1.2, 1.2, 1.2 Hz, 3H, $CH_3C_qCHCH_2CH_2$), 1.83 (dq, J = 6.8, 13.7 Hz, 1H, $(CH_3)_2CH$) 1.72 (m, 2H, $CHCH_2CH_2$), 1.33 (s, 3H, CH_3C_qCO), 1.31 (t, J = 6.8 Hz, 3H, CH_3CH_2), 0.86 (d, J = 6.8 Hz, 3H, CH_3CHCH_3), 0.84 (d, J = 6.7 Hz, 3H, CH_3CHCH_3). – ^{13}C NMR (100 MHz, $CDCl_3$): δ = 168.1 (1C, C_qOOEt), 138.6 (1C, $CHCH_2CH_2$), 135.2 (1C, C_qC_qOOEt), 131.3 (1C, $CH_3C_qCHCH_2CH$), 127.0 (1C, $C_qC_qC_q$OOEt), 125.3 (1C, C_qCHCH_2CH), 118.2 (1C, $C_qCHCH_2CH_2$), 109.8 (1C, CH_3C_qO), 97.9 (1C, $C_qC_qCHCH_2CH_2$), 91.1 (1C, $C_qC_qC_qCHCH_2CH_2$), 64.6 (2C, OCH_2CH_2O), 60.4 (1C, CH_2CH_3), 38.7 (1C, $(CH_3)_2CHCH$), 38.3 (1C, $CHCH_2CH_2$), 29.9 (1C, $(CH_3)_2CH$), 25.7 (1C, $CHCH_2CH_2$), 24.7 (1C, $CHCH_2CH$), 23.9 (1C, CH_3C_qO), 22.9 (1C, $CH_3C_qCHCH_2CH_2$), 20.8 (1C, CH_3CHCH_3), 20.4 (1C, $CH_3C_qCHCH_2CH$), 20.3 (1C, CH_3CHCH_3), 14.3 (1C, CH_3CH_2). – IR (ATR): $\tilde{\nu}$ = 2957 (m), 2874 (w), 1706 (s), 1447 (w), 1373 (m), 1228 (s), 1053 (s), 1026 (s), 862 (m), 816 (m) cm^{-1}. – UV/Vis (CH_2Cl_2): λ_{max} (lg ε) = 228 (3.87), 247 (3.83), 255 (3.83), 283 (3.70) nm. – HRESI(+)MS : calcd for $C_{24}H_{34}NaO_4$ $[M+Na]^+$: 409.23493, found 409.23511.

Ethyl (*R*,*Z*)-6-isopropyl-3-methyl-2-(3-methyl-7-oxooct-3-en-1-yn-1-yl)cyclohexa-1,3-diene-1-carboxylate (14)

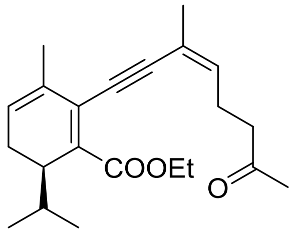

14

Under argon 1 M HCl (1.1 mL, 2.200 mmol) was added to a solution of **13** (52 mg, 0.135 mmol) in THF (5 mL) and it was stirred for 14 h at rt. Saturated aqueous $NaHCO_3$ (10 mL) was added and it was extracted with EtOAc (3 × 10 mL). The unified organic phases were washed with H_2O (2 × 15 mL) and brine (15 mL), dried over $MgSO_4$, and concentrated in vacuo at 21 °C. After column chromatography (silica, petroleum ether/EtOAc, 14:1 nach 10:1) product **14** (33 mg, 0.096 mmol, 71%) was obtained as yellow oil. – R_f (petroleum ether/EtOAc, 10:1) = 0.24. – $[\alpha]_D^{20}$ = –4.7 (*c* = 0.8, $CHCl_3$). – ^{1}H NMR (400 MHz, $CDCl_3$): δ = 5.73 (overlapping, 1H, C*H*CH_2CH_2), 5.72 (overlapping, 1H, C_qC*H*CH_2CH), 4.26 (m, 2H, C$H_2$$CH_3$), 2.62 (m, 1H, $(CH_3)_2$CHC*H*), 2.57 (m, 2H, CHCH_2CH), 2.55 (m, 2H, CHCH_2CH_2), 2.32 (m, 2H, CHCH_2CH), 2.16 (s, 3H, C$H_3$$C_q$=O), 1.93 (dd, *J* = 1.8, 3.7 Hz, 3H, C$H_3$$C_qCHCH_2$CH), 1.90 (dd, *J* = 1.2, 2,4 Hz, 3H, C$H_3$$C_qCHCH_2CH_2$), 1.83 (dq, *J* = 6.8, 13.7 Hz, 1H, $(CH_3)_2$C*H*), 1.31 (t, *J* = 7.1 Hz, 3H, C$H_3$$CH_2$), 0.86 (d, *J* = 6.8 Hz, 3H, CH_3CHCH_3), 0.84 (d, *J* = 6.7 Hz, 3H, CH_3CHCH_3). – ^{13}C NMR (100 MHz, $CDCl_3$): δ = 208.3 (1C, $CH_3$$C_q$=O), 167.9 (1C, C_qOOEt), 136.8 (1C, *C*HCH_2CH_2), 135.4 (1C, $C_q$$C_q$OOEt), 131.1 (1C, $CH_3$$C_qCHCH_2$CH), 127.0 (1C, $C_q$$C_q$$C_q$OOEt), 125.5 (1C, C_q*C*HCH_2CH), 119.5 (1C, C_qCHCH_2CH_2), 97.4 (1C, $C_q$$C_qCHCH_2CH_2$), 91.4 (1C, $C_q$$C_q$$C_qCHCH_2CH_2$), 60.4 (1C, C$H_2$$CH_3$), 43.1 (1C, CH$CH_2$*C*$H_2$), 38.6 (1C, $(CH_3)_2$CH*C*H), 29.9 (1C, $(CH_3)_2$*C*H), 29.7 (1C, *C*$H_3$$C_q$=O), 25.1 (1C, CH*C*$H_2CH_2$), 24.6 (1C, CH*C*$H_2$CH), 22.8 (1C, *C*$H_3$$C_qCHCH_2CH_2$), 20.8 (1C, *C*$H_3CHCH_3$), 20.3 (1C, *C*$H_3$$C_qCHCH_2$CH), 20.2 (1C, CH_3CH*C*H_3), 14.2 (1C, *C*$H_3$$CH_2$). – – MS (GC-MS, EI): *m*/*z* (%) = 342 (16) $[M]^+$, 299 (25), 253 (15), 213 (29), 185 (34), 169 (43), 154 (25), 141 (29), 128 (24), 115 (21), 91 (16), 43 (100). – IR (ATR): $\tilde{\nu}$ = 3414 (br, w), 2961 (w), 1712 (s), 1445 (w), 1366 (w), 1231 (s), 1166 (m), 1025 (m), 818 (w) cm^{-1}. – UV/Vis (CH_2Cl_2): λ_{max} (lg ε) = 228 (3.93), 256 (3.92), 284 (3.81) nm. – HREIMS: calcd for $C_{22}H_{30}O_3$ $[M]^{\cdot+}$: 342.21895, found 342.21797.

(*R*,*Z*)-6-Isopropyl-3-methyl-2-(3-methyl-6-(2-methyl-1,3-dioxolan-2-yl)hex-3-en-1-yn-1-yl)cyclohexa-1,3-diene-1-carbaldehyde (16)

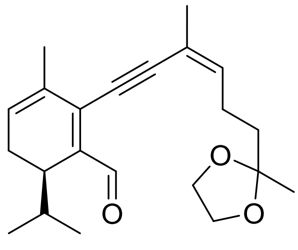

16

To a solution of triflate **15** (344 mg, 1.101 mmol), CuI (21 mg, 0.110 mmol), and $PdCl_2(PPh_{3)2}$ (77 mg, 0.110 mmol) in degassed THF (10 mL) was added alkyne **12** (218 mg, 1.211 mmol), dissolved in degassed Et_3N (5 mL), at 0 °C. After 14 h at rt it was filtered over Celite filtered and concentrated in vacuo at 22 °C. After column chromatography (silica, petroleum ether/EtOAc, 40:1 nach 15:1) product **16** (243 mg, 0.710 mmol, 64%) was obtained as yellow oil. – R_f (petroleum ether/EtOAc, 20:1) = 0.15. – $[\alpha]_D^{24}$ = +1.3 (*c* = 1.4, $CHCl_3$). – ^{1}H NMR (400 MHz, $CDCl_3$): δ = 10.32 (s, 1H, C*H*O), 5.93 (m, 1H, C_qC*H*CH_2CH), 5.82 (qt, *J* = 1.4, 7.5 Hz, 1H, C*H*CH_2CH_2), 3.92 (m, 4H, OCH_2CH_2O), 2.63 (m, 1H, $(CH_3)_2$CHC*H*), 2.41 (m, 2H, CHC$H_2$$CH_2$), 2.35 (m, 2H, CHC$H_2$CH), 1.97 (m, 3H, C$H_3$$C_qCHCH_2$CH), 1.90

(ddd, *J* = 1.2, 1.2, 1.2 Hz, 3H, $CH_3C_qCHCH_2CH_2$), 1.74 (m, 2H, $CHCH_2CH_2$), 1.73 (m, 1H, $(CH_3)_2CH$), 1.30 (s, 3H, CH_3C_qCO), 0.82 (d, *J* = 6.8 Hz, 3H, CH_3CHCH_3), 0.79 (d, *J* = 6.8 Hz, 3H, CH_3CHCH_3). – ^{13}C NMR (100 MHz, $CDCl_3$): δ = 192.8 (1C, *C*HO), 141.6 (1C, C_qCHO), 140.2 (1C, $CHCH_2CH_2$), 137.6 (1C, C_qC_qCHO), 131.5 (1C, $CH_3C_qCHCH_2CH$), 130.2 (1C, C_qCHCH_2CH), 117.6 (1C, $C_qCHCH_2CH_2$), 109.5 (1C, CH_3C_qO), 100.7 (1C, $C_qC_qCHCH_2CH_2$), 88.0 (1C, $C_qC_qC_qCHCH_2CH_2$), 64.7 (2C, OCH_2CH_2O), 38.3 (1C, $CHCH_2CH_2$), 34.4 (1C, $(CH_3)_2CHCH$), 29.5 (1C, $(CH_3)_2CH$), 25.9 (1C, $CHCH_2CH_2$), 24.8 (1C, $CHCH_2CH$), 24.0 (1C, CH_3C_qO), 22.7 (1C, $CH_3C_qCHCH_2CH_2$), 21.0 (1C, CH_3CHCH_3), 20.0 (1C, CH_3CHCH_3), 19.6 (1C, $CH_3C_qCHCH_2CH$). – IR (ATR): $\tilde{\nu}$ = 2957 (w), 2828 (w), 1659 (s), 1373 (m), 1207 (s), 1139 (w), 1053 (s), 863 (m), 817 (w), 607 (w), 566 (w) cm^{-1}. – UV/Vis (CH_2Cl_2): λ_{max} (lg ε) = 235 (3.84), 256 (3.77), 275 (3.73), 330 (3.33) nm. – HRESI(+)MS: calcd for $C_{22}H_{30}NaO_3$ $[M+Na]^+$: 365.20872, found 365.20885.

(*R*,*Z*)-6-Isopropyl-3-methyl-2-(3-methyl-7-oxooct-3-en-1-yn-1-yl)cyclohexa-1,3-diene-1-carbaldehyde (17)

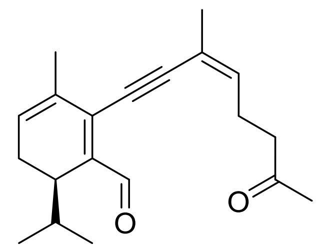

17

Under argon, 1 M HCl (2.5 mL, 4.995 mmol) was added to a solution of **16** (114 mg, 0.333 mmol) in THF (15 mL). After 16 h, saturated aqueous $NaHCO_3$ (20 mL) was added and it was extracted with EtOAc (4×20 mL). The unified organic phases were washed with H_2O (2×40 mL) and brine (40 mL), dried over $MgSO_4$, and concentrated in vacuo at 21 °C. After column chromatography (silica, petroleum ether/EtOAc, 10:1) product **17** (76 mg, 0.255 mmol, 77%) was obtained as yellow oil. – R_f (petroleum ether/EtOAc, 5:1) = 0.41. – $[\alpha]_D^{24}$ = –5.9 (*c* = 1.7, $CHCl_3$). – ^{1}H NMR (400 MHz, $CDCl_3$): δ = 10.31 (s, 1H, CHO), 5.96 (m, 1H, C_qCHCH_2CH), 5.81 (m, 1H, $CHCH_2CH_2$), 2.65 (m, 1H, $(CH_3)_2CHCH$), 2.55 (m, 2H, $CHCH_2CH$), 2.55 (m, 2H, $CHCH_2CH_2$), 2.42 (dd, *J* = 6.4, 18.5 Hz, 1H, $CHCHHCH_2$), 2.27 (m, 1H, $CHCHHCH_2$), 2.15 (s, 3H, CH_3C_q=O), 1.97 (m, 3H, $CH_3C_qCHCH_2CH$), 1.92 (dd, *J* = 0.9, 2.2 Hz, 3H, $CH_3C_qCHCH_2CH_2$), 1.73 (qd, *J* = 6.8, 13.6 Hz, 1H, $(CH_3)_2CH$), 0.84 (d, *J* = 6.8 Hz, 3H, CH_3CHCH_3), 0.81 (d, *J* = 6.9 Hz, 3H, CH_3CHCH_3). – ^{13}C NMR (100 MHz, $CDCl_3$): δ = 207.6 (C_q=O), 192.8 (1C, *C*HO), 141.8 (1C, C_qCHO), 138.3 (1C, $CHCH_2CH_2$), 137.5 (1C, C_qC_qCHO), 131.3 (1C, $CH_3C_qCHCH_2CH$), 130.4 (1C, C_qCHCH_2CH), 118.7 (1C, $C_qCHCH_2CH_2$), 100.2 (1C, $C_qC_qCHCH_2CH_2$), 88.3 (1C, $C_qC_qC_qCHCH_2CH_2$), 42.8 (1C, $CHCH_2CH_2$), 34.4 (1C, $(CH_3)_2CHCH$), 29.8 (1C, CH_3C_q=O), 29.5 (1C, $(CH_3)_2CH$), 25.3 (1C, $CHCH_2CH$), 24.8 (1C, $CHCH_2CH_2$), 22.7 (1C, $CH_3C_qCHCH_2CH_2$), 21.0 (1C, CH_3CHCH_3), 20.0 (1C, CH_3CHCH_3), 19.6 (1C, $CH_3C_qCHCH_2CH$). – IR (ATR): $\tilde{\nu}$ = 3470 (br, w), 2960 (m), 1713 (s), 1661 (s), 1369 (s), 1210 (m), 1163 (m), 1021 (w), 862 (m), 541 (m) cm^{-1}. – UV/Vis (CH_2Cl_2): λ_{max} (lg ε) = 224 (3.78), 236 (3.85), 256 (3.78), 275 (3.73), 330 (3.35) nm. – MS (GC-MS, EI): *m/z* (%) = 298 (10) $[M]^+$, 255 (100), 197 (14), 128 (13), 91 (7). – HREIMS: calcd for $C_{20}H_{26}O_2$ $[M]^{\cdot+}$: 298.19273, found 298.19402.

Bicyclic diol 18

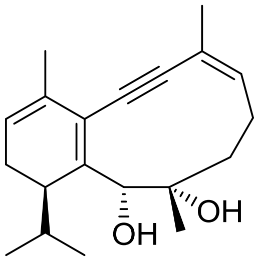

18

Under argon at 0 °C, zinc powder (261.6 mg, 4.000 mmol) was added dropwise to a solution of $TiCl_4$ (1.0 M in DCM, 2.0 mL, 2.000 mmol) in THF (37 mL). After 15 min a solution of ketoaldehyde **17** (30 mg, 0.101 mmol) in THF (8 mL) was added over a period of 2.5 h, followed by stirring (30 min). Aqueous K_2CO_3 (40 mL, 10%) was added an it was extracted with EtOAc (4 × 50 mL). The unified organic phases were dried over $MgSO_4$ and concentrated in vacuo at 20 °C. After separation by semipreparative HPLC (silica, hexane/*i*PrOH, 25:1) diol **18** (14 mg, 0.047 mmol, 47%) was obtained as yellow oil. – R_f (hexane/*i*PrOH, 25:1) = 0.17. – $[\alpha]_D^{25}$ = –5.0 (*c* = 1.4, $CHCl_3$). – ^1H NMR (600 MHz, $CDCl_3$): δ = 6.17 (m, 1H, $CH_3C_qCHCH_2CH_2$), 5.46 (m, 1H, $CH_3C_qCHCH_2CH$), 5.18 (s, 1H, CHOH), 2.82 (d, *J* = 3.1 Hz, 1H, CHO*H*), 2.46 (s, 1H, C_qOH), 2.43 (m, 1H, $(CH_3)_2CHCH$), 2.37 (m, 1H, CHC*H*HCH$_2$), 2.28 (m, 1H, CHC*H*HCH), 2.25 (m, 1H, CHCH*H*CH$_2$), 2.17 (m, 1H, CHCH*H*CH), 2.10 (m, 1H, $(CH_3)_2CH$), 2.00 (dd, *J* = 9.4, 14.7 Hz, 1H, $CHCH_2CHH$), 1.86 (ddd, *J* = 0.8, 1.4, 2.5 Hz, 3H, $CH_3C_qCHCH_2CH$), 1.83 (m, 3H, $CH_3C_qCHCH_2CH_2$), 1.75 (ddd, *J* = 9.4, 9.4 14.6 Hz, 1H, $CHCH_2CHH$), 1.20 (s, 3H, CH_3C_qOH), 0.93 (d, *J* = 7.0 Hz, 3H, CH_3CHCH_3), 0.83 (d, *J* = 6.9 Hz, 3H, CH_3CHCH_3). – ^{13}C NMR (150 MHz, $CDCl_3$): δ = 149.8 (1C, C_qC_qCHOH), 138.4 (1C, $CHCH_2CH_2$), 130.4 (1C, $CH_3C_qCHCH_2CH$), 122.2 (1C, $CH_3C_qCHCH_2CH_2$), 121.6 (1C, $CHCH_2CH$), 119.6 (1C, C_qC_qCHOH), 100.2 (1C, $C_qC_qCHCH_2CH_2$), 98.1 (1C, $C_qC_qC_qCHCH_2CH_2$), 73.1 (1C, CHOH), 71.6 (1C, C_qOH), 38.3 (1C, $(CH_3)_2CHCH$), 36.3 (1C, $CHCH_2CH_2$), 31.2 (1C, $(CH_3)_2CH$), 25.2 (1C, CH_3C_qOH), 24.1 (1C, $CHCH_2CH$), 23.7 (1C, $CHCH_2CH$), 21.8 (1C, CH_3CHCH_3), 20.4 (1C, $CH_3C_qCHCH_2CH_2$), 19.8 (1C, CH_3CHCH_3), 19.7 (1C, $CH_3C_qCHCH_2CH$). – IR (ATR): $\tilde{\nu}$ = 3421 (br, w), 2926 (w), 1452 (w), 1369 (w), 1046 (w), 753 (s) cm^{-1}. – UV/Vis (CH_2Cl_2): λ_{max} (lg ε) = 221 (3.65), 228 (3.73), 250 (3.68), 291 (3.51) nm. – MS (GC-MS, EI): *m/z* (%) = 300 (12) $[M]^+$, 282 (14), 242 (20), 229 (100), 199 (23), 181 (55), 171 (47), 155 (36), 143 (54), 115 (46), 105 (44), 91 (32), 77 (41). – HREIMS: calcd for $C_{20}H_{28}O_2$ $[M]^{\cdot+}$: 300.20838, found 300.20838.

Bicyclic diol 19

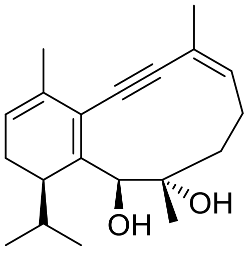

19

Under argon, IBX (57 mg, 0.202 mmol) was added to a solution of diol **18** (36 mg, 0.120 mmol) in CH_3CN (40 mL). After 4 h at rt the suspension was filtered over a pad of silica, followed by evaporation in vacuo at 21 °C. The crude product (13.2 mg, 44.232 µmol) was dissolved in THF (1.5 mL). At 0 °C, $LiAlH_4$ (1.0 M in THF, 22 µL, 22 µmol) was added. After 10 min H_2O (10 mL) and EtOAc (10 mL) were added, phases were separated and the aqueous phase was extracted with EtOAc (3 × 10 mL). The unified organic phases were with brine (1 × 20 mL) dried over Na_2SO_4, filtered and concentrated in vacuo at 21 °C. After column chromatography (silica, petroleum ether/EtOAc, 6:1) product **19** (12.8 mg, 42.604 µmol, 36%) was obtained as colorless solid. – R_f (petroleum ether/EtOAc, 5:1) = 0.43. –

$[\alpha]_D^{20}$ = –6.9 (c = 1.0, $CHCl_3$). – ^1H NMR (600 MHz, $CDCl_3$): δ = 6.08 (m, 1H, $CH_3C_qCHCH_2CH_2$), 5.49 (m, 1H, $CH_3C_qCHCH_2CH$), 4.14 (d, J = 9.6 Hz, 1H, CHOH), 3.66 (d, J = 9.3 Hz, 1H, CHO*H*), 3.20 (br s, 1H, $CHCHHCH_2$), 3.07 (s, 1H, C_qOH), 2.69 (dd, J = 8.3, 13.3 Hz, 1H, $CHCH_2CHH$), 2.25 (m, 2H, $CHCH_2CH$), 2.19 (m, 1H, $CHCHHCH_2$), 1.95 (m, 1H, $CHCH_2CHH$), 1.93 (m, 1H, $(CH_3)_2CH$), 1.85 (ddd, J = 1.0, 1.4, 2.7 Hz, 3H, $CH_3C_qCHCH_2CH$), 1.84 (m, 1H, $(CH_3)_2CHCH$), 1.80 (dd, J = 1.8, 2.1 Hz, 3H, $CH_3C_qCHCH_2CH_2$), 1.25 (s, 3H, CH_3C_qOH), 0.98 (d, J = 6.9 Hz, 3H, CH_3CHCH_3), 0.84 (d, J = 6.7 Hz, 3H, CH_3CHCH_3). – ^{13}C NMR (150 MHz, $CDCl_3$): δ = 149.0 (1C, C_qC_qCHOH), 139.2 (1C, $CHCH_2CH_2$), 129.9 (1C, $CH_3C_qCHCH_2CH$), 121.3 (1C, $CHCH_2CH$), 120.8 (1C, $CH_3C_qCHCH_2CH_2$), 118.6 (1C, C_qC_qCHOH), 100.7 (1C, $C_qC_qCHCH_2CH_2$), 96.6 (1C, $C_qC_qC_qCHCH_2CH_2$), 87.2 (1C, CHOH), 71.0 (1C, C_qOH), 46.4 (1C, $(CH_3)_2CHCH$), 37.2 (1C, $CHCH_2CH_2$), 29.4 (1C, $(CH_3)_2CH$), 28.4 (1C, CH_3C_qOH), 24.4 (1C, $CHCH_2CH$), 23.6 (1C, $CHCH_2CH$), 21.3 (1C, CH_3CHCH_3), 20.5 (1C, CH_3CHCH_3), 20.5 (1C, $CH_3C_qCHCH_2CH_2$), 19.6 (1C, $CH_3C_qCHCH_2CH$). – MS (GC-MS, EI): m/z (%) = 300 (11) $[M]^+$, 282 (14), 242 (22), 229 (88), 199 (19), 181 (35), 171 (55), 155 (31), 143 (52), 128 (47), 115 (39), 105 (43), 91 (32), 77 (41). – IR (ATR): $\tilde{\nu}$ = 3419 (br, w), 2932 (w), 1048 (m), 743 (s) cm^{-1}. – UV/Vis (CH_2Cl_2): λ_{max} (lg ε) = 221 (3.63), 229 (3.73), 251 (3.69), 290 (3.49) nm. – HREIMS: calcd for $C_{20}H_{28}O_2$ $[M]^{\cdot+}$: 300.20838, found 300.20822.

(2*R*,5*S*)-2-((*tert*-Butyldimethylsilyl)oxy)-5-isopropyl-2-methylcyclohexan-1-one (21)

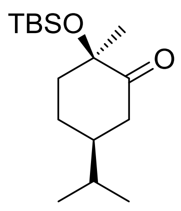

21

PtO_2 (89 mg, 0.393 mmol) was added to a solution of diol **20** (6.683 g, 39.254 mmol) in MeOH (150 mL) and stirred in an H_2 atmosphere (balloon) for 3 h. The suspension was filtered over Celite filtered and the solvent was removed in vacuo. The crude product was dissolved in EtOAc, IBX (13.190 g, 47.105 mmol) was added and it was refluxed for 5 h. After cooling the suspension was filtered over a pad silica and concentrated in vacuo. The crude product was dissolved in DCM (50 mL) and 2,6-lutidine (1.1 mL, 10.4 mL, 90.284 mmol) was added, followed by dropwise addition of TBSOTf (12.6 mL, 54.956 mmol) at −78 °C. The cooling was removed and it was stirred for 1 h, before ice-cold 1 M HCl (20 mL) was added. Phases were separated and the organic phase was washed with brine (2 × 20 mL). It was dried over Na_2SO_4 and concentrated in vacuo. After column chromatography (silica, petroleum ether/EtOAc, 30:1) product **21** (9.074 g, 31.893 mmol, 81%) was obtained as colorless oil. – R_f (petroleum ether/EtOAc, 20:1) = 0.44. – $[\alpha]_D^{24}$ = –56.8 (c = 2.4, $CHCl_3$). – ^1H NMR (400 MHz, $CDCl_3$): δ = 2.73 (dd, J = 12.4, 12.4 Hz, 1H, $CHHC_q$=O), 2.19 (ddd, J = 2.3, 3.6, 12.2 Hz, 1H, $CHHC_q$=O), 2.03 (ddd, J = 2.9, 3.5, 14.0 Hz, 1H, $TBSOC_qCHH$), 1.78 (dddd, J = 3.5, 13.2, 13.3, 16.5 Hz, 1H, $TBSOC_qCH_2CHH$), 1.55 (m, 1H, $(CH_3)_2CH$), 1.50 (m, 1H, $TBSOC_qCH_2CHH$),1.49 (m, 1H, $(CH_3)_2CHCH$), 1.40 (ddd, J = 3.9, 13.4, 13.8 Hz, 1H, $TBSOC_qCHH$), 1.29 (s, 3H, $CH_3C_qC_q$=O), 0.91 (d, J = 6.7 Hz, 3H, CH_3CHCH_3), 0.91 (s, 9H, $SiC_q(CH_3)_3$), 0.83 (d, J = 6.9 Hz, 3H, CH_3CHCH_3), 0.11 (s, 3H, CH_3SiCH_3), 0.01 (s, 3H, CH_3SiCH_3). – ^{13}C NMR (100 MHz, $CDCl_3$): δ = 212.6 (1C, C_q=O), 77.4 (1C, $TBSOC_q$), 46.8 (1C, $(CH_3)_2CHCH$), 41.8 (1C, $TBSOC_qCH_2$), 41.1 (1C, CH_2C_q=O), 32.8 (1C, $(CH_3)_2CH$), 25.9 (3C, $SiC_q(CH_3)_3$), 23.9 (1C, $TBSOC_qCH_2CH_2$), 23.6 (1C, CH_3C_qOTBS), 19.6 (1C, CH_3CHCH_3), 19.4 (1C, CH_3CHCH_3), 18.3 (1C, SiC_q), -2.0 (1C, CH_3SiCH_3), -3.2 (1C, CH_3SiCH_3). – IR (ATR): $\tilde{\nu}$ = 2956 (w), 2932 (w), 2858 (w), 1721 (m), 1468 (w), 1372 (w), 1254 (w), 1207 (w), 1169 (m), 1138 (w), 1081 (m), 1028 (m), 967 (w), 937 (w), 869 (w), 831 (s), 774 (s), 732 (w), 679 (w), 535 (w) cm^{-1}. – UV/Vis

(CH_2Cl_2): λ_{max} (lg ε) = 228 (2.21), 299 (1.74) nm. – MS (GC-MS, EI): *m/z* (%) = 269 (3) $[M–CH_3]^+$, 227 (100), 185 (10), 143 (19), 123 (18), 115 (12), 75 (89). – HREIMS: calcd for $C_{16}H_{32}O_2Si$ $[M–CH_3]^+$: 269.19319, found 269.19112.

Ethyl (1*R*,2*S*,3*R*,6*R*)-3-((*tert*-butyldimethylsilyl)oxy)-2-cyano-2-hydroxy-6-isopropyl-3-methylcyclohexane-1-carboxylate (22)

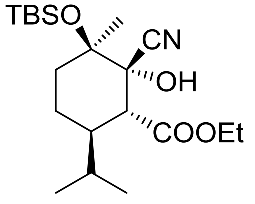

22

Under argon at −78 °C LiHMDS (1.0 M in THF, 1.1 mL, 1.100 mmol) was added to a solution of ketone **21** (150 mg, 0.527 mmol) in THF (2 mL) and warmed up to rt within 30 min. A solution of CNCOOEt (209 mg, 2.109 mmol) in THF (0.5 mL) was added dropwise at −78 °C and it was stirred for 30 min. Saturated aqueous NH_4Cl (5 mL) and EtOAc (5 mL) were added. At rt the phases were separated and the aqueous phase was extracted with EtOAc (3 × 5 mL). The unified organic phases were dried over Na_2SO_4, filtered, and the solvent was removed in vacuo. After column chromatography (silica, petroleum ether/EtOAc, 40:1 to 30:1) product **22** (154 mg, 0.401 mmol, 76%) was obtained as colorless oil. – R_f (petroleum ether/EtOAc, 20:1) = 0.31. – $[\alpha]_D^{22}$ = –8.1 (*c* = 1.9, $CHCl_3$). – $[\alpha]_D^{22}$ = –18.0 (*c* = 2.6, $CHCl_3$). – ^1H NMR (400 MHz, $CDCl_3$): δ = 5.19 (s, 1H, O*H*), 4.24 (dq, *J* = 0.6, 7.1 Hz, 2H, C*H*$_2$CH$_3$), 3.13 (d, *J* = 12.1 Hz, 1H, C*H*C$_q$COOEt), 1.84 (m, 1H, (CH$_3$)$_2$CHC*H*), 1.77 (m, 1H, TBSOC$_q$C*H*H), 1.60 (td, *J* = 3.4, 14.1 Hz, 1H, TBSOC$_q$CH*H*), 1.50 (m, 1H, (CH$_3$)$_2$C*H*), 1.46 (s, 3H, C*H*$_3$C$_q$OTBS), 1.42 (m, 2H, TBSOC$_q$CH$_2$C*H*$_2$), 1.34 (t, *J* = 7.1 Hz, 3H, C*H*$_3$CH$_2$), 0.94 (s, 9H, SiC$_q$(C*H*$_3$)$_3$), 0.92 (d, *J* = 6.9 Hz, 3H, C*H*$_3$CHCH$_3$), 0.86 (d, *J* = 6.9 Hz, 3H, CH$_3$CHC*H*$_3$), 0.21 (s, 3H, CH$_3$SiC*H*$_3$), 0.14 (s, 3H, C*H*$_3$SiCH$_3$). – ^{13}C NMR (100 MHz, $CDCl_3$): δ = 176.3 (1C, *C*$_q$OOEt), 120.3 (1C, *C*N), 76.2 (1C, *C*$_q$CN), 74.7 (1C, TBSO*C*$_q$), 61.7 (1C, *C*H$_2$CH$_3$), 49.2 (1C, *C*HC$_q$OOEt), 39.2 (1C, (CH$_3$)$_2$CH*C*H), 33.0 (1C, TBSOC$_q$*C*H$_2$), 29.2 (1C, (CH$_3$)$_2$*C*H), 25.9 (3C, SiC$_q$(*C*H$_3$)$_3$), 24.8 (1C, *C*H$_3$C$_q$OTBS), 21.0 (1C, *C*H$_3$CHCH$_3$), 18.3 (1C, Si*C*$_q$), 18.1 (1C, TBSOC$_q$CH$_2$*C*H$_2$), 15.5 (1C, CH$_3$CH*C*H$_3$), 14.1 (1C, *C*H$_3$CH$_2$), -2.0 (1C, *C*H$_3$SiCH$_3$), -2.4 (1C, CH$_3$Si*C*H$_3$). – IR (ATR): $\tilde{\nu}$ = 3425 (br, w), 2957 (w), 2933 (w), 2889 (w), 2859 (w), 1702 (m), 1467 (w), 1375 (w), 1332 (w), 1256 (m), 1147 (m), 1093 (m), 1065 (w), 1043 (m), 976 (w), 943 (w), 902 (w), 836 (s), 775 (s), 721 (w), 684 (w), 656 (w), 594 (w), 542 (w) cm^{-1}. – UV/Vis (CH_2Cl_2): λ_{max} (lg ε) = 228 (2.29). – MS (GC-MS, EI): *m/z* (%) = 326 (14) $[M–HCN–C_2H_6]^+$, 299 (74), 253 (19), 225 (76), 211 (14), 185 (27), 151 (29), 115 (26), 75 (100), 59 (14). – HREIMS: calcd for $C_{17}H_{30}O_4Si$ $[M–C_4H_9]^+$: 326.17821, found 326.17640.

Ethyl (1*R*,3*R*,6*R*)-3-((*tert*-butyldimethylsilyl)oxy)-6-isopropyl-3-methyl-2-oxocyclohexane-1-carboxylate (23)

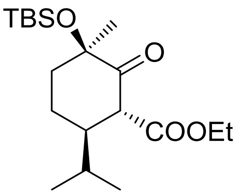

23

To a solution of cyanohydrin **22** (921 mg, 2.382 mmol) in Et_2O (45 mL) was added 1 M NaOH (3.7 mL, 3.700 mmol) under vigorous stirring. H_2O (35 mL) was added and it was extracted with Et_2O (4×40 mL). The unified organic phases were washed with H_2O (2 × 100 mL) and brine (100 mL) and dried over $MgSO_4$. The solvent was removed in vacuo and ketoester **23** (849 mg, 2.381 mmol, 100%) was obtained as colorless oil. – R_f (petroleum ether/EtOAc,

20:1) = 0.31. – $[\alpha]_D^{22}$ = –42.1 (*c* = 2.0, $CHCl_3$). – ^{1}H NMR (400 MHz, $CDCl_3$): *δ* = 4.26 (qd, *J* = 7.1, 7.3 Hz, 1H, $CHHCH_3$), 4.19 (qd, *J* = 7.1, 7.3 Hz, 1H, $CHHCH_3$), 3.96 (d, *J* = 12.5 Hz, 1H, CHC_qCOOEt), 2.06 (m, 1H, $(CH_3)_2CHCH$), 2.04 (m, 1H, $TBSOC_qCHH$), 1.80 (m, 1H, $TBSOC_qCH_2CHH$), 1.71 (m, 1H, $(CH_3)_2CH$), 1.47 (m, 1H, $TBSOC_qCH_2CHH$), 1.45 (m, 1H, $TBSOC_qCHH$), 1.30 (s, 3H, CH_3C_qOTBS), 1.27 (t, *J* = 7.1 Hz, 3H, CH_3CH_2), 0.94 (d, *J* = 6.9 Hz, 3H, CH_3CHCH_3), 0.92 (s, 9H, $SiC_q(CH_3)_3$), 0.86 (d, *J* = 7.0 Hz, 3H, CH_3CHCH_3), 0.11 (s, 3H, CH_3SiCH_3), 0.05 (s, 3H, CH_3SiCH_3). – ^{13}C NMR (100 MHz, $CDCl_3$): *δ* = 207.3 (1C, $TBSOC_qC_q$=O), 170.1 (1C, C_qOOEt), 77.4 (1C, $TBSOC_q$), 60.6 (1C, CH_2CH_3), 57.4 (1C, CHC_qOOEt), 47.4 (1C, $(CH_3)_2CHCH$), 41.0 (1C, $TBSOC_qCH_2$), 29.8 (1C, $(CH_3)_2CH$), 25.9 (3C, $SiC_q(CH_3)_3$), 23.7 (1C, CH_3C_qOTBS), 21.2 (1C, CH_3CHCH_3), 18.2 (1C, $TBSOC_qCH_2CH_2$), 18.2 (1C, SiC_q), 15.8 (1C, CH_3CHCH_3), 14.2 (1C, CH_3CH_2), -2.1 (1C, CH_3SiCH_3), -3.4 (1C, CH_3SiCH_3). – IR (ATR): $\tilde{\nu}$ = 2957 (w), 2934 (w), 2859 (w), 1747 (m), 1720 (m), 1467 (w), 1371 (w), 1330 (w), 1254 (m), 1216 (w), 1174 (w), 1142 (m), 1085 (m), 1015 (m), 987 (w), 946 (w), 833 (s), 775 (s), 725 (w), 673 (w), 637 (w), 540 (w) cm^{-1}. – UV/Vis (CH_2Cl_2): λ_{max} (lg *ε*) = 226 (2.40), 251 (2.44) nm. – MS (GC-MS, EI): *m/z* (%) = 341 (2) $[M–CH_3]^+$, 299 (99), 271 (40), 253 (23), 225 (97), 211 (14), 185 (42), 151 (33), 129 (17), 115 (24), 59 (14), 75 (98), 73 (100). – HREIMS: calcd for $C_{19}H_{36}O_4Si$ $[M–CH_3]^+$: 341.21426, found 341.21189.

Ethyl (1*R*,2*R*,3*R*,6*R*)-3-((*tert*-butyldimethylsilyl)oxy)-2-hydroxy-6-isopropyl-3-methyl-2-((*Z*)-3-methyl-6-(2-methyl-1,3-dioxolan-2-yl)hex-3-en-1-yn-1-yl)cyclohexane-1-carboxylate (24)

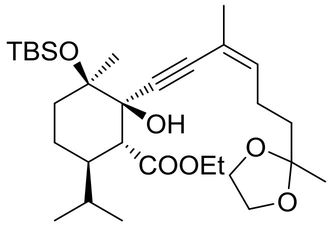

24

Under argon at −25 °C *n*-BuLi (1.6 M in hexane, 2.3 mL, 3.693 mmol) was added to a solution of alkyne **12** (634 mg, 3.517 mmol) in THF (20 mL) and it was stirred for 10 min. It was cooled to −78 °C and a solution of ketoester **23** (1.505 g, 4.221 mmol) in THF (20 mL) was added dropwise over 2 h, followed by stirring for 10 min. H_2O (40 mL) and EtOAc (50 mL) were added, phases were separated and the aqueous phase was extracted with EtOAc (3 × 10 mL). The unified organic phases were washed with brine (1 × 50 mL) dried over Na_2SO_4, filtered and concentrated in vacuo. After column chromatography (silica, petroleum ether/EtOAc, 10:1) product **24** (1.189 g, 2.215 mmol, 63%, 77% based on recovered alkyne **12**) was obtained as colorless oil. – R_f (petroleum ether/EtOAc, 5:1) = 0.29. – $[\alpha]_D^{20}$ = –32.7 (*c* = 2.8, $CHCl_3$). – ^{1}H NMR (400 MHz, $CDCl_3$): *δ* = 5.66 (qt, *J* = 1.3, 7.3 Hz, 1H, $CH_3C_qCHCH_2CH_2$), 4.21 (qd, *J* = 7.1, 10.8 Hz, 1H, $CHHCH_3$), 4.14 (qd, *J* = 7.1, 10.8 Hz, 1H, $CHHCH_3$), 3.92 (m, 4H, OCH_2CH_2O), 2.89 (d, *J* = 11.9 Hz, 1H, CHC_qCOOEt), 2.61 (s, 1H, OH), 2.37 (m, 2H, $CH_3C_qCHCH_2CH_2$), 1.97 (m, 1H, $(CH_3)_2CHCH$), 1.87 (m, 1H, $TBSOC_qCHH$), 1.84 (dd, *J* = 1.2, 2.5 Hz, 3H, $CH_3C_qCHCH_2CH_2$), 1.71 (m, 1H, $(CH_3)_2CH$), 1.70 (m, 1H, $TBSOC_qCHH$), 1.70 (m, 2H, $CH_3C_qCHCH_2CH_2$), 1.47 (s, 3H, CH_3C_qOTBS), 1.39 (m, 2H, $TBSOC_qCH_2CH_2$), 1.33 (s, 3H, $CH_3C_qCOCH_2CH_2O$), 1.25 (t, *J* = 7.1 Hz, 3H, CH_3CH_2), 0.93 (s, 9H, $SiC_q(CH_3)_3$), 0.91 (d, *J* = 6.9 Hz, 3H, CH_3CHCH_3), 0.80 (d, *J* = 6.9 Hz, 3H, CH_3CHCH_3), 0.16 (s, 6H, CH_3SiCH_3). – ^{13}C NMR (100 MHz, $CDCl_3$): *δ* = 172.7 (1C, C_qOOEt), 137.6 (1C, $CH_3C_qCHCH_2CH_2$), 117.7 (1C, $CH_3C_qCHCH_2CH_2$), 109.8 (1C, $CH_3C_qCOCH_2CH_2O$), 91.3 (1C, $HOC_qC_qC_q$), 87.2 (1C, $HOC_qC_qC_q$), 78.4 (1C, $TBSOC_q$), 76.7 (1C, C_qOH), 60.0 (1C, CH_2CH_3), 54.3 (1C, CHC_qOOEt), 64.6 (1C, OCH_2CH_2O), 64.6 (1C, OCH_2CH_2O), 42.3 (1C, $(CH_3)_2CHCH$), 38.3 (1C, $CH_3C_qCHCH_2CH_2$), 36.0 (1C, $TBSOC_qCH_2$), 28.8 (1C, $(CH_3)_2CH$), 26.1 (3C,

$SiC_q(CH_3)_3$), 25.6 (1C, $CH_3C_qCHCH_2CH_2$), 24.4 (1C, CH_3C_qOTBS), 23.9 (1C, $CH_3C_qCOCH_2CH_2O$), 22.9 (1C, $CH_3C_qCHCH_2CH_2$), 21.4 (1C, CH_3CHCH_3), 18.5 (1C, SiC_q), 18.3 (1C, $TBSOC_qCH_2CH_2$), 15.8 (1C, CH_3CHCH_3), 14.3 (1C, CH_3CH_2), -1.9 (1C, CH_3SiCH_3), -2.5 (1C, CH_3SiCH_3). – IR (ATR): $\tilde{\nu}$ = 3454 (br, w), 2954 (s), 2883 (m), 1736 (m), 1467 (w), 1373 (w), 1251 (m), 1147 (m), 1093 (m), 1053 (s), 834 (s), 776 (m) cm^{-1}. – UV/Vis (CH_2Cl_2): 232 (4.13) nm. – HRESI(+)MS: calcd for $C_{30}H_{52}NaO_6Si$ $[M+Na]^+$: 559.34254, found 559.34266.

(1*R*,2*R*,3*R*,6*R*)-3-((*tert*-Butyldimethylsilyl)oxy)-2-hydroxy-6-isopropyl-3-methyl-2-((*Z*)-3-methyl-7-oxooct-3-en-1-yn-1-yl)cyclohexane-1-carbaldehyde (25)

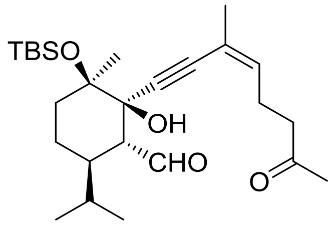

25

Under argon at −78 °C DIBAL-H (1.2 M in toluene, 3.0 mL, 3.600 mmol) was added to a solution of **24** (868 mg, 1.617 mmol) in DCM (25 mL). It was warmed to rt within 1 h and stirred for 14 h. H_2O (2 mL) and Na_2SO_4 (6 g) were added and it was stirred for 30 min. After filtration over Celite the solvent was removed in vacuo. The crude product was dissolved in DMSO (3 mL, argon atmosphere) and IBX (588 mg, 2.102 mmol) was added. After 2 h at rt H_2O (3 mL) was added, and after 30 min it was filtered over Celite. Phases were separated and the aqueous phase was extracted with TBME (3 × 10 mL). The unified organic phases were washed with H_2O (2 × 5 mL) and brine (1 × 5 mL), dried over Na_2SO_4 and filtered. The solvent was removed in vacuo and a solution of the crude product in THF (15 mL) was treated with 1 M HCl (12.2 mL, 24.4 mmol) for 14 h at rt. Saturated aqueous $NaHCO_3$ (20 mL) was added and it was extracted with EtOAc (4 × 20 mL). The unified organic phases were washed with H_2O (2 × 30 mL) and brine (30 mL), dried over Na_2SO_4, and the solvent was removed. After column chromatography (silica, petroleum ether/EtOAc, 10:1) product **25** (511 mg, 1.139 mmol, 71%) was obtained as yellowish oil. – R_f (petroleum ether/EtOAc, 5:1) = 0.59. – $[\alpha]_D^{21}$ = –3.9 (*c* = 1.3, $CHCl_3$). – ^1H NMR (400 MHz, $CDCl_3$): δ = 9.83 (d, *J* = 5.4 Hz, 1H, CHO), 5.71 (qd, *J* = 1.3, 7.0 Hz, 1H, $C_qCHCH_2CH_2$), 2.59 (overlapping, 1H, OH), 2.57 (dd, *J* = 5.4, 11.9 Hz, 1H, $CHCHO$), 2.51 (m, 2H, $O{=}C_qCH_2CH_2$), 2.48 (m, 2H, $O{=}C_qCH_2CH_2$), 2.14 (s, 3H, $CH_3C_q{=}O$), 2.06 (ddt, *J* = 3.0, 4.5, 11.8 Hz, 1H, $(CH_3)_2CHCH$), 1.84 (dd, *J* = 1.0, 2.3 Hz, 3H, $CH_3C_qCHCH_2CH_2$), 1.81 (m, 2H, $TBSOC_qCH_2$), 1.64 (m, 1H, $(CH_3)_2CH$), 1.46 (s, 3H, CH_3C_qOTBS), 1.44 (m, 2H, $TBSOC_qCH_2CH_2$), 0.94 (d, *J* = 6.9 Hz, 3H, CH_3CHCH_3), 0.92 (s, 9H, $SiC_q(CH_3)_3$), 0.79 (d, *J* = 6.9 Hz, 3H, CH_3CHCH_3), 0.17 (s, 3H, CH_3SiCH_3), 0.16 (s, 3H, CH_3SiCH_3). – ^{13}C NMR (100 MHz, $CDCl_3$): δ = 207.8 (1C, $C_q{=}O$), 205.3 (1C, CHO), 136.9 (1C, $CH_3C_qCHCH_2CH_2$), 118.4 (1C, $CH_3C_qCHCH_2CH_2$), 90.8 (1C, $HOC_qC_qC_q$), 88.5 (1C, $HOC_qC_qC_q$), 78.0 (1C, $TBSOC_q$), 75.5 (1C, C_qOH), 59.4 (1C, $CHCHO$), 42.8 (1C, $O{=}C_qCH_2CH_2$), 40.9 (1C, $(CH_3)_2CHCH$), 35.9 (1C, $TBSOC_qCH_2$), 29.9 (1C, $CH_3C_q{=}O$), 28.8 (1C, $(CH_3)_2CH$), 26.1 (3C, $SiC_q(CH_3)_3$), 25.1 (1C, $O{=}C_qCH_2CH_2$), 24.1 (1C, CH_3C_qOTBS), 22.8 (1C, $CH_3C_qCHCH_2CH_2$), 21.1 (1C, CH_3CHCH_3), 18.5 (1C, SiC_q), 18.3 (1C, $TBSOC_qCH_2CH_2$), 15.7 (1C, CH_3CHCH_3), -1.9 (1C, CH_3SiCH_3), -2.5 (1C, CH_3SiCH_3). – IR (ATR): $\tilde{\nu}$ = 3551 (br, w), 2954 (m), 2934 (m), 1736 (s), 1467 (w), 1373 (m), 1251 (m), 1201 (m), 1147 (s), 1093 (s), 1053 (s), 834 (s), 776 (s) cm^{-1}. – UV/Vis (CH_2Cl_2): 231 (3.34), 270 (2.77) nm. – HRESI(+)MS: calcd for $C_{26}H_{44}NaO_4Si$ $[M+Na]^+$: 471.29011, found 471.29039.

(1*R*,2*R*,3*R*,6*R*)-3-((*tert*-Butyldimethylsilyl)oxy)-6-isopropyl-3-methyl-2-((*Z*)-3-methyl-7-oxooct-3-en-1-yn-1-yl)-2-((trimethylsilyl)oxy)cyclohexane-1-carbaldehyde (26)

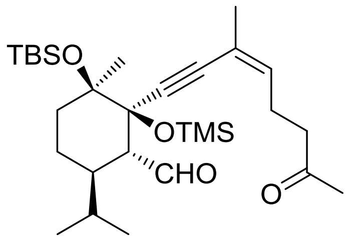

26

Under argon 2,6-lutidine (23 µL, 0.200 mmol) was added to a solution of alcohol **25** (45 mg, 0.100 mmol) in DCM (2 mL). TMSOTf (36 µL, 0.200 mmol) was added dropwise at 0 °C. After 30 min, saturated aqueous $NaHCO_3$ (5 mL) was added, followed by phase separation and extraction of the aqueous phase with DCM (3 × 10 mL). The unified organic phases were washed with brine (1 × 20 mL), dried over Na_2SO_4, filtered and the solvent was removed. After column chromatography (silica, petroleum ether/EtOAc, 20:1 nach 10:1) product **26** (35 mg, 67.191 µmol, 67%) was obtained as yellowish oil. – R_f (petroleum ether/EtOAc, 20:1) = 0.23. – $[\alpha]_D^{23}$ = +1.1 (*c* = 1.4, $CHCl_3$). – ^{1}H NMR (400 MHz, $CDCl_3$): δ = 9.75 (d, *J* = 6.1 Hz, 1H, C*H*O), 5.72 (m, 1H, $C_qCHCH_2CH_2$), 2.82 (dd, *J* = 5.4, 11.9 Hz, 1H, C*H*CHO), 2.50 (m, 2H, $O{=}C_qCH_2CH_2$), 2.49 (m, 2H, $O{=}C_qCH_2CH_2$), 2.14 (s, 3H, $CH_3C_q{=}O$), 2.04 (m, 1H, $(CH_3)_2CHCH$), 1.85 (s, 3H, $CH_3C_qCHCH_2CH_2$), 1.84 (m, 1H, $TBSOC_qCHH$), 1.62 (m, 1H, $TBSOC_qCHH$), 1.53 (m, 1H, $TBSOC_qCH_2CHH$), 1.52 (m, 1H, $(CH_3)_2CH$), 1.36 (m, 1H, $TBSOC_qCH_2CHH$), 1.34 (s, 3H, CH_3C_qOTBS), 0.91 (d, *J* = 6.9 Hz, 3H, CH_3CHCH_3), 0.88 (s, 9H, $SiC_q(CH_3)_3$), 0.77 (d, *J* = 6.8 Hz, 3H, CH_3CHCH_3), 0.15 (s, 9H, $Si(CH_3)_3$), 0.12 (s, 3H, CH_3SiCH_3), 0.10 (s, 3H, CH_3SiCH_3). – ^{13}C NMR (100 MHz, $CDCl_3$): δ = 207.4 (1C, $C_q{=}O$), 205.9 (1C, *C*HO), 136.8 (1C, $CH_3C_qCHCH_2CH_2$), 118.3 (1C, $CH_3C_qCHCH_2CH_2$), 93.0 (1C, $TMSOC_qC_qC_q$), 89.8 (1C, $TMSOC_qC_qC_q$), 77.8 (1C, $TBSOC_q$), 77.1 (1C, C_qOTMS), 59.1 (1C, *C*HCHO), 43.0 (1C, $O{=}C_qCH_2CH_2$), 40.4 (1C, $(CH_3)_2CHCH$), 37.2 (1C, $TBSOC_qCH_2$), 29.9 (1C, $CH_3C_q{=}O$), 29.0 (1C, $(CH_3)_2CH$), 26.1 (3C, $SiC_q(CH_3)_3$), 25.1 (1C, $O{=}C_qCH_2CH_2$), 24.5 (1C, CH_3C_qOTBS), 22.7 (1C, $CH_3C_qCHCH_2CH_2$), 21.2 (1C, CH_3CHCH_3), 18.6 (1C, SiC_q), 18.0 (1C, $TBSOC_qCH_2CH_2$), 15.5 (1C, CH_3CHCH_3), 2.4 (3C, $Si(CH_3)_3$), -2.0 (1C, CH_3SiCH_3), -2.2 (1C, CH_3SiCH_3). – HRESI(+)MS: calcd for $C_{29}H_{52}NaO_4Si_2$ $[M+Na]^+$: 543.32963, found 543.32962.

(1*R*,2*R*,3*R*,6*R*)-3-((*tert*-Butyldimethylsilyl)oxy)-2-((*Z*)-7-hydroxy-3-methyloct-3-en-1-yn-1-yl)-6-isopropyl-3-methyl-2-((trimethylsilyl)oxy)cyclohexane-1-carbaldehyde (27)

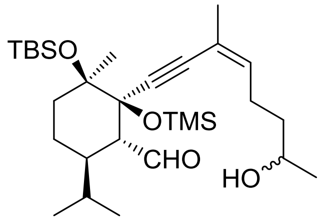

27

Under argon zinc powder (73 mg, 1.109 mmol) was added dropwise to a solution of $TiCl_4$ (1.0 M in DCM, 550 µL, 0.550 mmol) in THF (12 mL) at 0 °C. After 10 min at rt pyridine (46 µL, 0.570 mmol) was added. Over 2.5 h, a solution of ketoaldehyde **26** (15 mg, 28.796 µmol) in THF (2.5 mL) was added dropwise, followed by stirring at 60 °C for 12 h. Aqueous K_2CO_3 (15 mL, 10%) was added and it was extracted with EtOAc (4×20 mL). The unified organic phases were dried over $MgSO_4$ and the solvent was removed. After column chromatography (silica, petroleum ether/EtOAc, 10:1) product **27** (6.1 mg, 11.665 µmol, 40%, 55% brsm) was obtained as colorless oil as mixture of diastereomers (**27a**/**27b**, 1:1). – R_f (**27a**, **27b**, petroleum ether/EtOAc, 5:1) = 0.20. – ^{1}H NMR (600 MHz, $CDCl_3$, **27a**): δ = 9.77

(d, J = 5.9 Hz, 1H, C*H*O), 5.75 (m, 1H, $C_qCHCH_2CH_2$), 3.81 (m, 1H, C*H*OH), 2.84 (ddd, J = 2.3, 5.9, 12.0 Hz, 1H, C*H*CHO), 2.35 (m, 1H, $OHCHCH_2CH_2$), 2.06 (dddd, J = 3.6, 3.6, 12.5, 12.5 Hz, 1H, $(CH_3)_2CHCH$), 1.87 (d, J = 1.0 Hz, 3H, $CH_3C_qCHCH_2CH_2$), 1.84 (dd, J = 4.2, 13.6 Hz, 1H, $TBSOC_qCHH$), 1.62 (m, 1H, $TBSOC_qCHH$), 1.55 (m, 1H, $(CH_3)_2CH$), 1.54 (m, 1H, $OHCHCH_2CH_2$), 1.51 (m, 1H, $TBSOC_qCH_2CHH$), 1.36 (m, 1H, $TBSOC_qCH_2CHH$), 1.35 (s, 3H, CH_3C_qOTBS), 1.21 (s, 3H, $OHCHCH_3$), 0.91 (d, J = 6.9 Hz, 3H, CH_3CHCH_3), 0.89 (s, 9H, $SiC_q(CH_3)_3$), 0.77 (d, J = 6.8 Hz, 3H, CH_3CHCH_3), 0.15 (s, 9H, $Si(CH_3)_3$), 0.13 (s, 3H, CH_3SiCH_3), 0.10 (s, 3H, CH_3SiCH_3). – ^{13}C NMR (150 MHz, $CDCl_3$, **27a**): δ = 206.2 (1C, *C*HO), 138.4 (1C, $CH_3C_qCHCH_2CH_2$), 117.4 (1C, $CH_3C_qCHCH_2CH_2$), 92.5 (1C, $TMSOC_qC_qC_q$), 90.1 (1C, $TMSOC_qC_qC_q$), 77.8 (1C, $TBSOC_q$), 77.2 (1C, C_qOTMS), 67.6 (1C, *C*HOH), 59.1 (1C, *C*HCHO), 40.3 (1C, $(CH_3)_2CHCH$), 38.6 (1C, $HOCHCH_2$), 37.2 (1C, $TBSOC_qCH_2$), 28.9 (1C, $(CH_3)_2CH$), 27.4 (1C, $HOCHCH_2CH_2$), 26.0 (3C, $SiC_q(CH_3)_3$), 24.5 (1C, CH_3C_qOTBS), 23.5 (1C, CH_3CHOH), 22.8 (1C, $CH_3C_qCHCH_2CH_2$), 21.2 (1C, CH_3CHCH_3), 18.6 (1C, SiC_q), 17.9 (1C, $TBSOC_qCH_2CH_2$), 15.5 (1C, CH_3CHCH_3), 2.4 (3C, $Si(CH_3)_3$), -2.0 (1C, CH_3SiCH_3), -2.2 (1C, CH_3SiCH_3). – ^{1}H NMR (600 MHz, $CDCl_3$, **27b**): δ = 9.77 (d, J = 5.9 Hz, 1H, C*H*O), 5.75 (m, 1H, $C_qCHCH_2CH_2$), 3.81 (m, 1H, C*H*OH), 2.84 (ddd, J = 2.3, 5.9, 12.0 Hz, 1H, C*H*CHO), 2.35 (m, 1H, $OHCHCH_2CH_2$), 2.06 (dddd, J = 3.6, 3.6, 12.5, 12.5 Hz, 1H, $(CH_3)_2CHCH$), 1.87 (d, J = 1.0 Hz, 3H, $CH_3C_qCHCH_2CH_2$), 1.84 (dd, J = 4.2, 13.6 Hz, 1H, $TBSOC_qCHH$), 1.62 (m, 1H, $TBSOC_qCHH$), 1.55 (m, 1H, $(CH_3)_2CH$), 1.54 (m, 1H, $OHCHCH_2CH_2$), 1.51 (m, 1H, $TBSOC_qCH_2CHH$), 1.36 (m, 1H, $TBSOC_qCH_2CHH$), 1.35 (s, 3H, CH_3C_qOTBS), 1.20 (s, 3H, $OHCHCH_3$), 0.91 (d, J = 6.9 Hz, 3H, CH_3CHCH_3), 0.89 (s, 9H, $SiC_q(CH_3)_3$), 0.77 (d, J = 6.8 Hz, 3H, CH_3CHCH_3), 0.15 (s, 9H, $Si(CH_3)_3$), 0.13 (s, 3H, CH_3SiCH_3), 0.10 (s, 3H, CH_3SiCH_3). – ^{13}C NMR (150 MHz, $CDCl_3$, **27b**): δ = 206.2 (1C, *C*HO), 138.4 (1C, $CH_3C_qCHCH_2CH_2$), 117.4 (1C, $CH_3C_qCHCH_2CH_2$), 92.5 (1C, $TMSOC_qC_qC_q$), 90.1 (1C, $TMSOC_qC_qC_q$), 77.8 (1C, $TBSOC_q$), 77.2 (1C, C_qOTMS), 67.6 (1C, *C*HOH), 59.1 (1C, *C*HCHO), 40.3 (1C, $(CH_3)_2CHCH$), 36.6 (1C, $HOCHCH_2$), 37.2 (1C, $TBSOC_qCH_2$), 28.9 (1C, $(CH_3)_2CH$), 27.4 (1C, $HOCHCH_2CH_2$), 26.0 (3C, $SiC_q(CH_3)_3$), 24.5 (1C, CH_3C_qOTBS), 23.5 (1C, CH_3CHOH), 22.8 (1C, $CH_3C_qCHCH_2CH_2$), 21.2 (1C, CH_3CHCH_3), 18.6 (1C, SiC_q), 17.9 (1C, $TBSOC_qCH_2CH_2$), 15.5 (1C, CH_3CHCH_3), 2.4 (3C, $Si(CH_3)_3$), -2.0 (1C, CH_3SiCH_3), -2.2 (1C, CH_3SiCH_3). – HRESI(+)MS: calcd for $C_{29}H_{54}NaO_4Si_2$ $[M+Na]^+$: 545.34528, found 545.34568.

Ethyl (1*R*,2*S*,3*R*,6*R*)-2-cyano-2-hydroxy-6-isopropyl-3-methoxy-3-methylcyclohexane-1-carboxylate (29) and ethyl (1*R*,2*R*,3*R*,6*R*)-2-cyano-2-hydroxy-6-isopropyl-3-methoxy-3-methylcyclohexane-1-carboxylate (30)

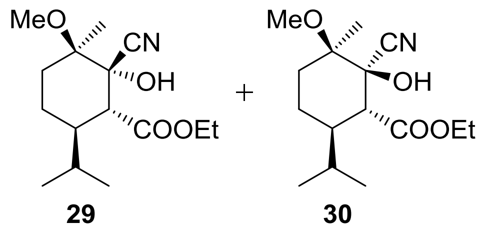

Under argon at −78 °C NaHMDS (1.0 M in THF, 7.50 mL, 7.500 mmol) was added to a solution of ketone **28** (1.383 g, 7.505 mmol) in THF (30 mL). After warming to 0 °C over 30 min CNCOOEt (2.2 mL, 22.515 mmol) was added dropwise at −78 °C, followed by 30 min of stirring. Saturated aqueous NH_4Cl (30 mL) and EtOAc (40 mL) were added. At rt the phases were separated and the aqueous phase was extracted with EtOAc (3 × 30 mL). The unified organic phases were dried over Na_2SO_4, filtered and the solvent was removed. After column chromatography (silica, petroleum ether/EtOAc, 10:1 to 5:1) diastereomer **29** (1.040 g, 3.670 mmol, 49%) was obtained as colorless oil and **30** (610 mg, 2.153 g, 29%) als crystalline colorless solid. – R_f (**29**, petroleum ether/EtOAc, 5:1) = 0.62. –

$[\alpha]_D^{22}$ (**29**) = −20.1 (*c* = 0.7, MeOH). – ^{1}H NMR (400 MHz, $CDCl_3$, **29**): δ = 5.25 (s, 1H, O*H*), 4.32 (qd, *J* = 7.2, 10.8 Hz, 1H, C*H*HCH_3), 4.25 (qd, *J* = 7.1, 10.8 Hz, 1H, CH*H*CH_3), 3.24 (s, 3H, OCH_3), 3.16 (d, *J* = 12.1 Hz, 1H, C*H*C_qCOOEt), 1.84 (m, 1H, $(CH_3)_2$CHC*H*), 1.83 (m, 1H, MeOC_qC*H*H), 1.60 (m, 1H, MeOC_qCH*H*), 1.50 (m, 1H, $(CH_3)_2$C*H*), 1.38 (m, 1H, MeO$C_q$$CH_2$C*H*H), 1.36 (s, 3H, C$H_3$$C_q$OMe), 1.33 (t, *J* = 7.2 Hz, 3H, C$H_3$$CH_2$), 1.23 (m, 1H, MeO$C_q$$CH_2$CH*H*), 0.92 (d, *J* = 6.9 Hz, 3H, CH_3CHCH_3), 0.86 (d, *J* = 6.9 Hz, 3H, CH_3CHCH_3). – ^{13}C NMR (100 MHz, $CDCl_3$, **29**): δ = 176.4 (1C, *C*$_q$OOEt), 120.1 (1C, *C*N), 76.1 (1C, *C*$_q$CN), 75.2 (1C, MeO*C*$_q$), 61.7 (1C, *C*H_2CH_3), 49.2 (1C, *C*HC_qOOEt), 39.3 (1C, $(CH_3)_2$CH*C*H), 29.1 (1C, $(CH_3)_2$*C*H), 26.7 (1C, MeOC_q*C*H_2), 21.1 (1C, *C*H_3CHCH_3), 18.8 (1C, *C*H_3C_qOMe), 18.0 (1C, MeO$C_q$$CH_2$*C*$H_2$), 15.5 (1C, CH_3CH*C*H_3), 14.1 (1C, *C*H_3CH_2). – IR (ATR, **29**): $\tilde{\nu}$ = 3421 (br, w), 2958 (m), 1751 (w), 1700 (s), 1464 (m), 1374 (m), 1333 (m), 1189 (s),1110 (m), 1091 (s), 1017 (s), 870 (m) cm^{-1}. – UV/Vis (CH_2Cl_2, **29**): 229 (2.16), 309 (1.88) nm. – HRESI(+)MS (**29**): calcd for $C_{15}H_{25}NNaO_4$ $[M+Na]^+$: 306.16758, found 306.16774. – R_f (**30**, petroleum ether/EtOAc, 5:1) = 0.22. – mp.161-162 °C. – $[\alpha]_D^{22}$ (**30**) = −3.1 (*c* = 1.1, MeOH). – ^{1}H NMR (400 MHz, $CDCl_3$, **30**): δ = 4.29 (qd, *J* = 7.2, 11.0 Hz, 1H, C*H*HCH_3), 4.21 (qd, *J* = 7.3, 10.8 Hz, 1H, CH*H*CH_3), 3.25 (s, 3H, OCH_3), 3.00 (s, 1H, O*H*), 2.95 (d, *J* = 12.0 Hz, 1H, C*H*C_qCOOEt), 2.07 (ddd, *J* = 3.3, 3.3, 15.4 Hz, 1H, MeOC_qC*H*H), 1.92 (m, 1H, $(CH_3)_2$CHC*H*), 1.66 (m, 1H, $(CH_3)_2$C*H*), 1.64 (m, 1H, MeOC_qCH*H*), 1.46 (ddd, *J* = 3.3, 6.4, 12.4 Hz, 1H, MeO$C_q$$CH_2$C*H*H), 1.43 (s, 3H, C$H_3$$C_q$OMe), 1.30 (t, *J* = 7.1 Hz, 3H, C$H_3$$CH_2$), 1.16 (ddd, *J* = 3.9, 13.4, 26.3 Hz, 1H, MeO$C_q$$CH_2$CH*H*), 0.94 (d, *J* = 6.9 Hz, 3H, CH_3CHCH_3), 0.83 (d, *J* = 6.9 Hz, 3H, CH_3CHCH_3). – ^{13}C NMR (100 MHz, $CDCl_3$, **30**): δ = 171.2 (1C, *C*$_q$OOEt), 118.0 (1C, *C*N), 77.7 (1C, *C*$_q$CN), 77.6 (1C, MeO*C*$_q$), 60.9 (1C, *C*H_2CH_3), 53.1 (1C, *C*HC_qOOEt), 42.8 (1C, $(CH_3)_2$CH*C*H), 29.7 (1C, MeOC_q*C*H_2), 28.9 (1C, $(CH_3)_2$*C*H), 21.1 (1C, *C*H_3CHCH_3), 18.4 (1C, *C*H_3C_qOMe), 18.3 (1C, MeO$C_q$$CH_2$*C*$H_2$), 15.6 (1C, CH_3CH*C*H_3), 14.1 (1C, *C*H_3CH_2). – IR (ATR, **30**): $\tilde{\nu}$ = 3421 (w), 2958 (m), 1751 (w), 1700 (s), 1464 (w), 1374 (m), 1333 (m), 1189 (s),1110 (m), 1091 (m), 1017 (s), 870 (w) cm^{-1}. – UV/Vis (CH_2Cl_2, **30**): 230 (2.39) nm. – HRESI(+)MS (**30**): calcd for $C_{15}H_{25}NNaO_4$ $[M+Na]^+$: 306.16758, found 306.16759.

(2*R*,5*S*)-5-Isopropyl-2-methoxy-2-methylcyclohexan-1-one (28)

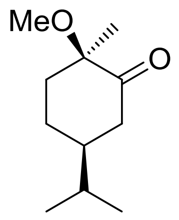

28

A suspension of PtO_2 (7 mg, 0.031 mmol) in a solution of diol **20** (520 mg, 3.054 mmol) in MeOH (20 mL) was stirred under an H_2 atmosphere (balloon) for 3 h. It was filtered over Celite and the solvent was removed. The crude product was dissolved in EtOAc, followed by addition of IBX (1.112 g, 3.970 mmol) and refluxing (5 h). After cooling to rt the suspension was filtered of a pad of silica and the solvent was removed. Under argon the crude product was dissolved in CH_3CN (20 mL) and MeI (6.4 mL, 102.795 mmol) and Ag_2O (6.4 mL, 11.014 mmol) were added, followed stirring at 40 °C for 17 h. It was filtered over Celite and the solvent was removed. After column chromatography (silica, petroleum ether/EtOAc, 10:1) product **28** (540 mg, 2.930 mmol, 96%) was obtained as colorless oil. – R_f (petroleum ether/EtOAc, 5:1) = 0.65. – $[\alpha]_D^{20}$ = −21.2 (*c* = 2.7, $CHCl_3$). – ^{1}H NMR (400 MHz, $CDCl_3$): δ = 3.14 (s, 3H, OCH_3), 2.51 (dd, *J* = 12.6, 12.6 Hz, 1H, C*H*HC_q=O), 2.25 (ddd, *J* = 2.1, 3.5, 12.1 Hz, 1H, CH*H*C_q=O), 2.13 (ddd, *J* = 2.7, 3.7, 14.3 Hz, 1H, MeOC_qC*H*H), 1.75 (m, 1H, MeO$C_q$$CH_2$C*H*H), 1.57 (m, 1H, $(CH_3)_2$C*H*), 1.53 (m, 1H, MeO$C_q$$CH_2$CH*H*), 1.50 (m, 1H, $(CH_3)_2$CHC*H*), 1.43 (ddd, *J* = 4.1, 13.4, 14.2 Hz, 1H, MeOC_qCH*H*), 1.19 (s, 3H, C$H_3$$C_q$$C_q$=O), 0.91 (d,

J = 6.7 Hz, 3H, CH_3CHCH$_3$), 0.90 (d, J = 6.7 Hz, 3H, CH$_3$CHCH_3). – ^{13}C NMR (100 MHz, CDCl$_3$): δ = 213.6 (1C, C_q=O), 79.9 (1C, MeOC_q), 51.0 (1C, CH$_3$OC$_q$), 46.9 (1C, (CH$_3$)$_2$CHCH), 42.1 (1C, CH$_2$C$_q$=O), 39.6 (1C, MeOC$_q$$CH_2$), 32.8 (1C, (CH$_3$)$_2$$C$H), 23.8 (1C, MeOC$_qCH_2$$CH_2$), 19.7 (1C, CH$_3$CHCH$_3$), 19.4 (1C, CH$_3$CHCH$_3$), 17.6 (1C, CH$_3C_q$OMe). – IR (ATR): $\tilde{\nu}$ = 2957 (m), 2874 (w), 1716 (s), 1463 (w), 1371 (w), 1175 (w), 1083 (s), 1059 (m), 696 (w) cm^{-1}. – UV/Vis (CH$_2$Cl$_2$): λ_{max} (lg ε) = 229 (1.97), 309 (1.70) nm. – MS (GC-MS, EI): m/z (%) = 184 (22) [M]$^+$, 140 (36), 113 (64), 85 (100), 81 (22), 72 (41), 55 (24). – HREIMS: calcd for $C_{11}H_{20}O_2$ [M]$^{\cdot+}$: 184.14578, found 184.14508.

Ethyl (1*R*,2*R*,3*R*,6*R*)-3-((*tert*-butyldimethylsilyl)oxy)-2-hydroxy-6-isopropyl-3-methyl-2-((*E*)-3-methyl-6-(2-methyl-1,3-dioxolan-2-yl)hex-3-en-1-yn-1-yl)cyclohexane-1-carboxylate (31)

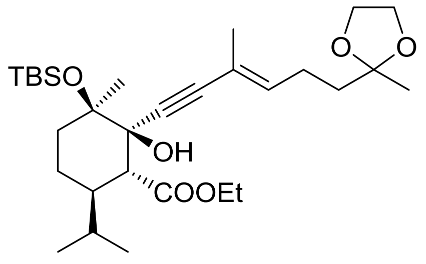

31

Under argon at −25 °C, n-BuLi (1.6 M in hexane, 347 µL, 0.555 mmol) was added to a solution of (E)-2-methyl-2-(4-methylhex-3-en-5-yn-1-yl)-1,3-dioxolane (100 mg, 0.555 mmol) in THF (3 mL). After 10 min, it was cooled to −78 °C and a solution of ketoester **23** (218 mg, 0.611 mmol) in THF (4.5 mL) was added dropwise over a period of 2 h. H$_2$O (10 mL) and EtOAc (12 mL) were added, the phases separated and the aqueous phase extracted with EtOAc (3 × 10 mL). The unified organic phases were with washed with brine (1 × 20 mL), dried over Na$_2$SO$_4$, filtered, and the solvent was removed. After column chromatography (silica, petroleum ether/EtOAc, 10:1) product **31** (193 mg, 0.360 mmol, 65%, 74% based on re-isolated alkyne) was obtained as colorless oil. – R_f (petroleum ether/EtOAc, 5:1) = 0.40. – $[\alpha]_D^{25}$ = +21.9 (c = 2.9, CHCl$_3$). – ^{1}H NMR (400 MHz, CDCl$_3$): δ = 5.86 (qt, J = 1.4, 7.4 Hz, 1H, CH$_3$C$_q$CHCH$_2$CH$_2$), 4.07 (qd, J = 7.1, 10.8 Hz, 1H, CHHCH$_3$), 4.20 (qd, J = 7.1, 10.8 Hz, 1H, CHHCH$_3$), 3.94 (m, 4H, OCH_2CH_2O), 2.87 (d, J = 11.9 Hz, 1H, CHC$_q$COOEt), 2.54 (s, 1H, OH), 2.18 (m, 2H, CH$_3$C$_q$CHCH_2CH$_2$), 1.93 (m, 1H, (CH$_3$)$_2$CHCH), 1.82 (m, 1H, TBSOC$_q$CHH), 1.80 (m, 3H, CH_3C$_q$CHCH$_2$CH$_2$), 1.70 (m, 2H, CH$_3$C$_q$CHCH$_2$CH_2), 1.69 (m, 1H, TBSOC$_q$CHH), 1.69 (m, 1H, (CH$_3$)$_2$CH), 1.45 (s, 3H, CH_3C$_q$OTBS), 1.38 (m, 2H, TBSOC$_q$CH$_2$CH_2), 1.32 (s, 3H, CH_3C$_q$COCH$_2$CH$_2$O), 1.26 (t, J = 7.1 Hz, 3H, CH_3CH$_2$), 0.93 (s, 9H, SiC$_q$(CH_3)$_3$), 0.91 (d, J = 7.0 Hz, 3H, CH_3CHCH$_3$), 0.80 (d, J = 6.9 Hz, 3H, CH$_3$CHCH_3), 0.16 (s, 6H, CH_3SiCH_3). – ^{13}C NMR (100 MHz, CDCl$_3$): δ = 172.3 (1C, C_qOOEt), 137.6 (1C, CH$_3$C$_q$$CHCH_2CH_2$), 117.6 (1C, CH$_3$$C_q$CHCH$_2CH_2$), 109.6 (1C, CH$_3$$C_q$COCH$_2CH_2$O), 90.8 (1C, HOC$_q$$C_qC_q$), 83.9 (1C, HOC$_qC_q$$C_q$), 78.5 (1C, C_qOH), 76.5 (1C, TBSOC_q), 60.0 (1C, CH$_2$CH$_3$), 54.4 (1C, CHC$_q$OOEt), 64.7 (2C, OCH$_2$$CH_2$O), 42.3 (1C, (CH$_3$)$_2CHC$H), 38.1 (1C, CH$_3C_q$CHCH$_2$$CH_2$), 35.9 (1C, TBSOC$_q$$CH_2$), 28.8 (1C, (CH$_3$)$_2$$C$H), 26.1 (3C, SiC$_q$($CH_3$)$_3$), 24.4 (1C, CH$_3C_q$OTBS), 23.1 (1C, CH$_3C_q$CHCH$_2CH_2$), 23.9 (1C, CH$_3C_q$COCH$_2CH_2$O), 21.4 (1C, CH$_3$CHCH$_3$), 18.5 (1C, SiC_q), 18.3 (1C, TBSOC$_q$CH$_2$$CH_2$), 17.0 (1C, CH$_3C_q$CHCH$_2CH_2$), 15.8 (1C, CH$_3$CHCH$_3$), 14.3 (1C, CH$_3CH_2$), -1.9 (1C, CH$_3$SiCH$_3$), -2.5 (1C, CH$_3$SiCH$_3$). – IR (ATR): $\tilde{\nu}$ = 3456 (br, w), 2954 (s), 2883 (m), 1734 (m), 1467 (w), 1372 (m), 1250 (m), 1146 (m), 1094 (m), 1048 (s), 833 (s), 775 (m) cm^{-1}. – UV/Vis (CH$_2$Cl$_2$): 230 (3.45) nm. – HRESI(+)MS: calcd for $C_{30}H_{52}NaO_6Si$ [M+Na]$^+$: 559.34254, found 559.34246.

Allene 32

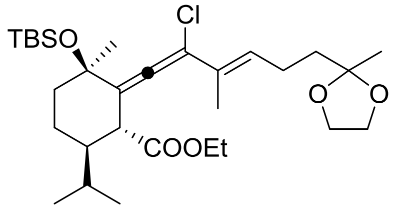

32

Under argon at 0 °C, thionyl chloride (13.5 µL, 0.186 mmol) was added to a solution of **31** (10 mg, 18.628 µmol) in pyridine (1 mL). Saturated aqueous $NaHCO_3$ (10 mL) and EtOAc (10 mL) were added, the phases separated and the aqueous phase extracted with EtOAc (3 × 10 mL). The unified organic phases were washed with brine (1 × 10 mL), dried over Na_2SO_4, filtered, and the solvent was removed. After column chromatography (silica, petroleum ether/EtOAc, 20:1) product **32** (8 mg, 15.420 µmol, 83%) was obtained as colorless oil. – R_f (petroleum ether/EtOAc, 5:1) = 0.61. – $[\alpha]_D^{22}$ = –6.0 (c = 0.67, $CHCl_3$). – ^{1}H NMR (400 MHz, $CDCl_3$): δ = 5.92 (qd, J = 1.1, 7.4 Hz, 1H, $C_qCHCH_2CH_2$), 4.08 (m, 2H, CH_2CH_3), 3.95 (m, 4H, OCH_2CH_2O), 3.48 (d, J = 11.7 Hz, 1H, CHC_qCOOEt), 2.25 (m, 2H, $CH_3C_qCHCH_2CH_2$), 1.93 (td, J = 3.2, 13.2 Hz, 1H, $TBSOC_qCHH$), 1.79 (m, 1H, $(CH_3)_2CHCH$), 1.75 (m, 1H, $(CH_3)_2CH$), 1.75 (m, 2H, $CH_3C_qCHCH_2CH_2$), 1.71 (dd, J = 0.8, 1.7 Hz, 3H, $CH_3C_qCHCH_2CH_2$), 1.65 (dddd, J = 3.6, 13.0, 13.0, 13.1 Hz, 1H, $TBSOC_qCH_2CHH$), 1.49 (dddd, J = 3.5, 3.5, 3.5, 13.2 Hz, 1H, $TBSOC_qCH_2CHH$), 1.37 (m, 1H, $TBSOC_qCHH$), 1.34 (s, 3H, $CH_3C_qCOCH_2CH_2O$), 1.32 (s, 3H, CH_3C_qOTBS), 1.21 (t, J = 7.1 Hz, 3H, CH_3CH_2), 0.93 (d, J = 6.9 Hz, 3H, CH_3CHCH_3), 0.92 (s, 9H, $SiC_q(CH_3)_3$), 0.82 (d, J = 6.9 Hz, 3H, CH_3CHCH_3), 0.20 (s, 3H, CH_3SiCH_3), 0.11 (s, 3H, CH_3SiCH_3). – ^{13}C NMR (100 MHz, $CDCl_3$): δ = 194.3 (1C, $C_qC_qC_qCl$), 172.4 (1C, C_qOOEt), 129.3 (1C, $CH_3C_qCHCH_2CH_2$), 127.8 (1C, $CH_3C_qCHCH_2CH_2$), 117.5 (1C, $C_qC_qC_qCl$), 110.9 (1C, $C_qC_qC_qCl$), 109.7 (1C, $CH_3C_qCOCH_2CH_2O$), 72.6 (1C, $TBSOC_q$), 64.7 (1C, OCH_2CH_2O), 64.7 (1C, OCH_2CH_2O), 60.4 (1C, CH_2CH_3), 48.7 (1C, CHC_qOOEt), 44.9 (1C, $(CH_3)_2CHCH$), 42.0 (1C, $TBSOC_qCH_2$), 38.5 (1C, $CH_3C_qCHCH_2CH_2$), 29.3 (1C, $(CH_3)_2CH$), 28.8 (1C, CH_3C_qOTBS), 26.0 (3C, $SiC_q(CH_3)_3$), 23.9 (1C, $CH_3C_qCOCH_2CH_2O$), 23.4 (1C, $CH_3C_qCHCH_2CH_2$), 21.3 (1C, CH_3CHCH_3), 18.6 (1C, $TBSOC_qCH_2CH_2$), 18.3 (1C, SiC_q), 15.9 (1C, CH_3CHCH_3), 14.6 (1C, $CH_3C_qCHCH_2CH_2$), 14.0 (1C, CH_3CH_2), -2.0 (1C, CH_3SiCH_3), -3.3 (1C, CH_3SiCH_3). – IR (ATR): $\tilde{\nu}$ = 2956 (m), 1736 (m), 1467 (w), 1374 (w), 1252 (m), 1016 (m), 983 (m), 834 (s), 775 (s), 690 (w) cm^{-1}. – UV/Vis (CH_2Cl_2): 233 (3.91), 275 (3.27), 320 (2.89) nm. – HRESI(+)MS: calcd for $C_{30}H_{51}ClNaO_5Si$ $[M+Na]^+$: 577.30865, found 577.30877.

Allene 34

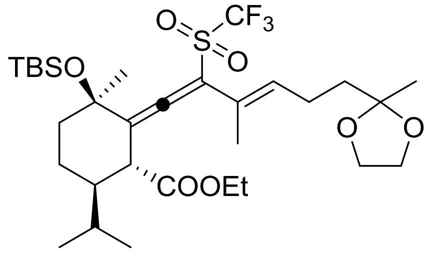

34

Under argon at 0 °C, Tf_2O (14 µL, 81.964 mmol) was added to a solution of **31** (22 mg, 40.982 µmol) in pyridine (1.5 mL). It was warmed to 70 °C and stirred for 20 min. Saturated aqueous $NaHCO_3$ (5 mL) and EtOAc (5 mL) were added, the phases were separated and the aqueous phase was extracted with EtOAc (3 × 5 mL). The unified organic phases were washed with brine (1 × 10 mL), dried over Na_2SO_4, filtered and the solvent removed. After column chromatography (silica, petroleum ether/EtOAc, 20:1) product **34** (17 mg, 26.038 µmol, 64%) was obtained as colorless oil. – R_f (petroleum ether/EtOAc, 10:1) = 0.54. – $[\alpha]_D^{23}$ = –2.8 (c = 1.7, $CHCl_3$). – ^{1}H NMR (400 MHz, $CDCl_3$): δ = 6.06 (t, J = 7.3 Hz, 1H, $C_qCHCH_2CH_2$), 4.11 (m, 2H, CH_2CH_3), 3.95 (m, 4H, OCH_2CH_2O), 3.54 (d, J = 11.2 Hz, 1H, CHC_qCOOEt), 2.25 (m, 2H, $CH_3C_qCHCH_2CH_2$), 1.97 (td, J = 2.8, 12.9 Hz, 1H, $TBSOC_qCHH$), 1.87 (d, J = 0.9 Hz, 3H, $CH_3C_qCHCH_2CH_2$),

1.78 (m, 1H, $(CH_3)_2CHCH$), 1.74 (m, 2H, $CH_3C_qCHCH_2CH_2$), 1.71 (m, 1H, $(CH_3)_2CH$), 1.71 (m, 1H, $TBSOC_qCH_2CHH$), 1.53 (ddd, J = 2.9, 5.9, 9.7 Hz, 1H, $TBSOC_qCH_2CHH$), 1.43 (m, 1H, $TBSOC_qCHH$), 1.38 (s, 3H, CH_3C_qOTBS), 1.33 (s, 3H, $CH_3C_qCOCH_2CH_2O$), 1.24 (t, J = 7.1 Hz, 3H, CH_3CH_2), 0.94 (d, J = 6.9 Hz, 3H, CH_3CHCH_3), 0.91 (s, 9H, $SiC_q(CH_3)_3$), 0.86 (d, J = 6.9 Hz, 3H, CH_3CHCH_3), 0.18 (s, 3H, CH_3SiCH_3), 0.13 (s, 3H, CH_3SiCH_3). – ^{13}C NMR (100 MHz, $CDCl_3$): δ = 203.7 (1C, $C_qC_qC_qTf$), 171.5 (1C, C_qOOEt), 134.5 (1C, $CH_3C_qCHCH_2CH_2$), 123.6 (1C, $CH_3C_qCHCH_2CH_2$), 120.1 (q, 1C, $J(^{19}F,^{13}C)$ = 328.8 Hz, CF_3), 119.5 (1C, $C_qC_qC_qTf$), 113.5 (1C, $C_qC_qC_qTf$), 109.6 (1C, $CH_3C_qCOCH_2CH_2O$), 72.6 (1C, $TBSOC_q$), 64.7 (2C, OCH_2CH_2O), 60.7 (1C, CH_2CH_3), 48.1 (1C, CHC_qOOEt), 46.2 (1C, $(CH_3)_2CHCH$), 42.3 (1C, $TBSOC_qCH_2$), 38.1 (1C, $CH_3C_qCHCH_2CH_2$), 29.3 (1C, $(CH_3)_2CH$), 28.2 (1C, CH_3C_qOTBS), 25.9 (3C, $SiC_q(CH_3)_3$), 23.9 (1C, $CH_3C_qCOCH_2CH_2O$), 23.6 (1C, $CH_3C_qCHCH_2CH_2$), 21.2 (1C, CH_3CHCH_3), 18.7 (1C, $TBSOC_qCH_2CH_2$), 18.3 (1C, SiC_q), 16.4 (1C, $CH_3C_qCHCH_2CH_2$), 15.6 (1C, CH_3CHCH_3), 14.0 (1C, CH_3CH_2), -2.3 (1C, CH_3SiCH_3), -3.5 (1C, CH_3SiCH_3). – IR (ATR): $\tilde{\nu}$ = 2933 (w), 1737 (m), 1360 (m),1205 (s), 1121 (s),1020 (m), 836 (m), 778 (m) cm^{-1}. – UV/Vis (CH_2Cl_2): 227 (3.91), 262 (3.92), 356 (2.42) nm. – HRESI(+)MS: calcd for $C_{31}H_{51}F_3NaO_7SSi$ $[M+Na]^+$: 675.29691, found 675.29721.

X-ray analysis of 30

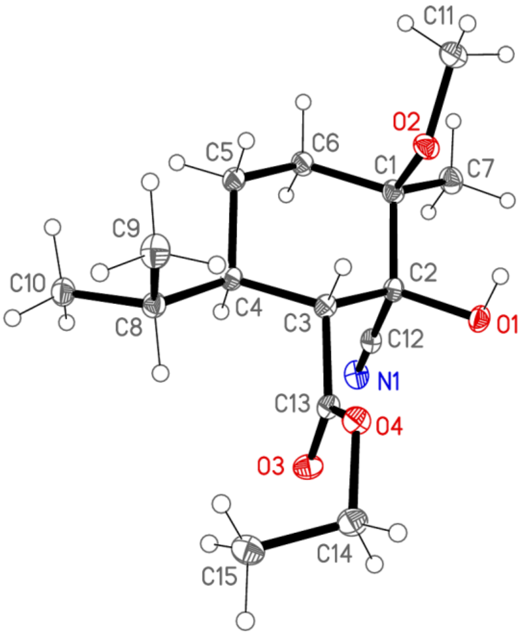

Table 1. Crystal data and structure refinement.

Identification code	frett	
Empirical formula	$C_{15}H_{25}NO_4$	
Formula weight	283.36	
Temperature	101(2) K	
Wavelength	1.54184 Å	
Crystal system	Monoclinic	
Space group	$P2_1$	
Unit cell dimensions	*a* = 7.59870(16) Å	α = 90°
	b = 11.4650(3) Å	β = 108.479(3)°
	c = 9.1080(2) Å	γ = 90°
Volume	752.57(3) $Å^3$	
Z	2	
Density (calculated)	1.250 Mg/m^3	
Absorption coefficient	0.732 mm^{-1}	
F(000)	308	
Crystal size	0.20 x 0.20 x 0.05 mm^3	
Theta range for data collection	5.12 to 76.15°	
Index ranges	-9<=h<=9, -14<=k<=12, -11<=l<=11	
Reflections collected	15535	
Independent reflections	2914 [R(int) = 0.0302]	
Completeness to theta = 75.00°	99.9 %	
Absorption correction	Semi-empirical from equivalents	
Max. and min. transmission	1.00000 and 0.77877	
Refinement method	Full-matrix least-squares on F^2	
Data / restraints / parameters	2914 / 1 / 190	
Goodness-of-fit on F^2	1.040	
Final R indices [I>2sigma(I)]	R1 = 0.0284, wR2 = 0.0752	
R indices (all data)	R1 = 0.0286, wR2 = 0.0755	
Absolute structure parameter	0.00(13)	
Largest diff. peak and hole	0.270 and -0.151 e.$Å^{-3}$	

Table 2. Atomic coordinates (x 10^4) and equivalent isotropic displacement parameters ($Å^2$x 10^3). U(eq) is defined as one third of the trace of the orthogonalized U_{ij} tensor.

	x	y	z	U(eq)
C(1)	2734.5(15)	1874.0(11)	739.7(13)	22.2(2)
C(2)	4848.3(16)	2165.3(11)	1211.1(13)	20.1(2)
C(3)	5567.2(15)	2548.0(11)	2930.7(12)	19.2(2)
C(4)	5267.4(15)	1589.7(11)	4015.6(13)	19.8(2)
C(5)	3180.7(15)	1344.5(12)	3563.2(13)	22.7(2)
C(6)	2378.9(16)	978.0(12)	1865.1(13)	23.7(3)
C(7)	2031.3(17)	1426.5(13)	-926.8(14)	28.3(3)
C(8)	6182.9(16)	1893.1(12)	5747.7(13)	22.5(2)
C(9)	5327.4(19)	2955.5(13)	6270.2(14)	31.1(3)
C(10)	6160.5(18)	842.1(13)	6774.6(14)	27.3(3)
C(11)	24.6(17)	3125.7(15)	253.8(15)	32.0(3)
C(12)	5848.8(16)	1113.8(11)	949.1(12)	22.2(2)
C(13)	7593.6(15)	2877.2(11)	3354.3(12)	20.4(2)
C(14)	9761.5(18)	4409.3(12)	4324.0(16)	28.3(3)
C(15)	10587.8(17)	4089.9(14)	6011.5(15)	30.6(3)
O(1)	5214.3(12)	3045.3(8)	249.9(9)	23.9(2)
O(2)	1979.9(11)	2991.4(8)	904.6(10)	25.3(2)
O(3)	8829.1(12)	2207.9(9)	3388.1(10)	26.7(2)
O(4)	7847.8(11)	4014.8(8)	3730.0(10)	24.3(2)
N(1)	6542.1(15)	285.2(11)	693.2(12)	28.4(2)

Table 3. Bond lengths [Å] and angles [°].

C(1)-O(2)	1.4308(15)	C(4)-C(8)	1.5486(15)
C(1)-C(7)	1.5292(16)	C(5)-C(6)	1.5306(15)
C(1)-C(6)	1.5344(16)	C(8)-C(9)	1.5261(19)
C(1)-C(2)	1.5616(15)	C(8)-C(10)	1.5288(18)
C(2)-O(1)	1.4199(14)	C(11)-O(2)	1.4228(14)
C(2)-C(12)	1.4844(17)	C(12)-N(1)	1.1455(17)
C(2)-C(3)	1.5496(14)	C(13)-O(3)	1.2053(15)
C(3)-C(13)	1.5119(15)	C(13)-O(4)	1.3467(16)
C(3)-C(4)	1.5421(15)	C(14)-O(4)	1.4537(14)
C(4)-C(5)	1.5326(14)	C(14)-C(15)	1.5096(18)
O(2)-C(1)-C(7)	112.27(10)	C(3)-C(2)-C(1)	109.93(9)
O(2)-C(1)-C(6)	111.59(10)	C(13)-C(3)-C(4)	111.08(9)
C(7)-C(1)-C(6)	111.00(10)	C(13)-C(3)-C(2)	109.44(9)
O(2)-C(1)-C(2)	101.02(9)	C(4)-C(3)-C(2)	111.69(9)
C(7)-C(1)-C(2)	110.49(9)	C(5)-C(4)-C(3)	107.99(9)
C(6)-C(1)-C(2)	110.06(9)	C(5)-C(4)-C(8)	114.05(9)
O(1)-C(2)-C(12)	105.52(9)	C(3)-C(4)-C(8)	112.51(9)
O(1)-C(2)-C(3)	110.29(10)	C(6)-C(5)-C(4)	111.63(9)
C(12)-C(2)-C(3)	110.98(9)	C(5)-C(6)-C(1)	113.16(10)
O(1)-C(2)-C(1)	111.59(9)	C(9)-C(8)-C(10)	110.48(10)
C(12)-C(2)-C(1)	108.46(10)	C(9)-C(8)-C(4)	113.50(10)

C(10)-C(8)-C(4)	110.96(10)	O(4)-C(13)-C(3)	111.04(10)
N(1)-C(12)-C(2)	176.64(12)	O(4)-C(14)-C(15)	110.34(10)
O(3)-C(13)-O(4)	124.21(10)	C(11)-O(2)-C(1)	116.97(10)
O(3)-C(13)-C(3)	124.72(12)	C(13)-O(4)-C(14)	116.22(9)

Table 4. Torsion angles [°].

O(2)-C(1)-C(2)-O(1)	-58.39(11)
C(7)-C(1)-C(2)-O(1)	60.62(13)
C(6)-C(1)-C(2)-O(1)	-176.44(10)
O(2)-C(1)-C(2)-C(12)	-174.19(9)
C(7)-C(1)-C(2)-C(12)	-55.18(12)
C(6)-C(1)-C(2)-C(12)	67.75(11)
O(2)-C(1)-C(2)-C(3)	64.31(11)
C(7)-C(1)-C(2)-C(3)	-176.68(10)
C(6)-C(1)-C(2)-C(3)	-53.75(13)
O(1)-C(2)-C(3)-C(13)	-54.13(13)
C(12)-C(2)-C(3)-C(13)	62.43(13)
C(1)-C(2)-C(3)-C(13)	-177.59(10)
O(1)-C(2)-C(3)-C(4)	-177.56(9)
C(12)-C(2)-C(3)-C(4)	-61.00(12)
C(1)-C(2)-C(3)-C(4)	58.99(12)
C(13)-C(3)-C(4)-C(5)	177.44(10)
C(2)-C(3)-C(4)-C(5)	-60.07(12)
C(13)-C(3)-C(4)-C(8)	50.69(12)
C(2)-C(3)-C(4)-C(8)	173.19(9)
C(3)-C(4)-C(5)-C(6)	57.63(13)
C(8)-C(4)-C(5)-C(6)	-176.53(10)
C(4)-C(5)-C(6)-C(1)	-56.29(14)
O(2)-C(1)-C(6)-C(5)	-58.06(12)
C(7)-C(1)-C(6)-C(5)	175.88(10)
C(2)-C(1)-C(6)-C(5)	53.24(13)
C(5)-C(4)-C(8)-C(9)	-58.39(14)
C(3)-C(4)-C(8)-C(9)	65.04(12)
C(5)-C(4)-C(8)-C(10)	66.72(13)
C(3)-C(4)-C(8)-C(10)	-169.85(9)
C(4)-C(3)-C(13)-O(3)	58.31(14)
C(2)-C(3)-C(13)-O(3)	-65.47(14)
C(4)-C(3)-C(13)-O(4)	-120.08(10)
C(2)-C(3)-C(13)-O(4)	116.14(10)
C(7)-C(1)-O(2)-C(11)	49.16(13)
C(6)-C(1)-O(2)-C(11)	-76.19(12)
C(2)-C(1)-O(2)-C(11)	166.88(9)
O(3)-C(13)-O(4)-C(14)	-4.96(15)
C(3)-C(13)-O(4)-C(14)	173.44(9)
C(15)-C(14)-O(4)-C(13)	-79.73(13)

Table 5. Hydrogen bonds [Å and °].

D-H...A	d(D-H)	d(H...A)	d(D...A)	<(DHA)
O(1)-H(01)...O(2)	0.82(2)	2.211(19)	2.7072(12)	119.4(19)
O(1)-H(01)...N(1)#1	0.82(2)	2.21(2)	2.8961(16)	141.4(19)

Symmetry transformations used to generate equivalent atoms:
#1 -x+1,y+1/2,-z

NMR spectra

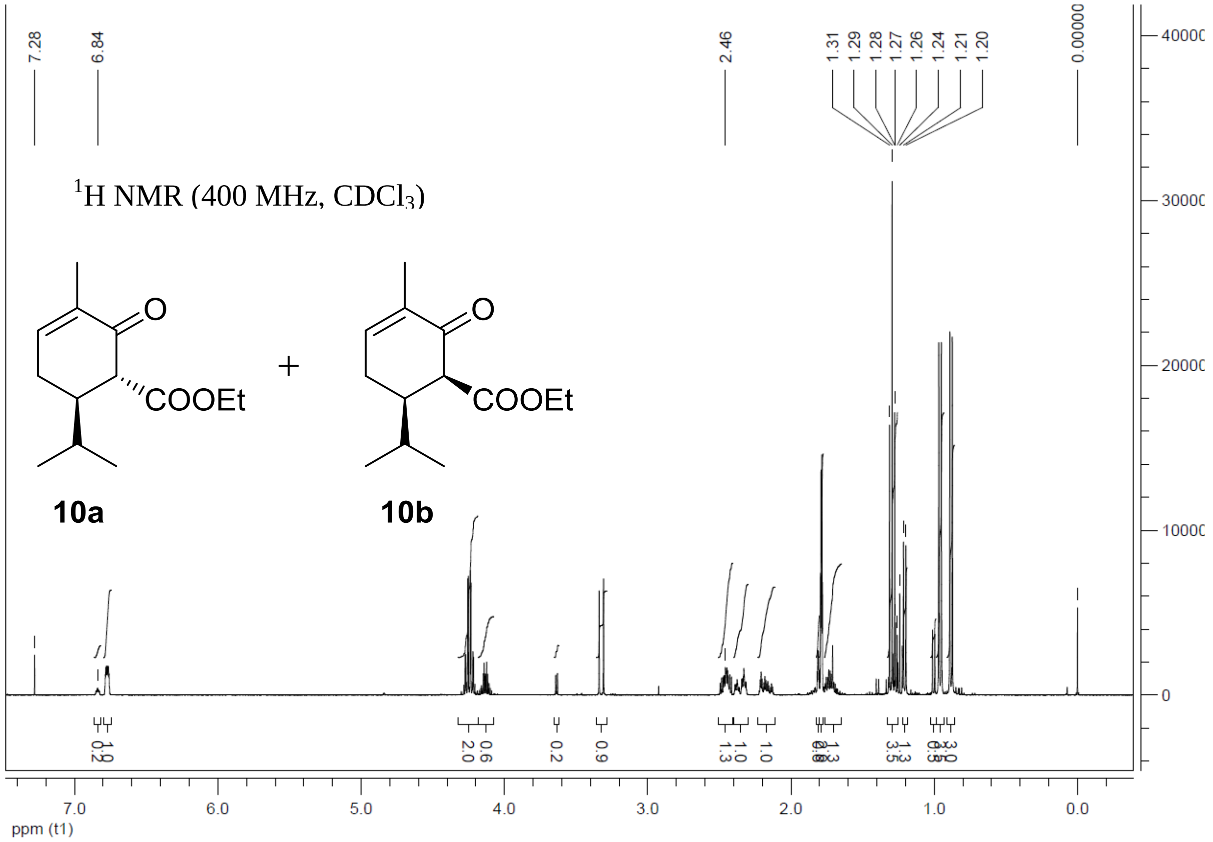

7.28
6.84
2.46
1.31
1.29
1.28
1.27
1.26
1.24
1.21
1.20
0.00000
^{1}H NMR (400 MHz, CDCl$_3$)
COOEt
+
COOEt
10a
10b
ppm (t1)

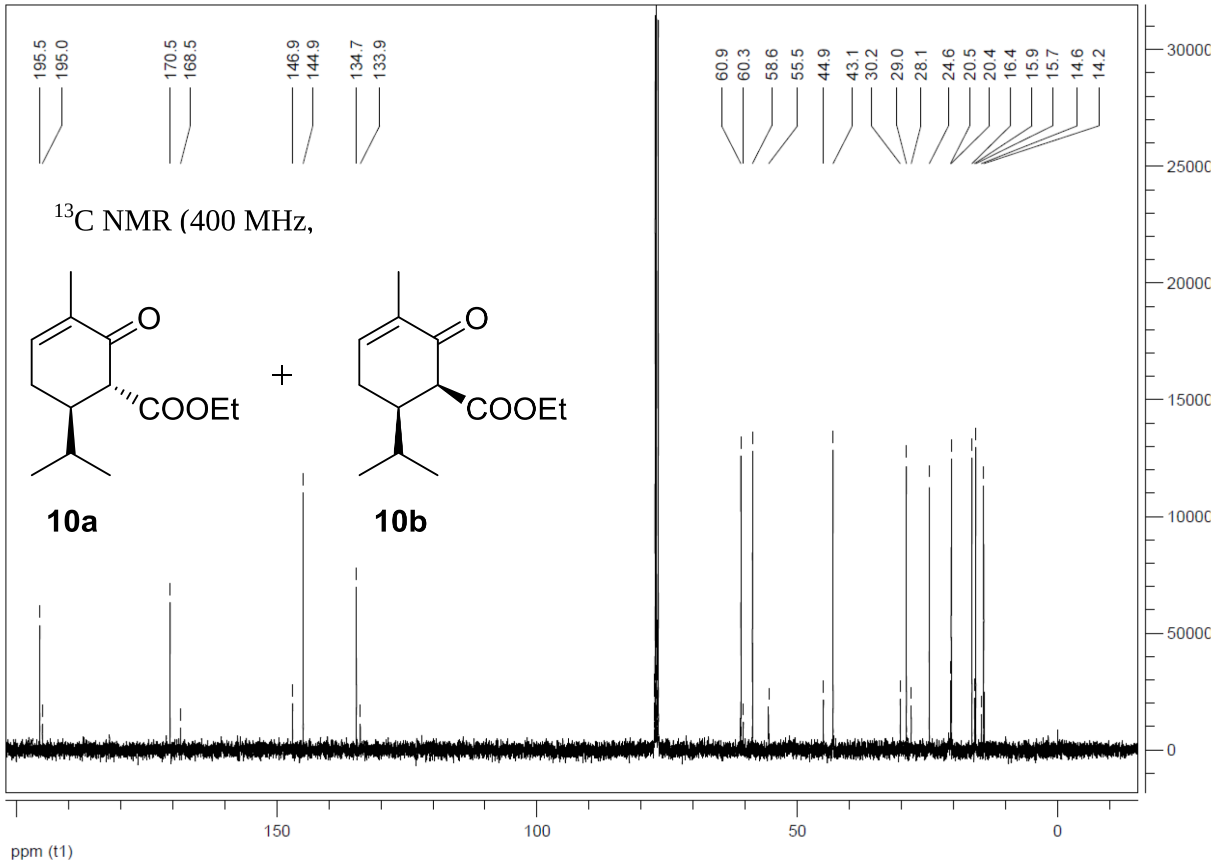

195.5
195.0
170.5
168.5
146.9
144.9
134.7
133.9
60.9
60.3
58.6
55.5
44.9
43.1
30.2
29.0
28.1
24.6
20.5
20.4
16.4
15.9
15.7
14.6
14.2
^{13}C NMR (400 MHz,
COOEt
+
COOEt
10a
10b
ppm (t1)

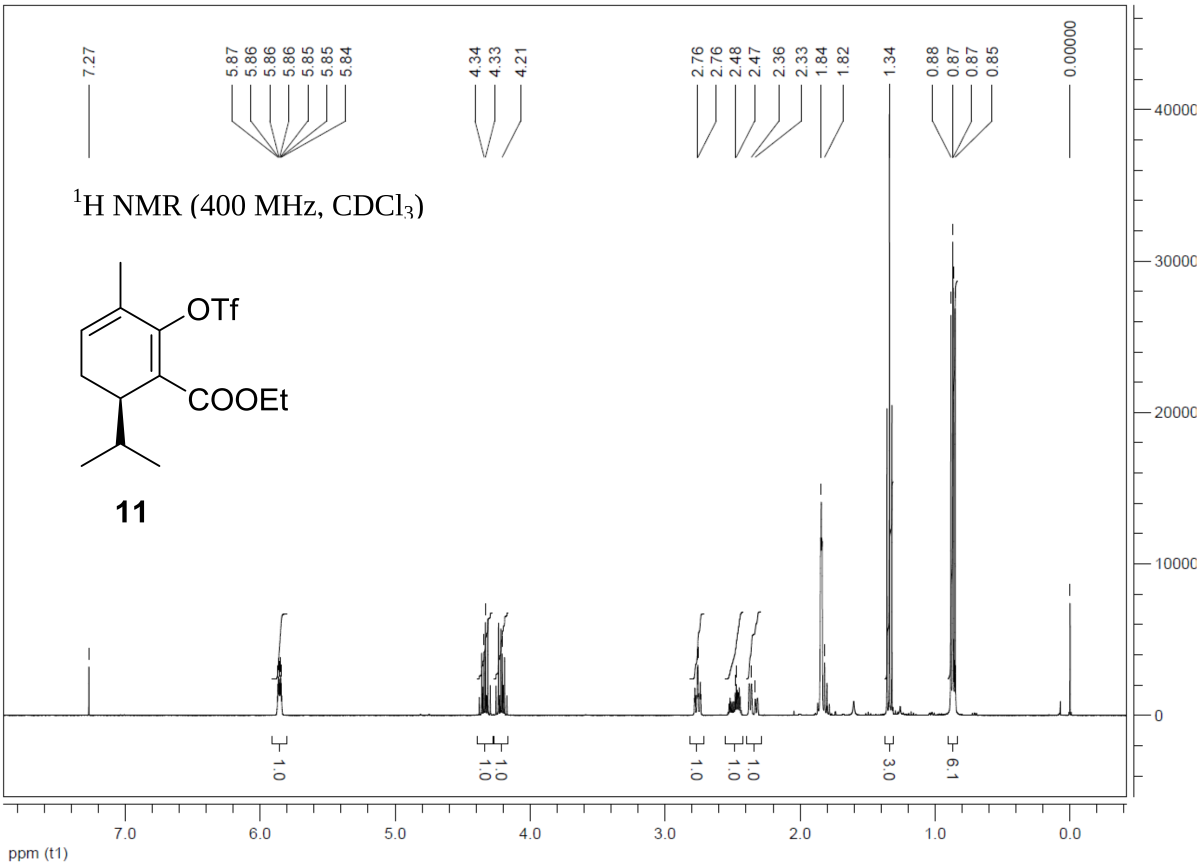

7.27
5.87 5.86 5.86 5.86 5.85 5.85 5.84
4.34 4.33 4.21
2.76 2.76 2.48 2.47 2.36 2.33 1.84 1.82
1.34
0.88 0.87 0.87 0.85
0.00000
1H NMR (400 MHz, CDCl3)
OTf
COOEt
11
1.0 1.0 1.0 1.0 1.0 1.0 3.0 6.1
40000 30000 20000 10000 0
7.0 6.0 5.0 4.0 3.0 2.0 1.0 0.0
ppm (t1)

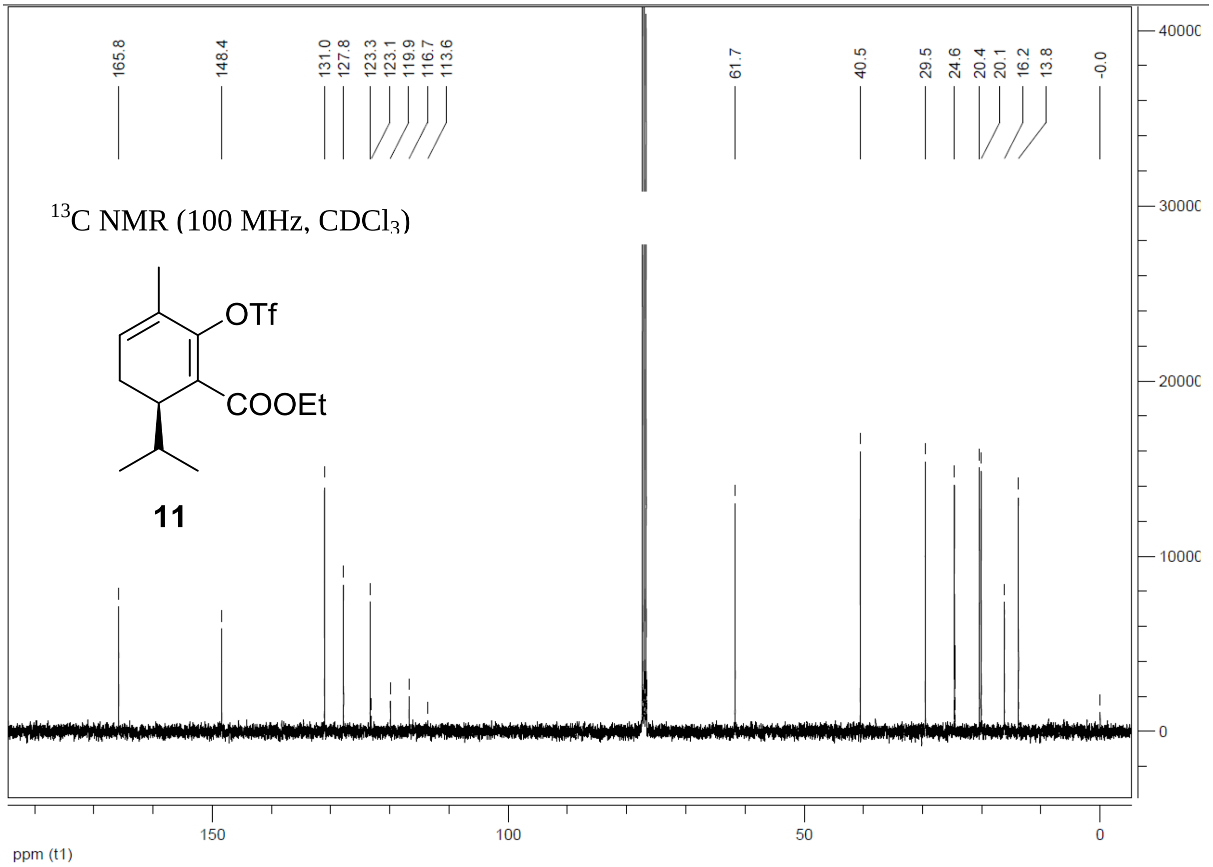

165.8
148.4
131.0 127.8 123.3 123.1 119.9 116.7 113.6
61.7
40.5
29.5 24.6 20.4 20.1 16.2 13.8
-0.0
13C NMR (100 MHz, CDCl3)
OTf
COOEt
11
40000 30000 20000 10000 0
150 100 50 0
ppm (t1)

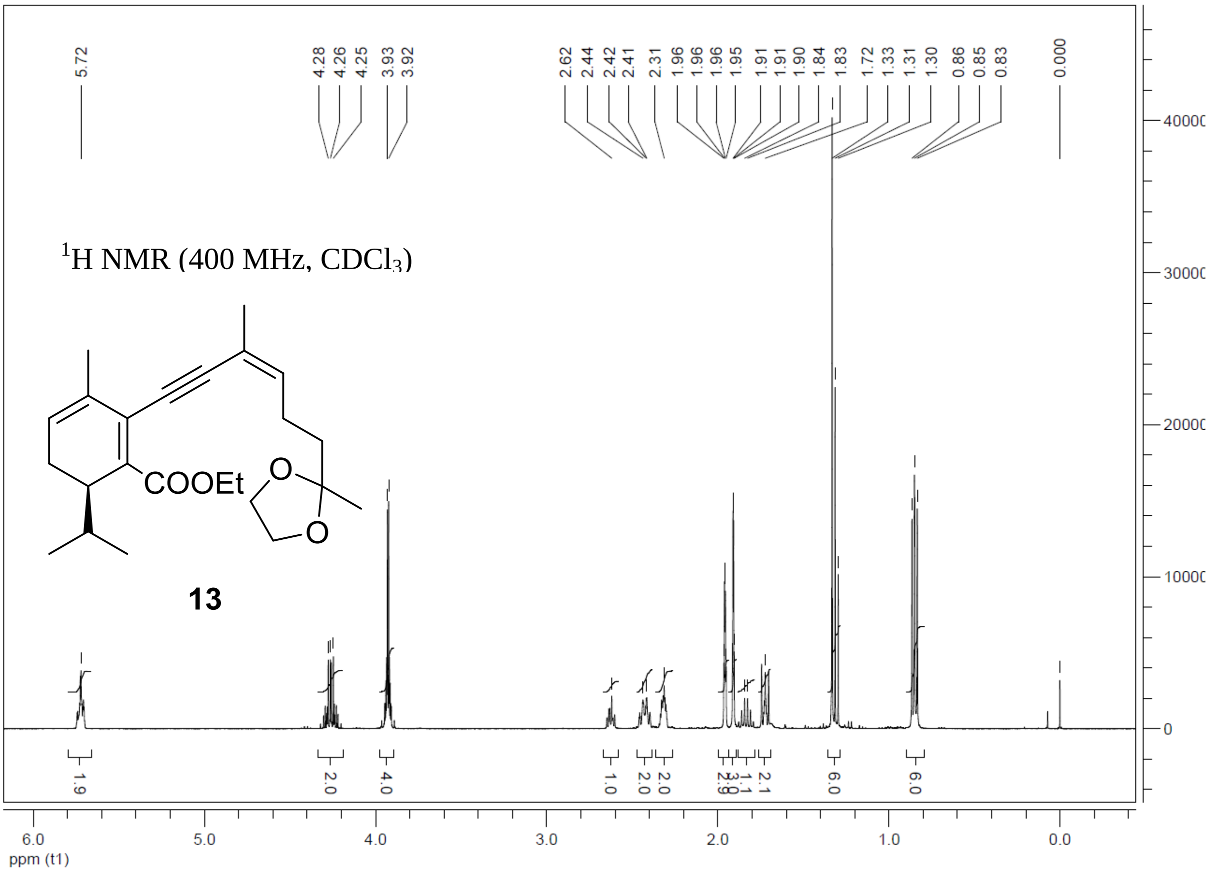

5.72
4.28
4.26
4.25
3.93
3.92
2.62
2.44
2.42
2.41
2.31
1.96
1.96
1.96
1.95
1.91
1.91
1.90
1.84
1.83
1.72
1.33
1.31
1.30
0.86
0.85
0.83
0.000
^{1}H NMR (400 MHz, CDCl$_3$)
COOEt
O
O
13
1.9
2.0
4.0
1.0
2.0
2.0
2.9
3.0
1.1
2.1
6.0
6.0
ppm (t1)

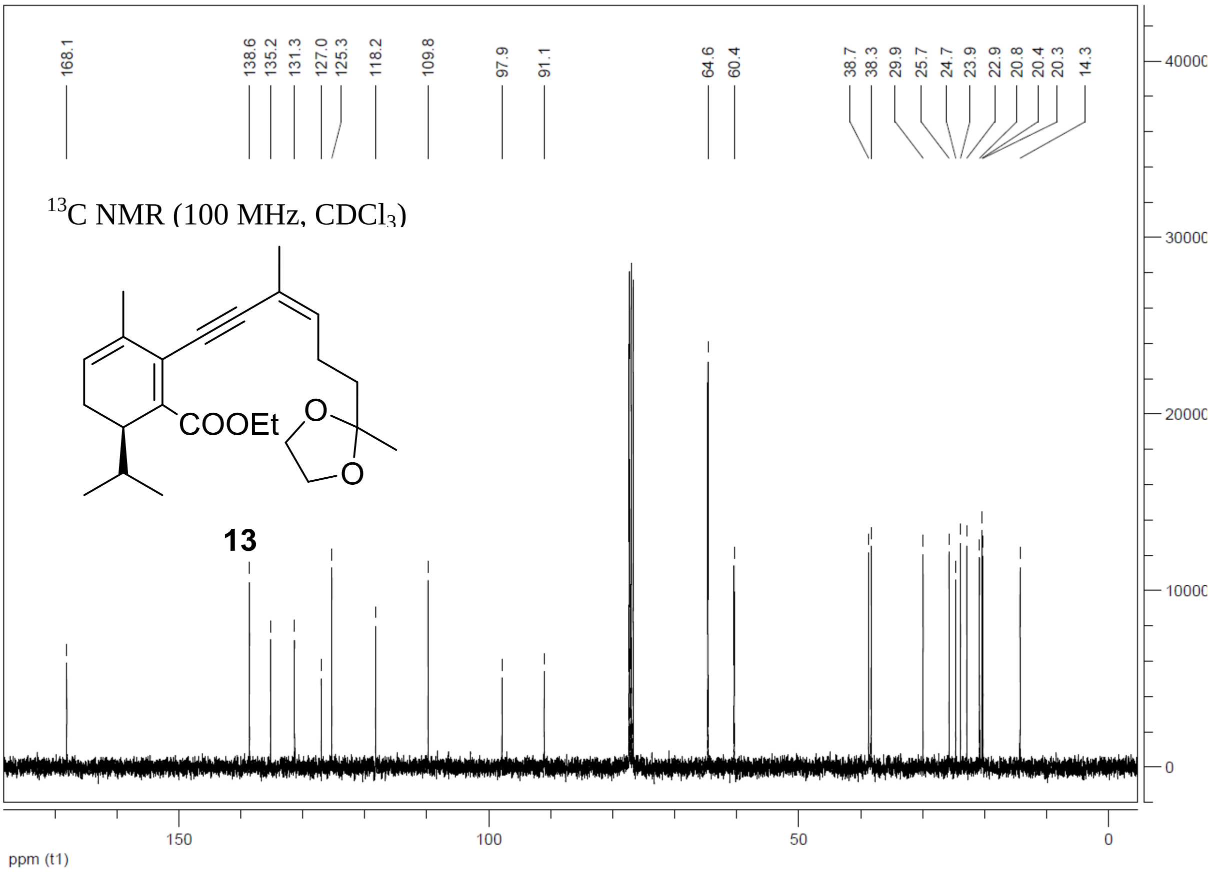

168.1
138.6
135.2
131.3
127.0
125.3
118.2
109.8
97.9
91.1
64.6
60.4
38.7
38.3
29.9
25.7
24.7
23.9
22.9
20.8
20.4
20.3
14.3
^{13}C NMR (100 MHz, CDCl$_3$)
COOEt
O
O
13
ppm (t1)

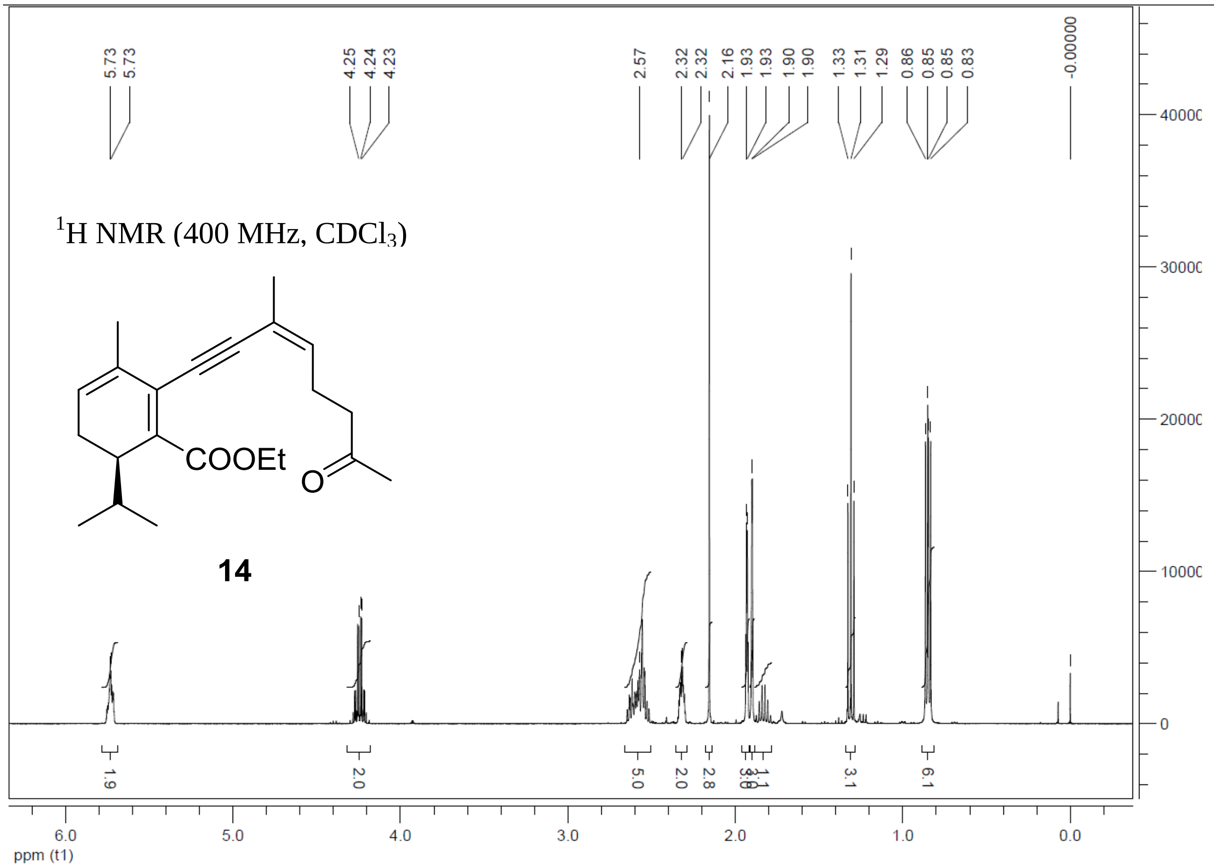

^{1}H NMR (400 MHz, $CDCl_3$)
COOEt
O
14
ppm (t1)

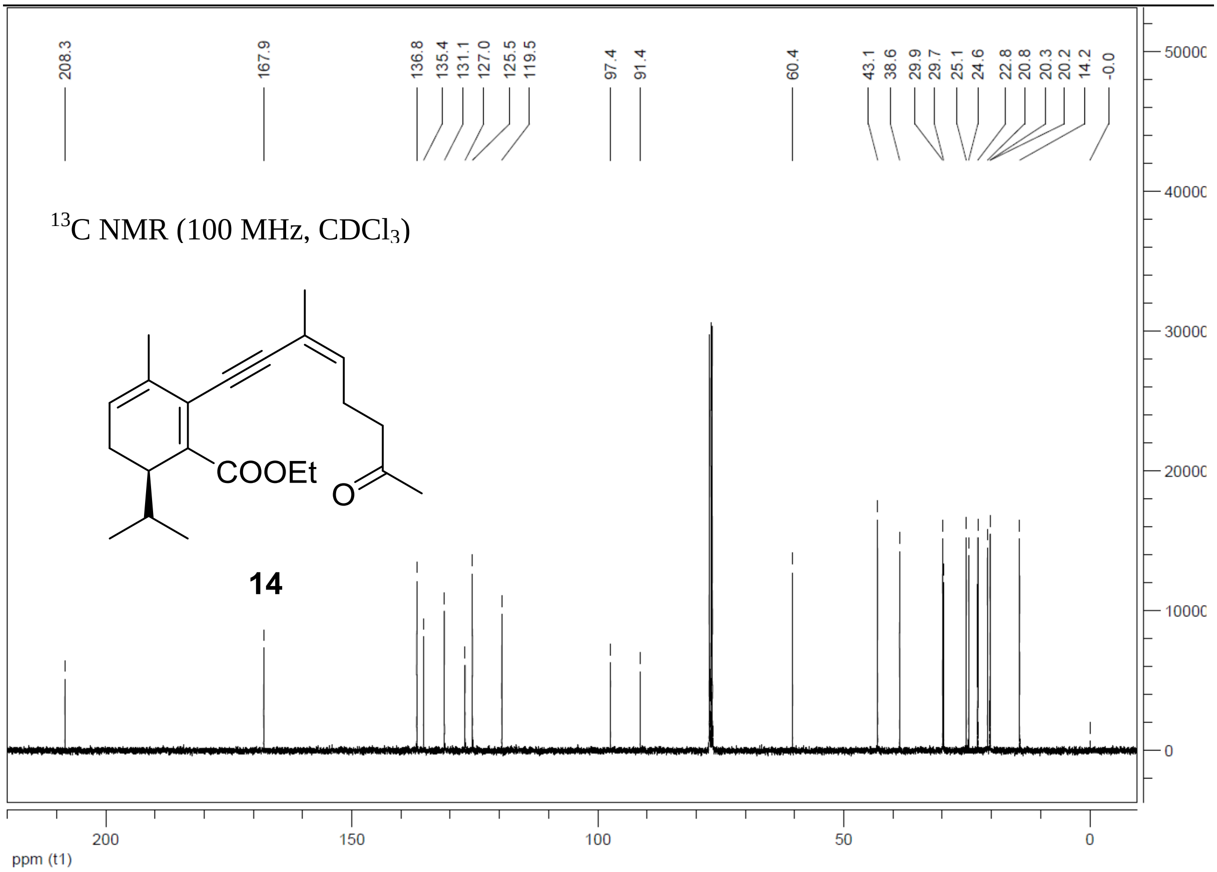

^{13}C NMR (100 MHz, $CDCl_3$)
COOEt
O
14
ppm (t1)

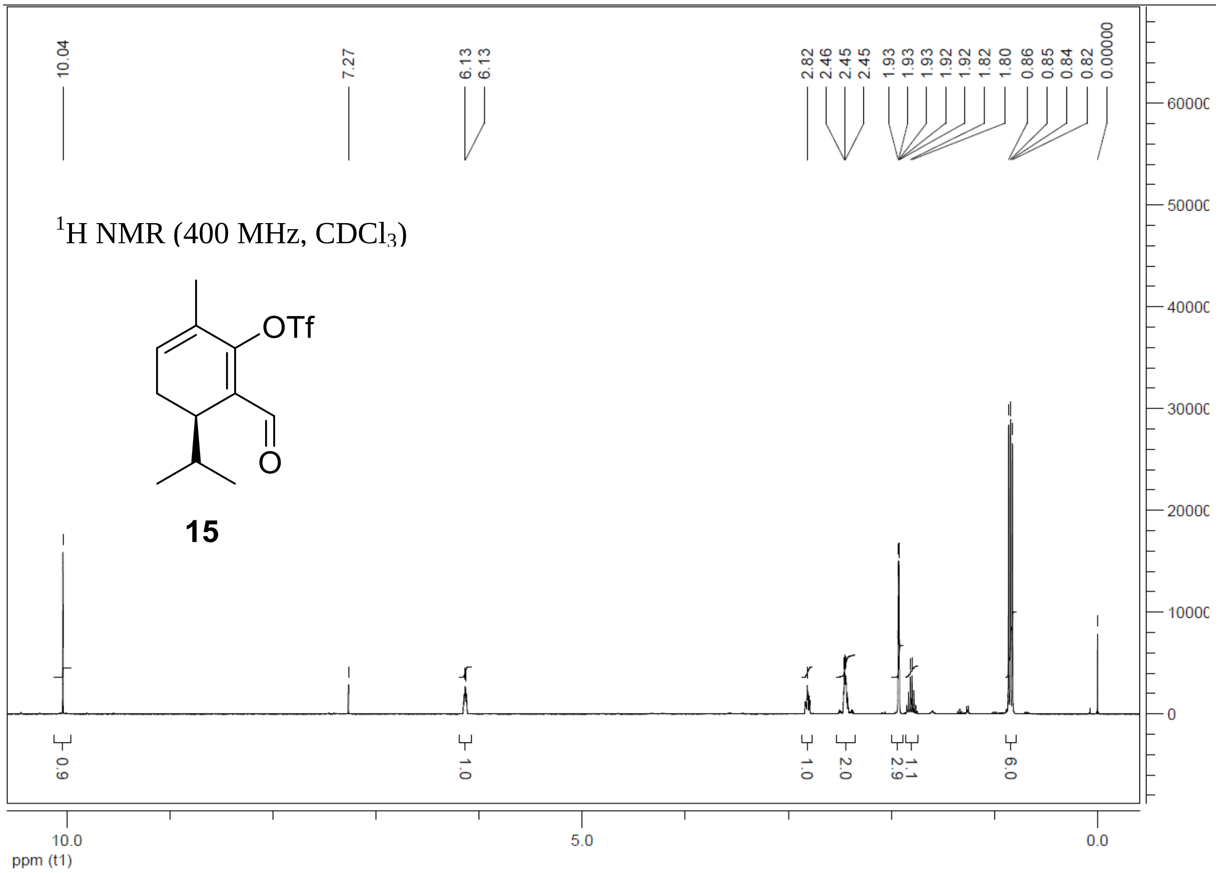
10.04
7.27
6.13
6.13
2.82
2.46
2.45
2.45
1.93
1.93
1.93
1.92
1.92
1.82
1.80
0.86
0.85
0.84
0.82
0.00000
1H NMR (400 MHz, CDCl3)
OTf
O
15
0.9
1.0
1.0
2.0
2.9
1.1
6.0
10.0
5.0
0.0
ppm (t1)

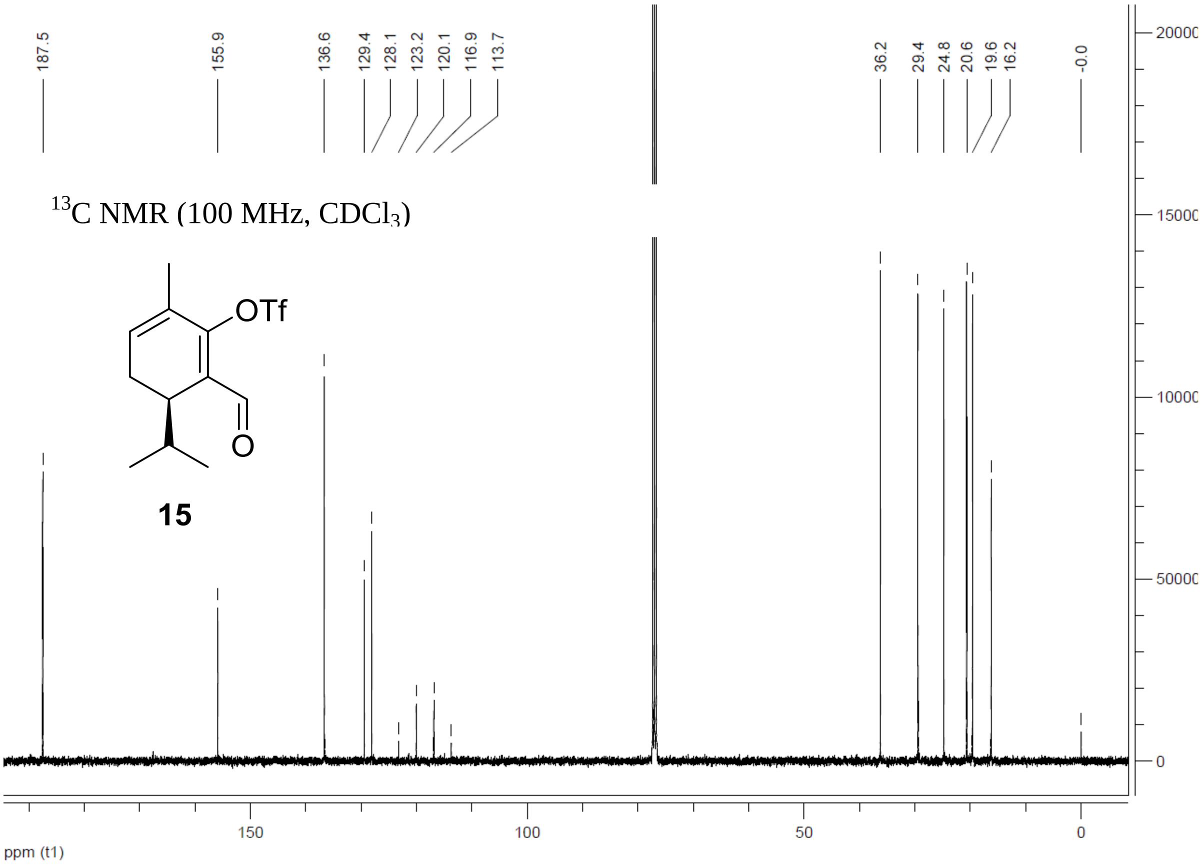
187.5
155.9
136.6
129.4
128.1
123.2
120.1
116.9
113.7
36.2
29.4
24.8
20.6
19.6
16.2
-0.0
13C NMR (100 MHz, CDCl3)
OTf
O
15
150
100
50
0
ppm (t1)

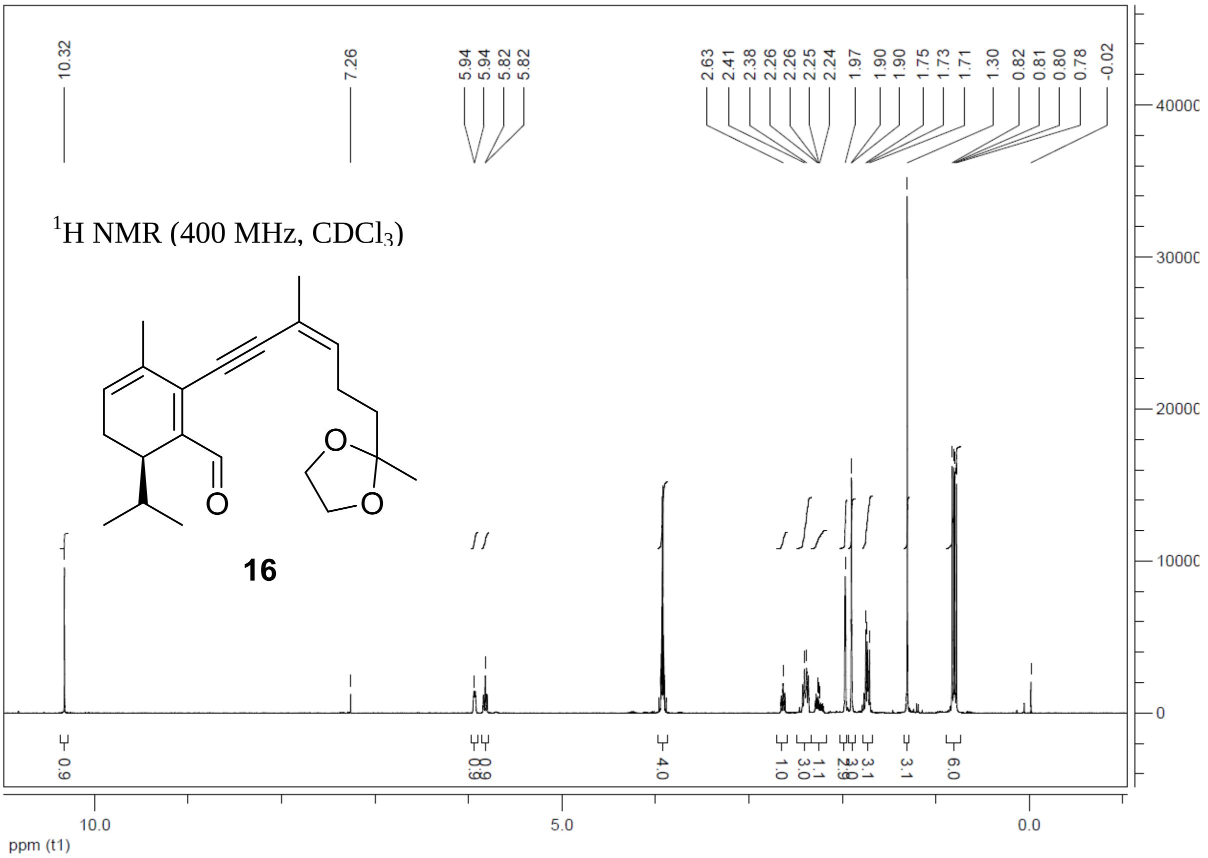

1H NMR (400 MHz, CDCl3)
16
10.32
7.26
5.94
5.94
5.82
5.82
2.63
2.41
2.38
2.26
2.26
2.25
2.24
1.97
1.90
1.90
1.75
1.73
1.71
1.30
0.82
0.81
0.80
0.78
-0.02
ppm (t1)

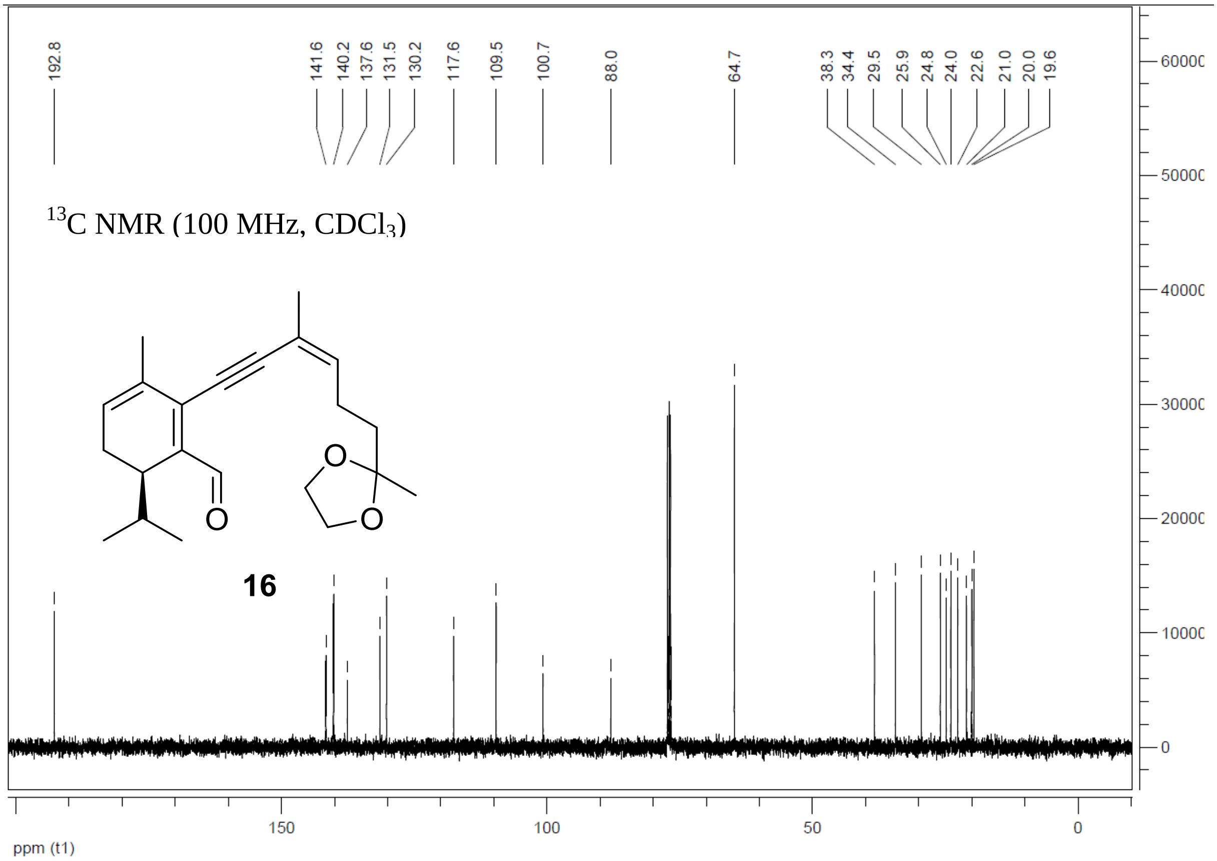

13C NMR (100 MHz, CDCl3)
16
192.8
141.6
140.2
137.6
131.5
130.2
117.6
109.5
100.7
88.0
64.7
38.3
34.4
29.5
25.9
24.8
24.0
22.6
21.0
20.0
19.6
ppm (t1)

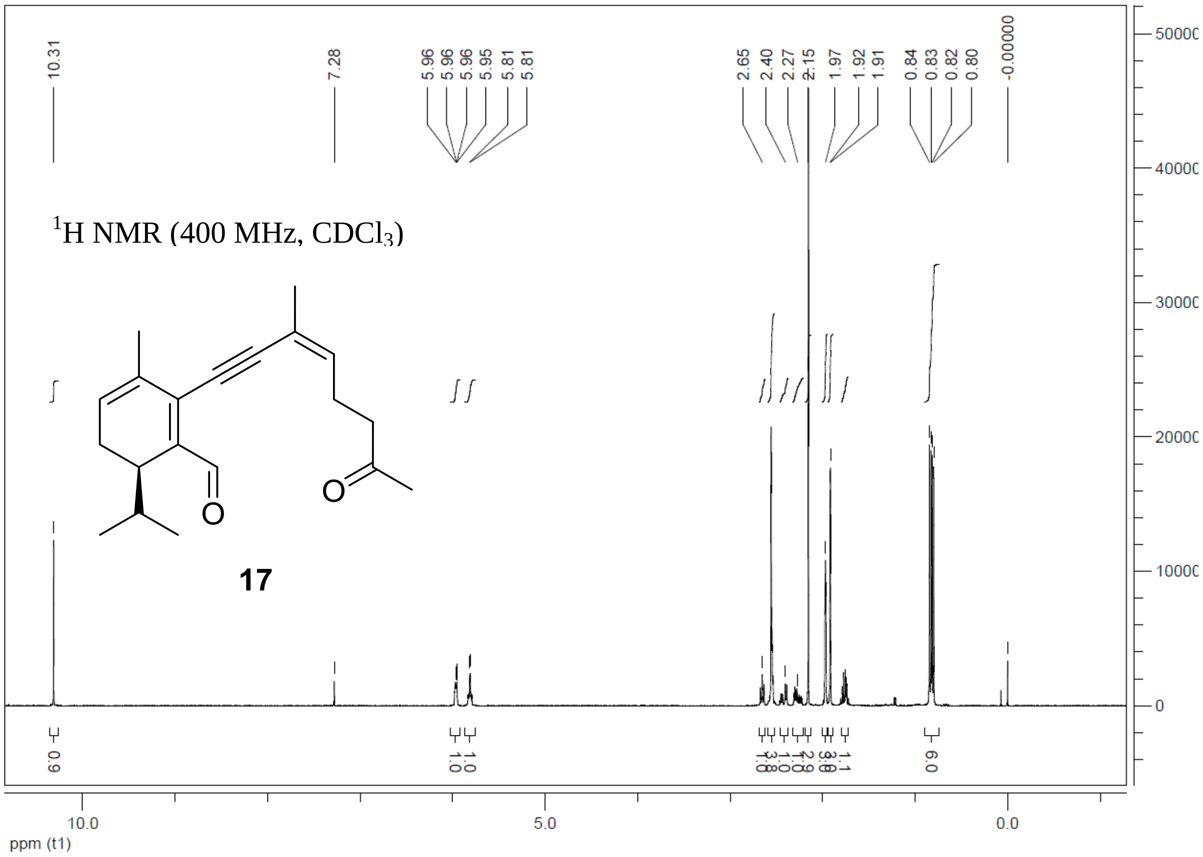
1H NMR (400 MHz, CDCl3)
17
10.31
7.28
5.96 5.96 5.96 5.95 5.81 5.81
2.65 2.40 2.27 2.15 1.97 1.92 1.91
0.84 0.83 0.82 0.80
-0.00000
ppm (t1)

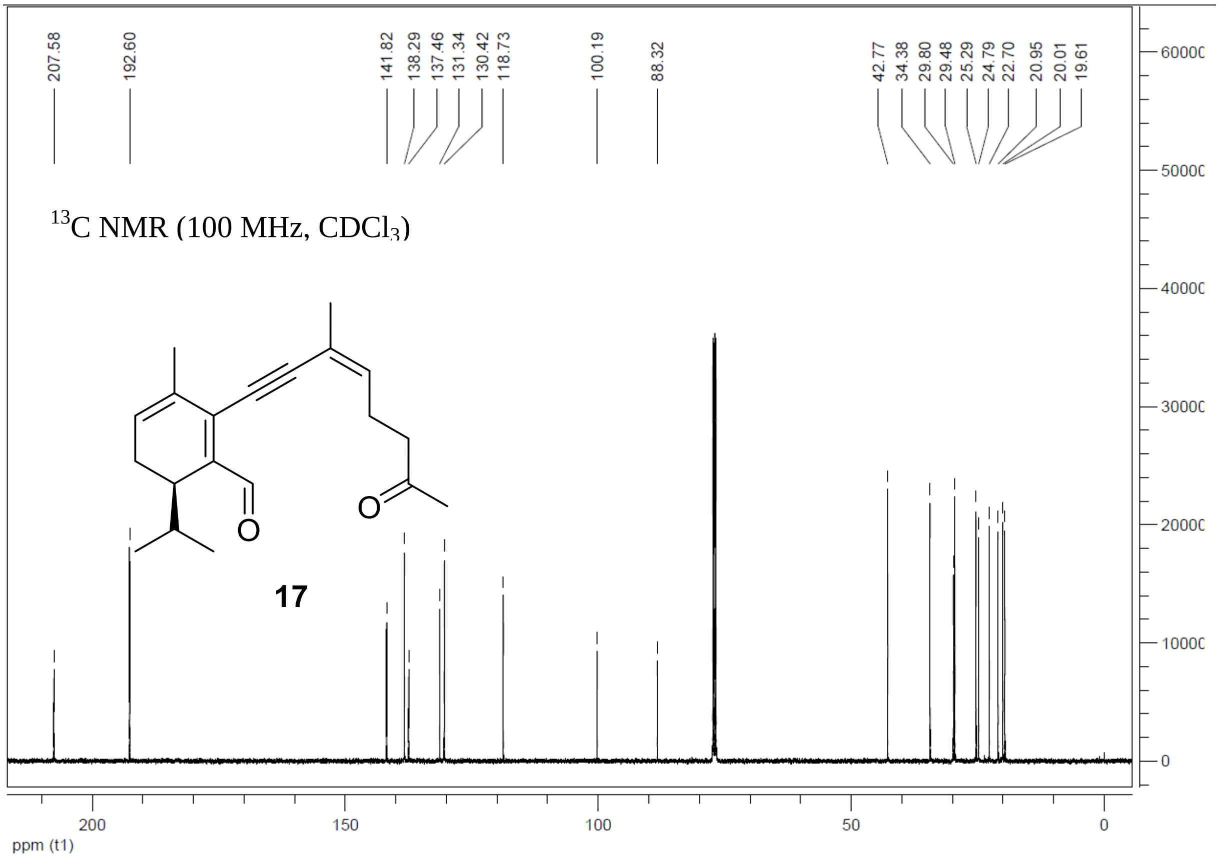
13C NMR (100 MHz, CDCl3)
17
207.58
192.60
141.82 138.29 137.46 131.34 130.42 118.73
100.19
88.32
42.77 34.38 29.80 29.48 25.29 24.79 22.70 20.95 20.01 19.61
ppm (t1)

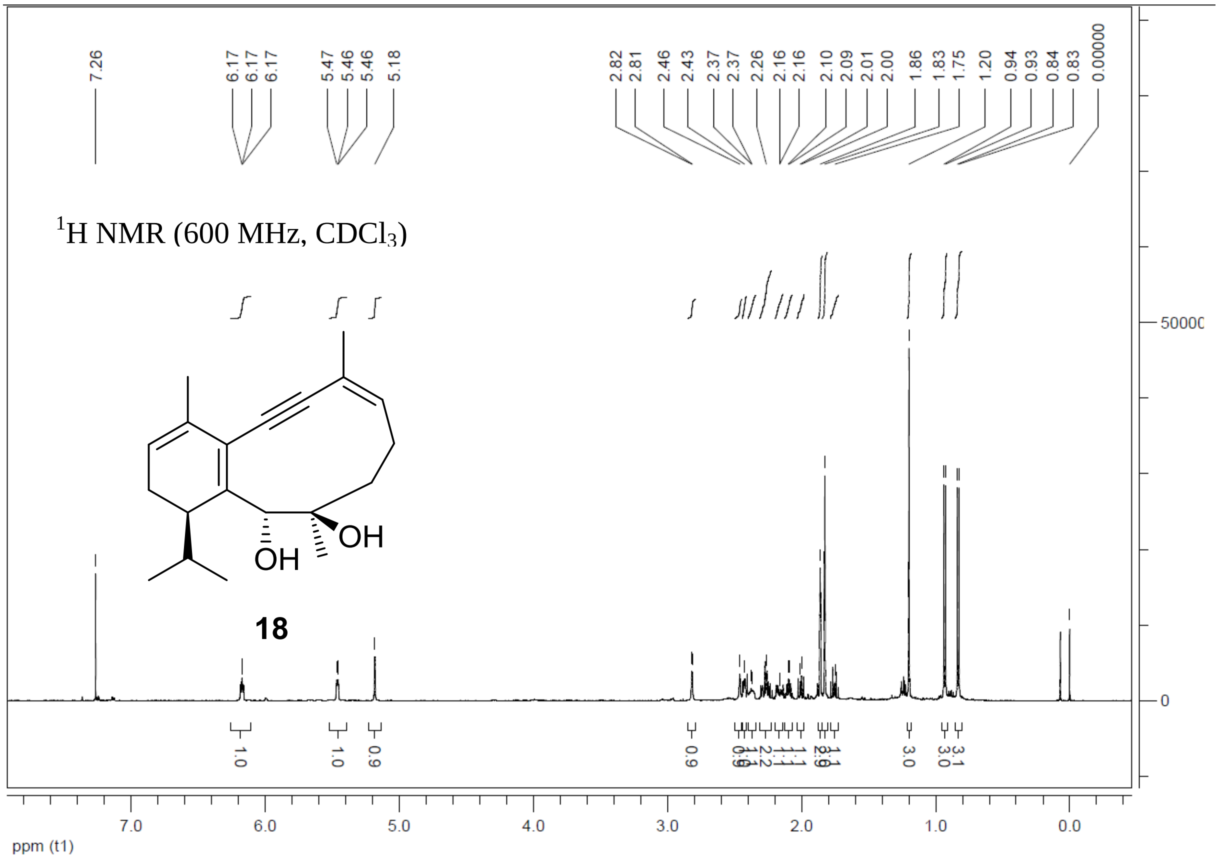

7.26
6.17 6.17 6.17
5.47 5.46 5.46 5.18
2.82 2.81 2.46 2.43 2.37 2.37 2.26 2.16 2.16 2.10 2.09 2.01 2.00 1.86 1.83 1.75 1.20 0.94 0.93 0.84 0.83 0.00000
1H NMR (600 MHz, CDCl3)
OH
OH
18
1.0 1.0 0.9 0.9 3.0 3.0 3.1
ppm (t1)

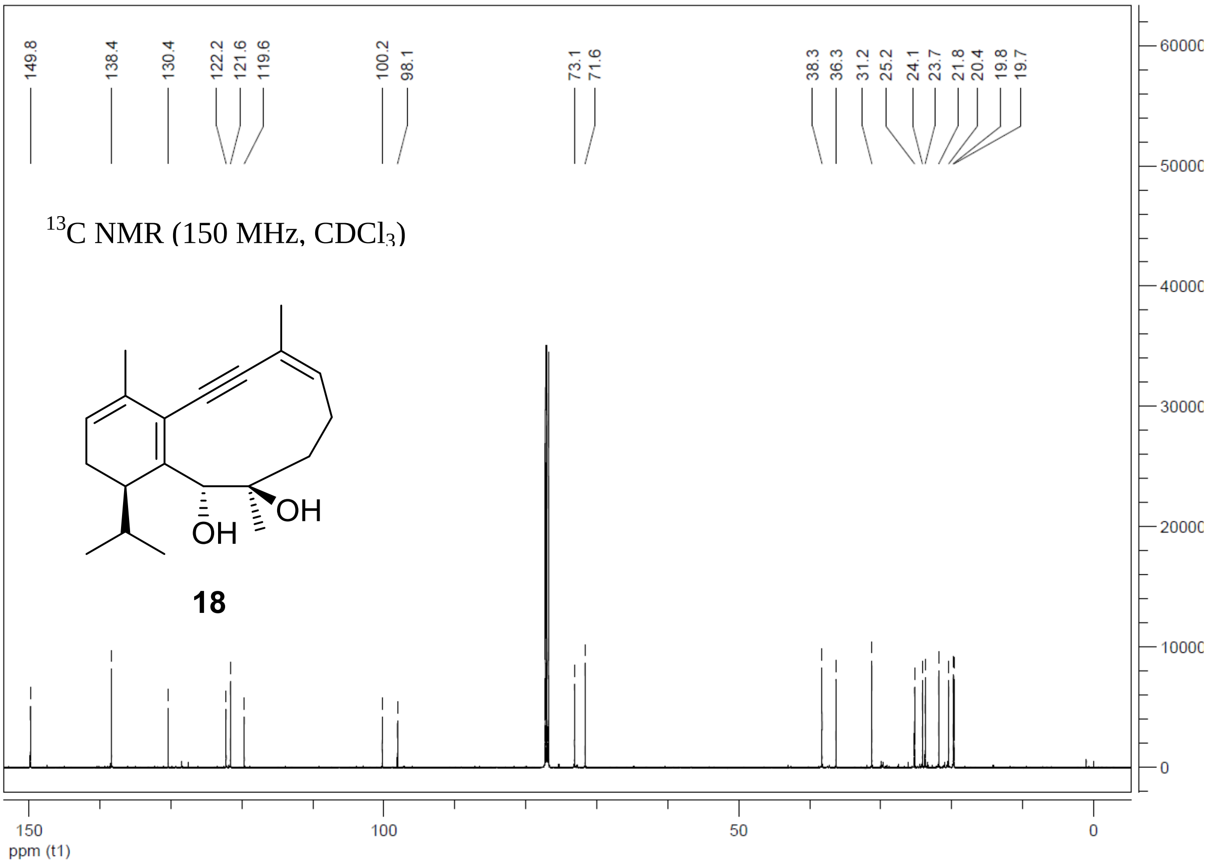

149.8 138.4 130.4 122.2 121.6 119.6 100.2 98.1 73.1 71.6 38.3 36.3 31.2 25.2 24.1 23.7 21.8 20.4 19.8 19.7
13C NMR (150 MHz, CDCl3)
OH
OH
18
ppm (t1)

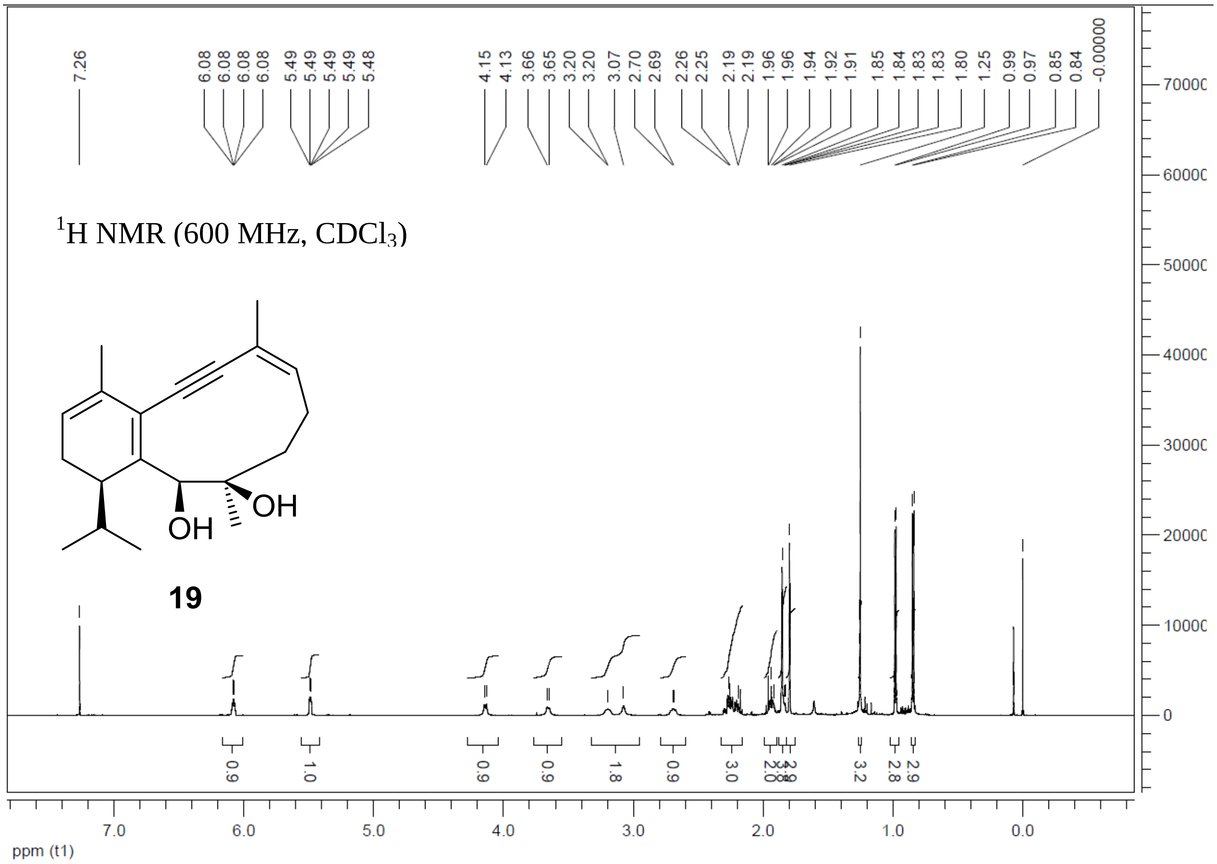

7.26
6.08 6.08 6.08 6.08 5.49 5.49 5.49 5.49 5.48
4.15 4.13 3.66 3.65 3.20 3.20 3.07 2.70 2.69 2.26 2.25 2.19 2.19 1.96 1.96 1.94 1.92 1.91 1.85 1.84 1.83 1.83 1.80 1.25 0.99 0.97 0.85 0.84 -0.00000
¹H NMR (600 MHz, CDCl₃)
OH
OH
19
0.9 1.0 0.9 0.9 1.8 0.9 3.0 2.0 3.2 2.8 2.9
ppm (t1)

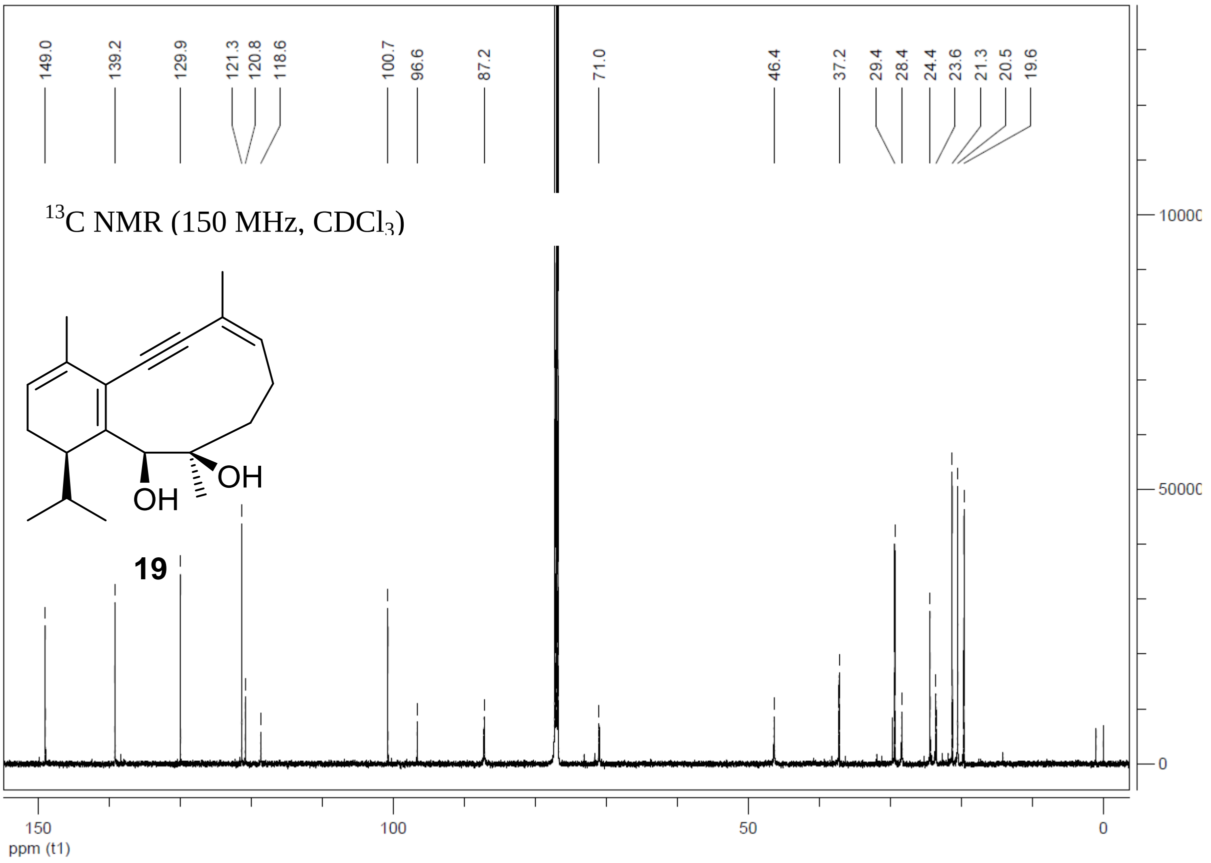

149.0 139.2 129.9 121.3 120.8 118.6 100.7 96.6 87.2 71.0 46.4 37.2 29.4 28.4 24.4 23.6 21.3 20.5 19.6
¹³C NMR (150 MHz, CDCl₃)
OH
OH
19
ppm (t1)

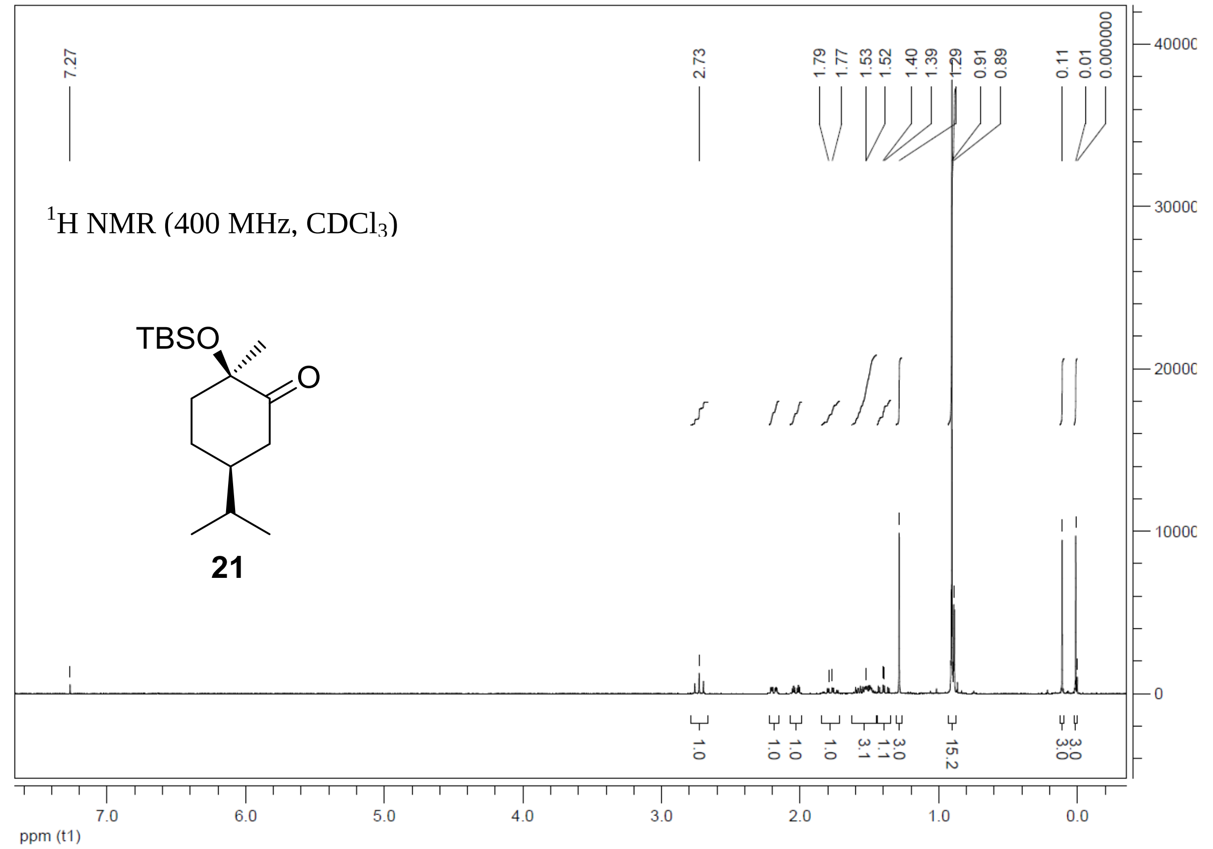

1H NMR (400 MHz, CDCl3)
TBSO
O
21
ppm (t1)

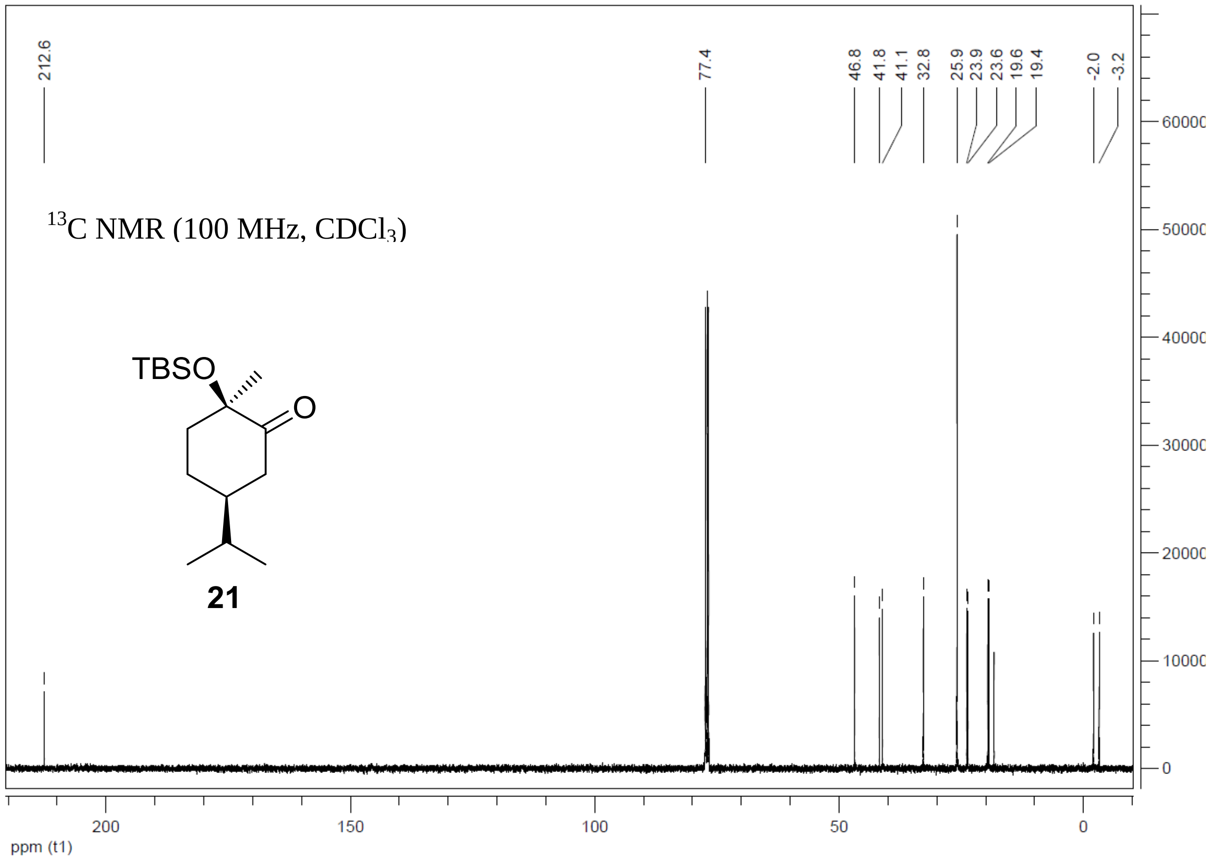

13C NMR (100 MHz, CDCl3)
TBSO
O
21
ppm (t1)

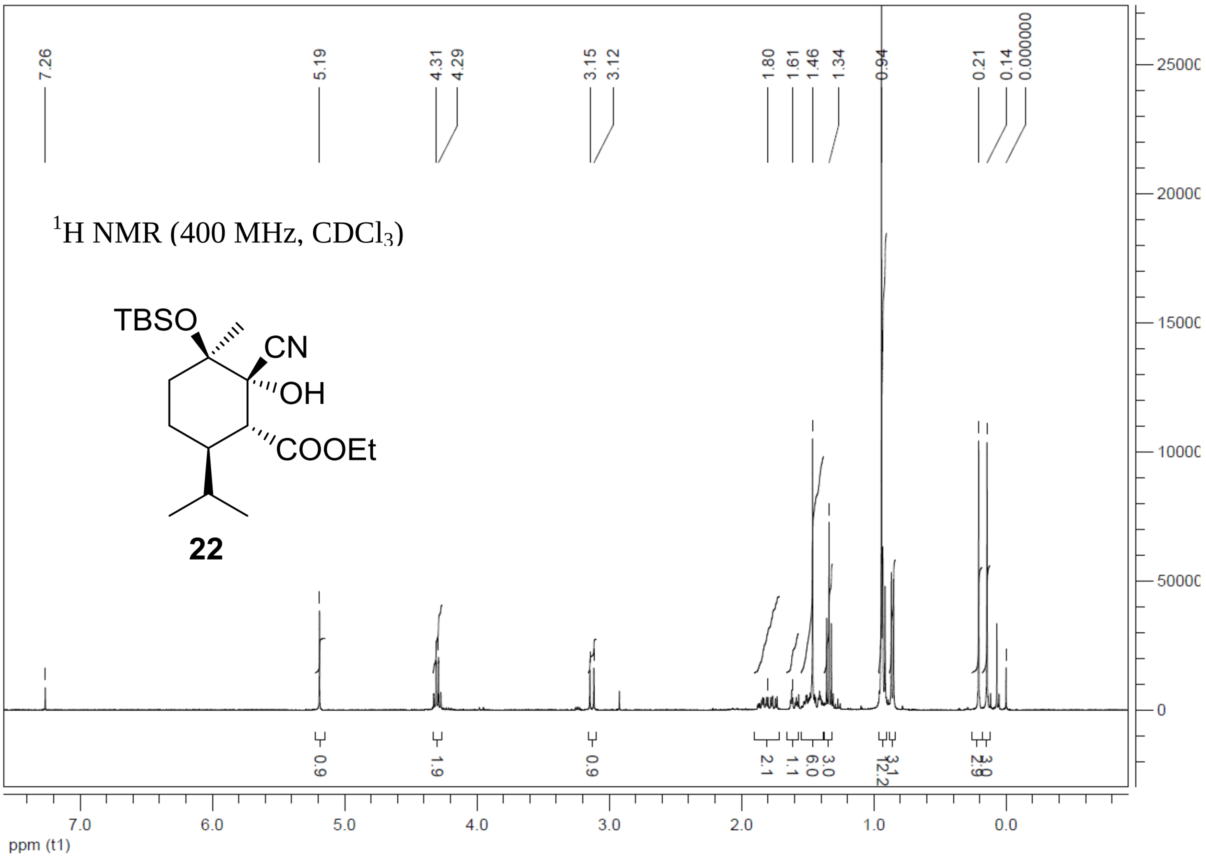
7.26
5.19
4.31
4.29
3.15
3.12
1.80
1.61
1.46
1.34
0.94
0.21
0.14
0.000000
^{1}H NMR (400 MHz, $CDCl_3$)
TBSO
CN
OH
COOEt
22
0.9
1.9
0.9
2.1
1.1
6.0
3.0
12.2
3.1
2.9
3.0
ppm (t1)

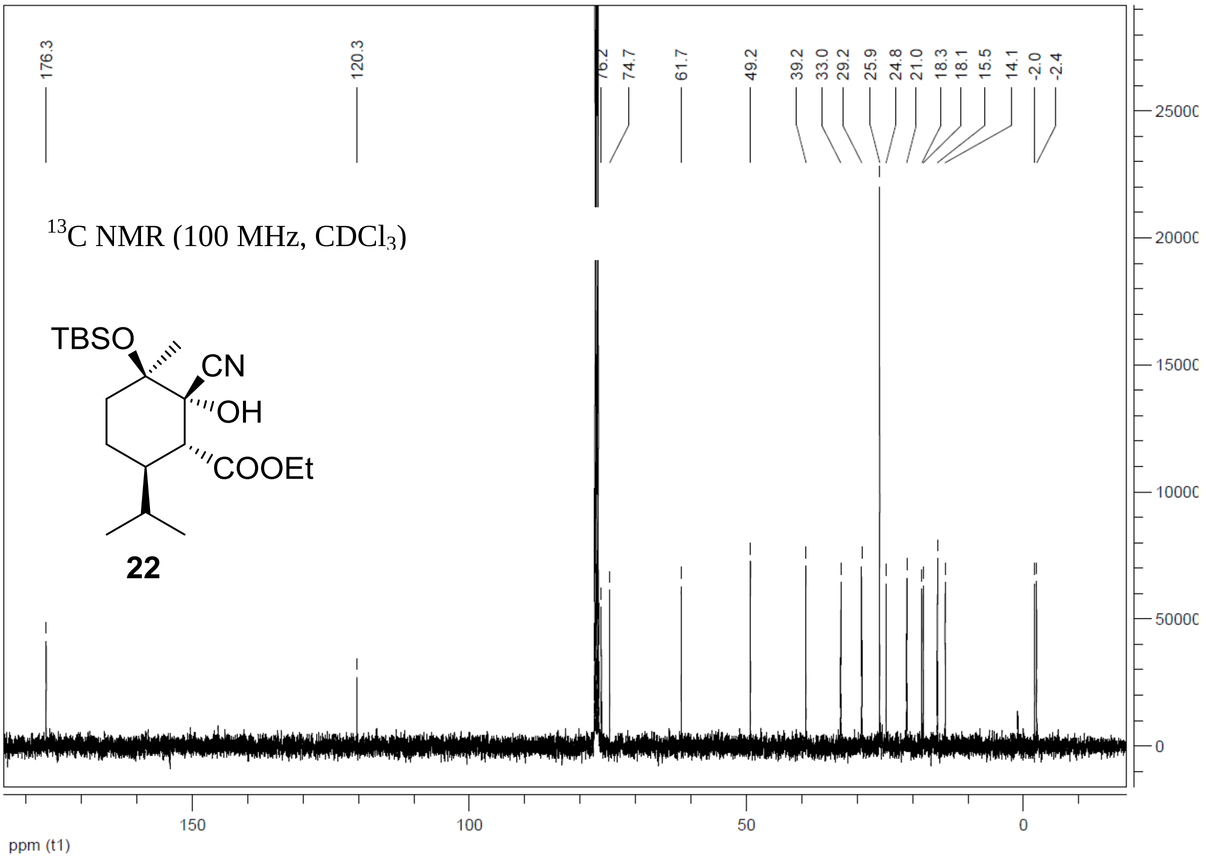
176.3
120.3
76.2
74.7
61.7
49.2
39.2
33.0
29.2
25.9
24.8
21.0
18.3
18.1
15.5
14.1
-2.0
-2.4
^{13}C NMR (100 MHz, $CDCl_3$)
TBSO
CN
OH
COOEt
22
ppm (t1)

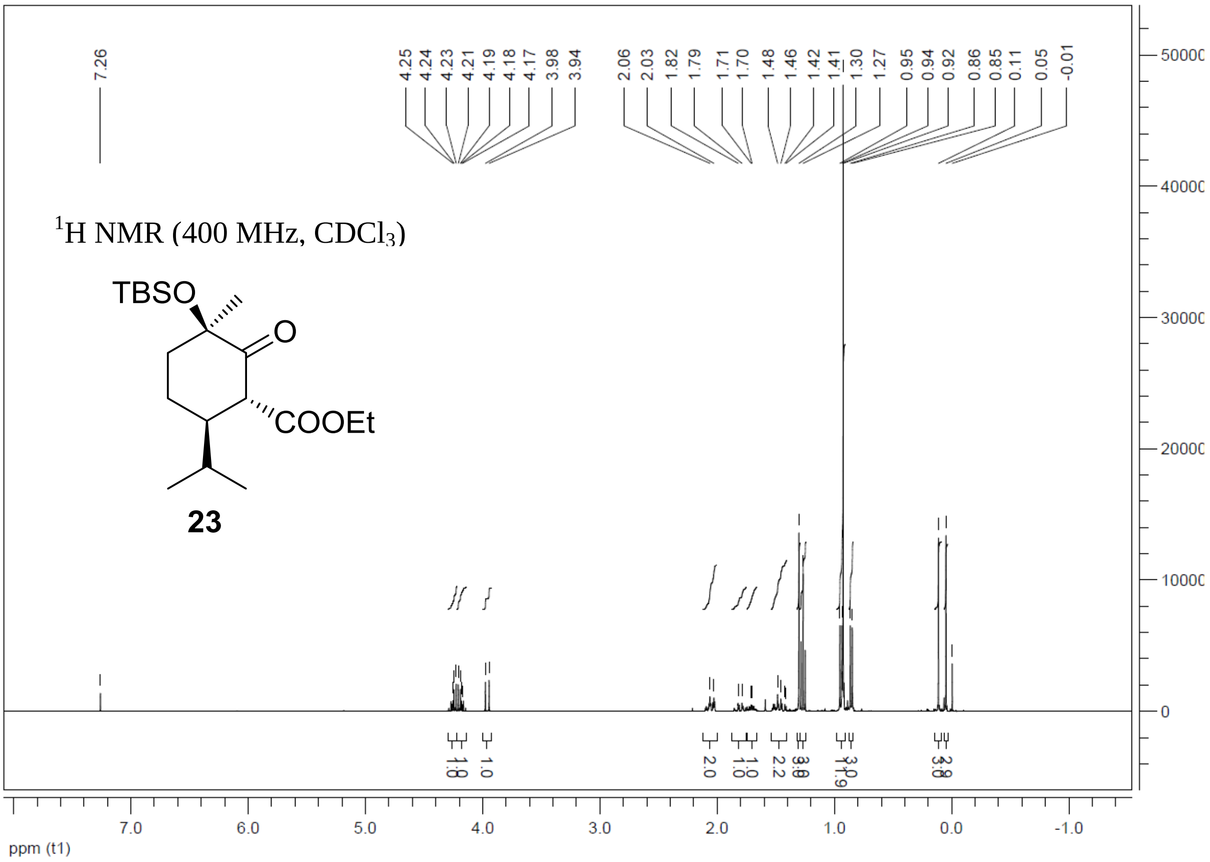
7.26
4.25
4.24
4.23
4.21
4.19
4.18
4.17
3.98
3.94
2.06
2.03
1.82
1.79
1.71
1.70
1.48
1.46
1.42
1.41
1.30
1.27
0.95
0.94
0.92
0.86
0.85
0.11
0.05
-0.01
1H NMR (400 MHz, CDCl3)
TBSO
O
COOEt
23
ppm (t1)

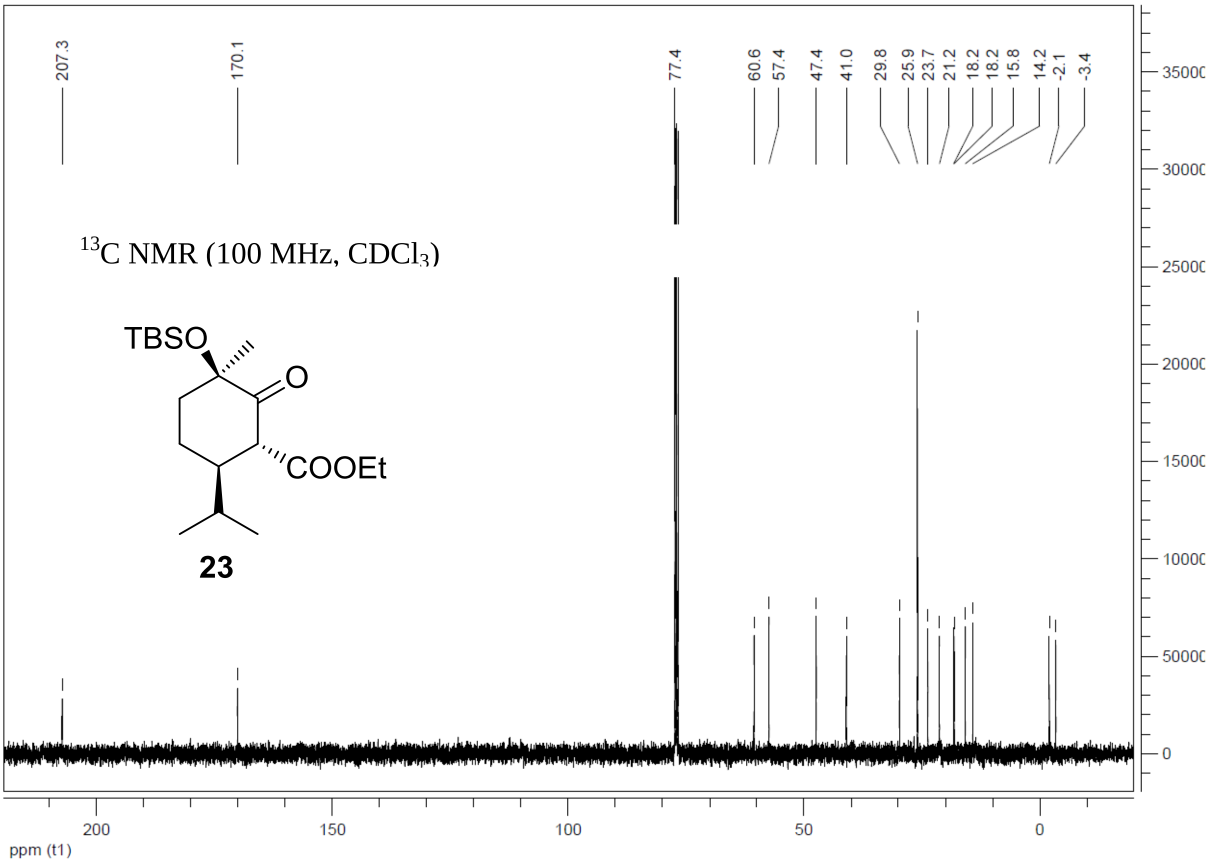
207.3
170.1
77.4
60.6
57.4
47.4
41.0
29.8
25.9
23.7
21.2
18.2
18.2
15.8
14.2
-2.1
-3.4
13C NMR (100 MHz, CDCl3)
TBSO
O
COOEt
23
ppm (t1)

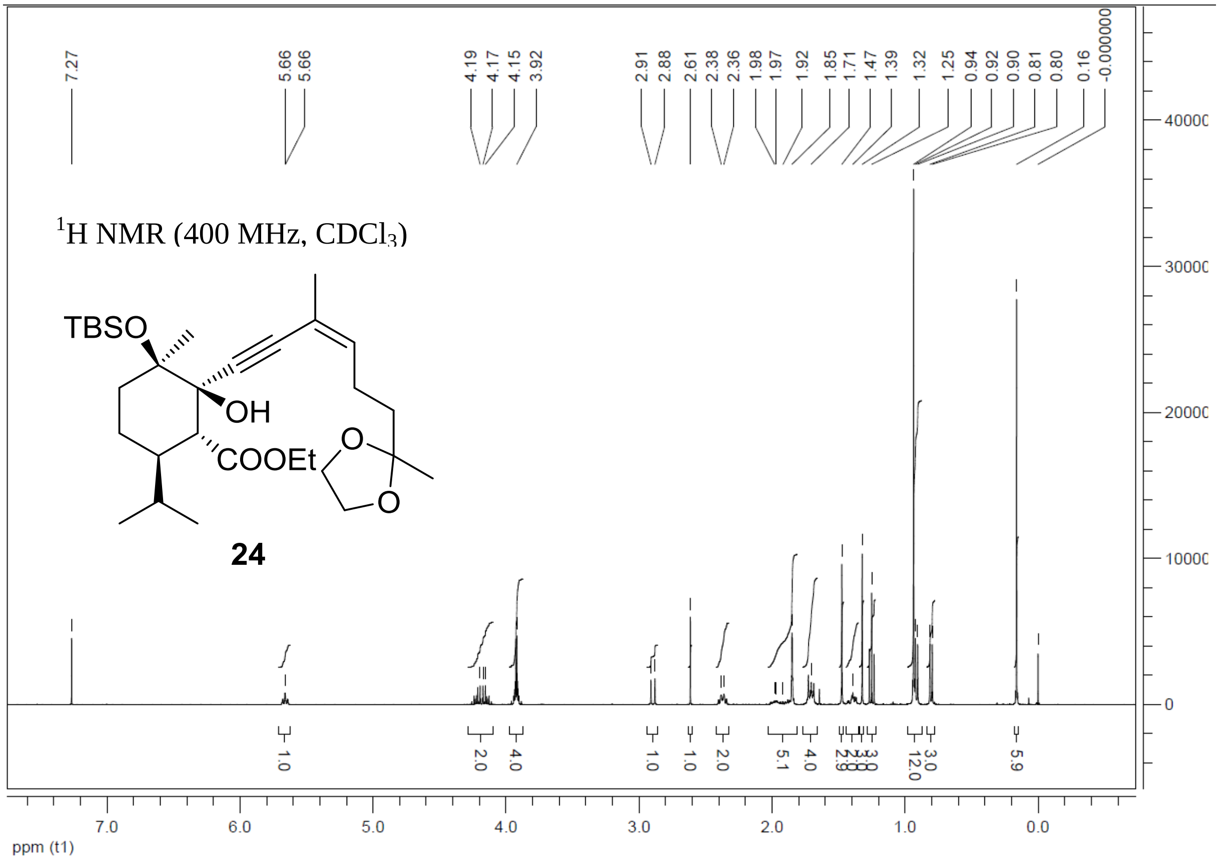

7.27
5.66
5.66
4.19
4.17
4.15
3.92
2.91
2.88
2.61
2.38
2.36
1.98
1.97
1.92
1.85
1.71
1.47
1.39
1.32
1.25
0.94
0.92
0.90
0.81
0.80
0.16
-0.000000
1H NMR (400 MHz, CDCl3)
TBSO
OH
COOEt
O
O
24
1.0
2.0
4.0
1.0
1.0
2.0
5.1
4.0
12.0
3.0
5.9
7.0
6.0
5.0
4.0
3.0
2.0
1.0
0.0
ppm (t1)

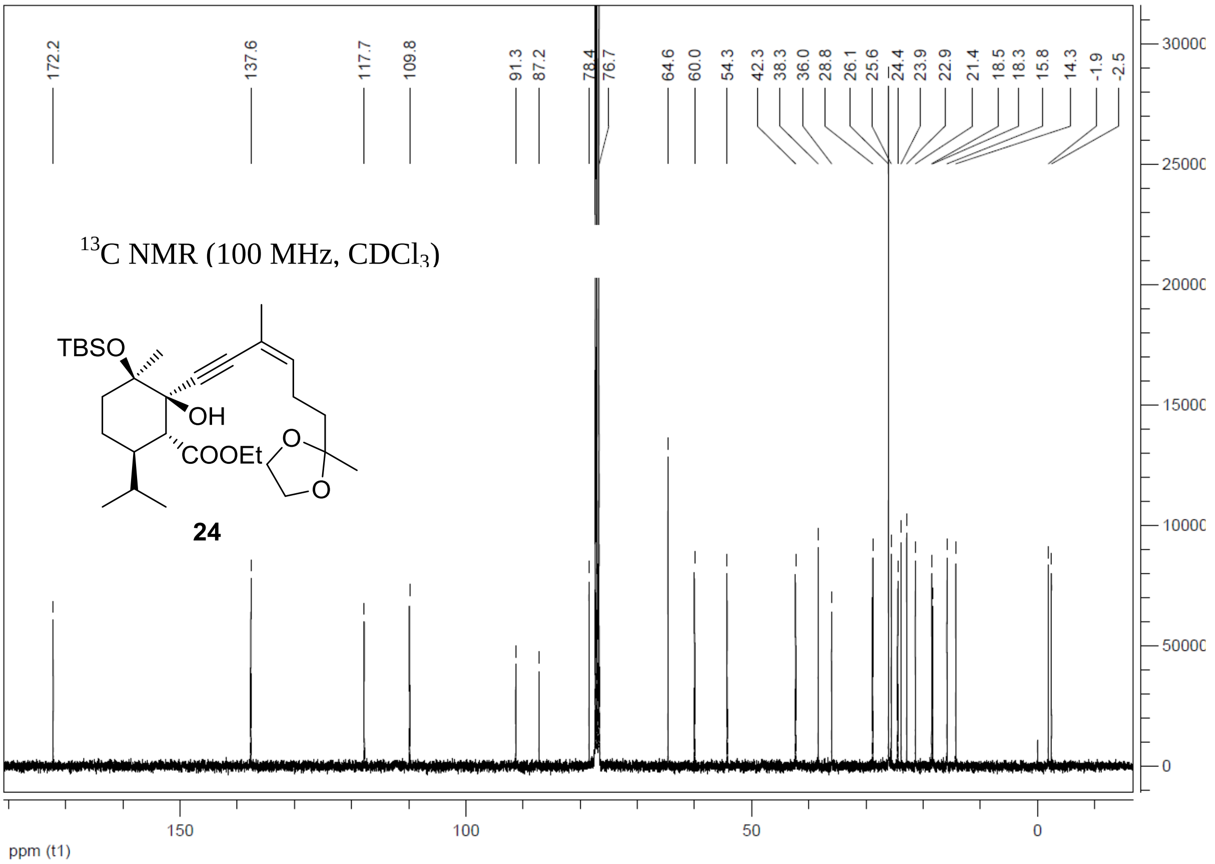

172.2
137.6
117.7
109.8
91.3
87.2
78.4
76.7
64.6
60.0
54.3
42.3
38.3
36.0
28.8
26.1
25.6
24.4
23.9
22.9
21.4
18.5
18.3
15.8
14.3
-1.9
-2.5
13C NMR (100 MHz, CDCl3)
TBSO
OH
COOEt
O
O
24
150
100
50
0
ppm (t1)

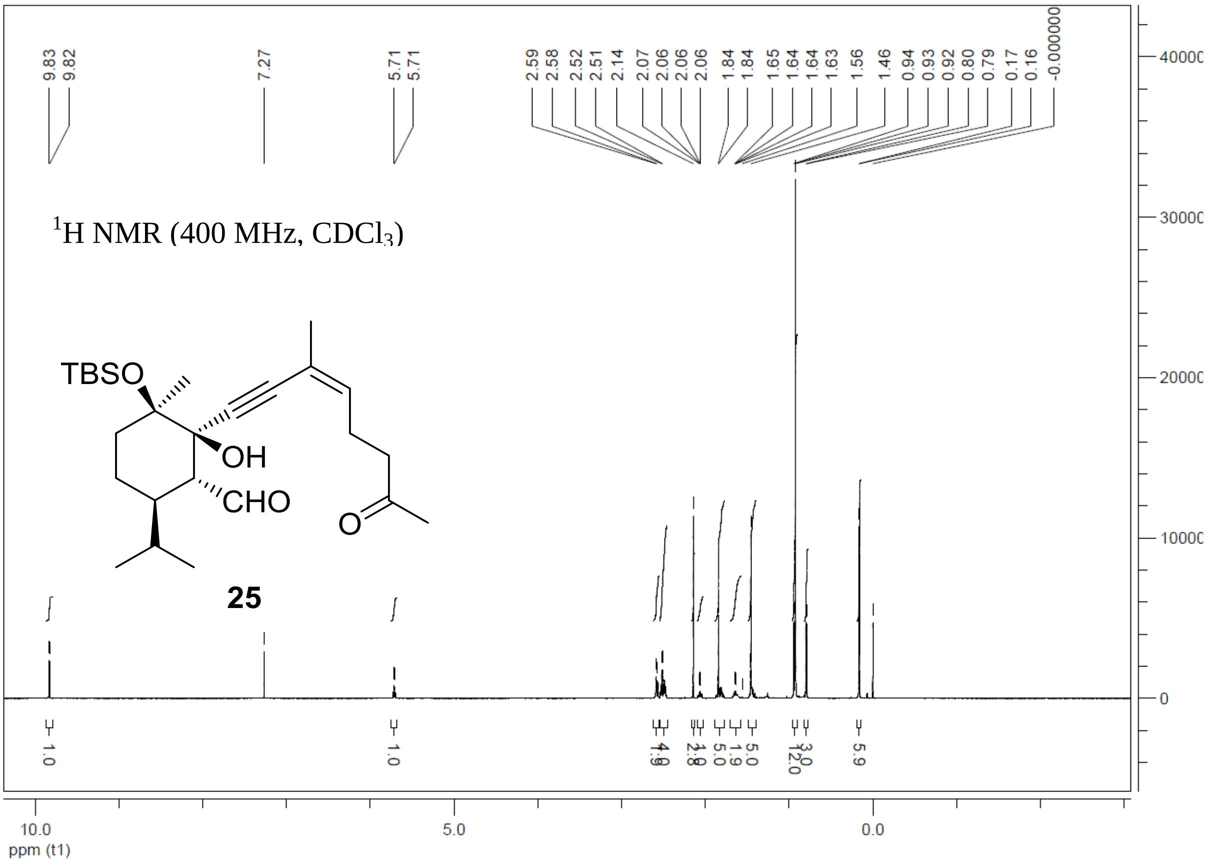
9.83
9.82
7.27
5.71
5.71
2.59
2.58
2.52
2.51
2.14
2.07
2.06
2.06
2.06
1.84
1.84
1.65
1.64
1.64
1.63
1.56
1.46
0.94
0.93
0.92
0.80
0.79
0.17
0.16
-0.000000
1H NMR (400 MHz, CDCl3)
TBSO
OH
CHO
O
25
1.0
1.0
5.0
1.9
5.0
12.0
3.0
5.9
10.0
5.0
0.0
ppm (t1)
40000
30000
20000
10000
0

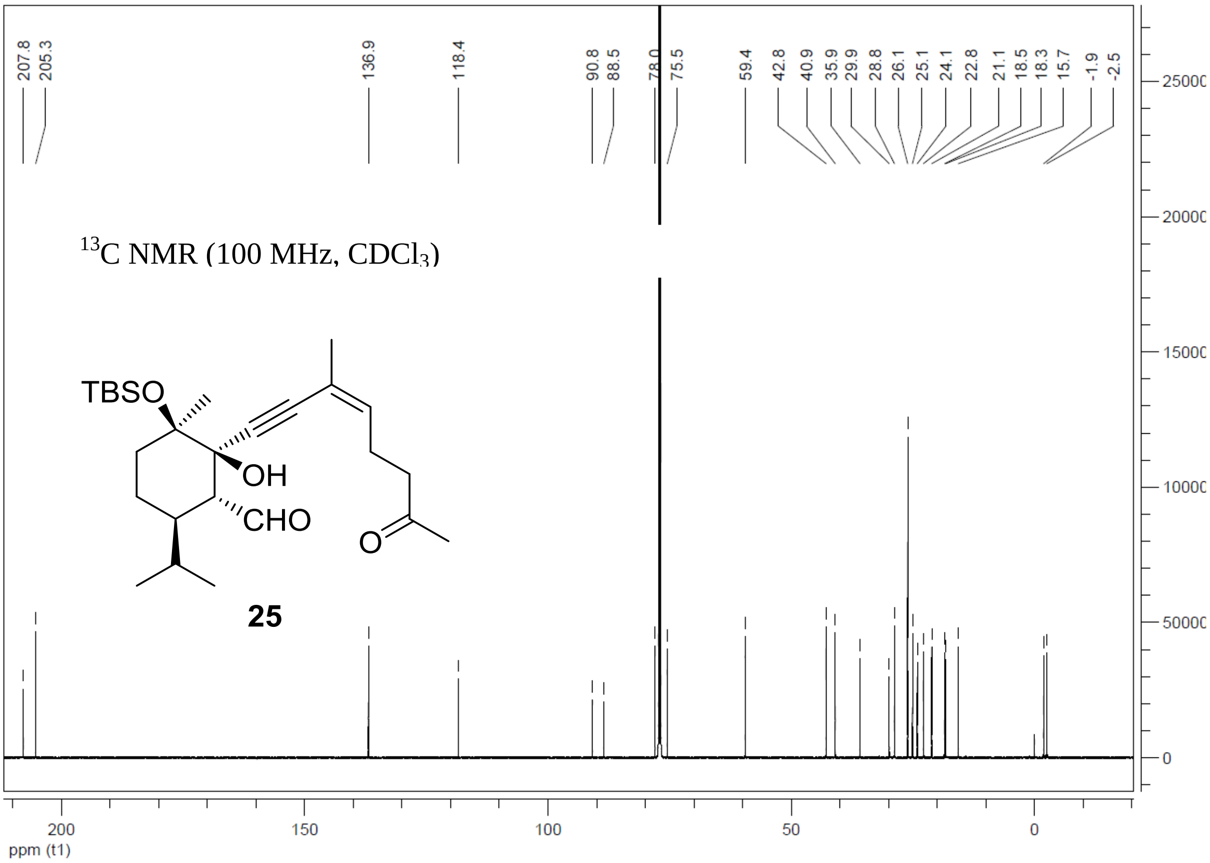
207.8
205.3
136.9
118.4
90.8
88.5
78.0
75.5
59.4
42.8
40.9
35.9
29.9
28.8
26.1
25.1
24.1
22.8
21.1
18.5
18.3
15.7
-1.9
-2.5
13C NMR (100 MHz, CDCl3)
TBSO
OH
CHO
O
25
200
150
100
50
0
ppm (t1)
25000
20000
15000
10000
50000
0

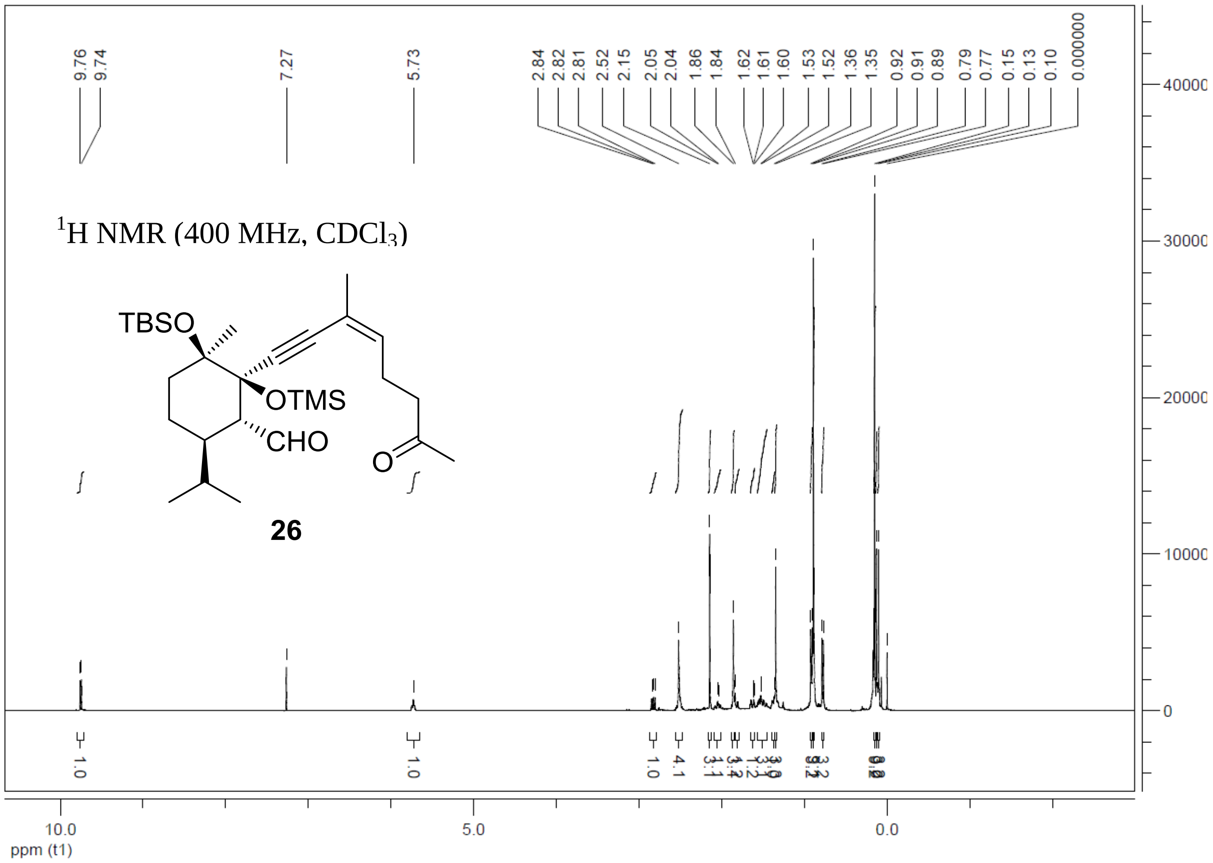

1H NMR (400 MHz, CDCl3)
TBSO
OTMS
CHO
O
26
ppm (t1)

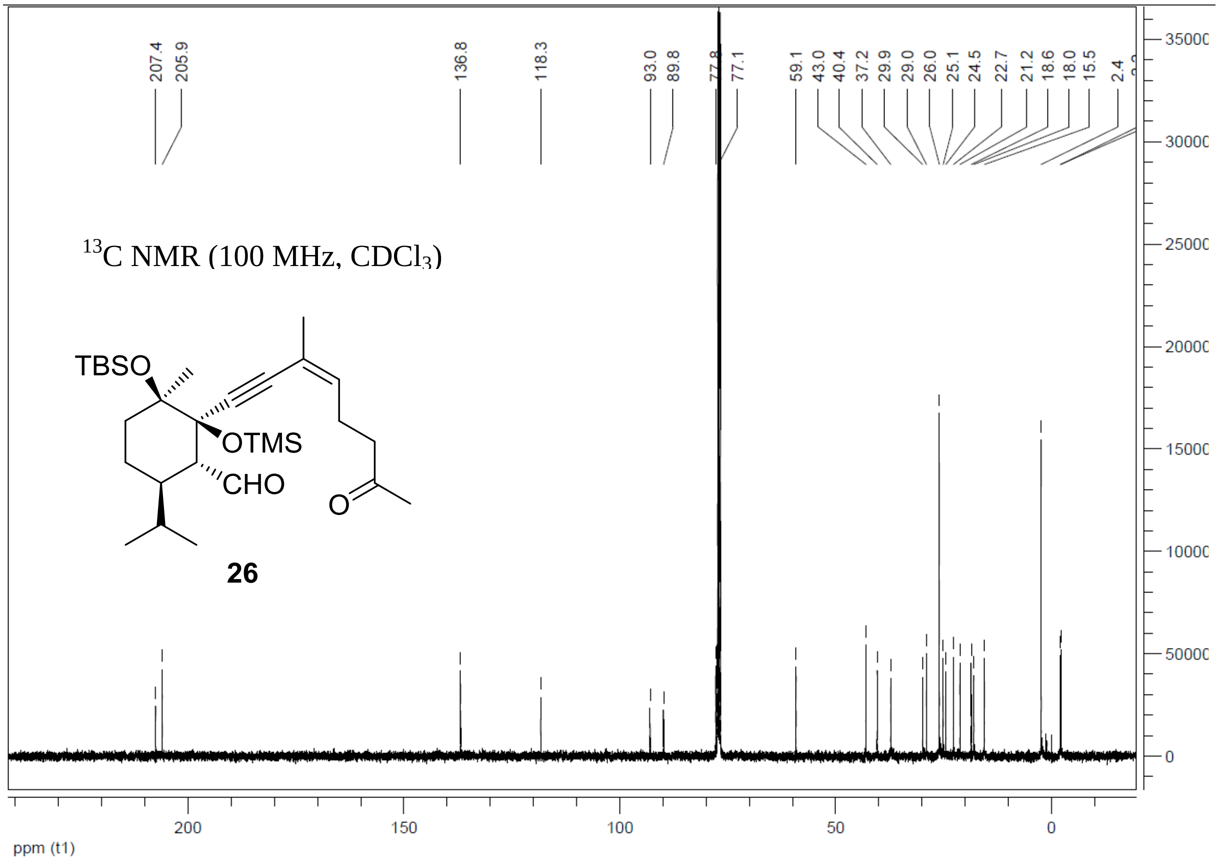

13C NMR (100 MHz, CDCl3)
TBSO
OTMS
CHO
O
26
ppm (t1)

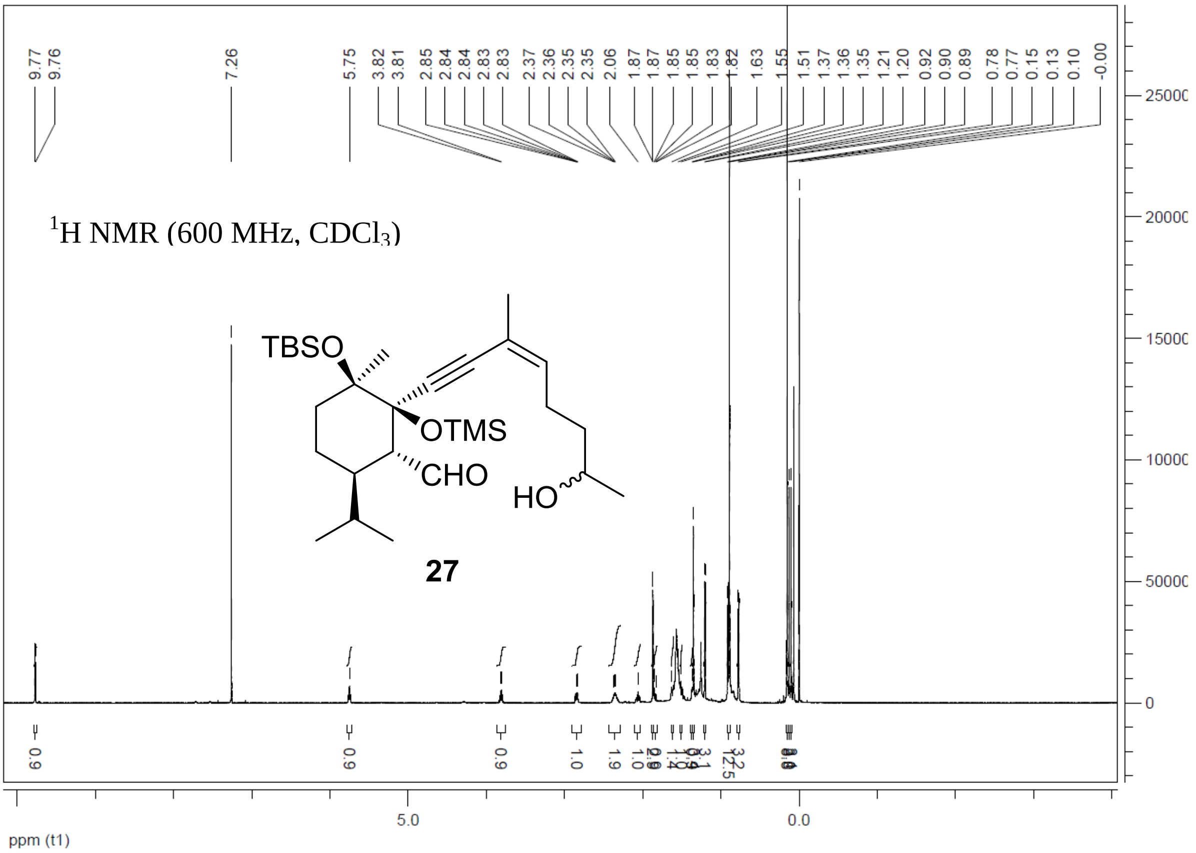
¹H NMR (600 MHz, CDCl₃)
TBSO
OTMS
CHO
HO
27
ppm (t1)

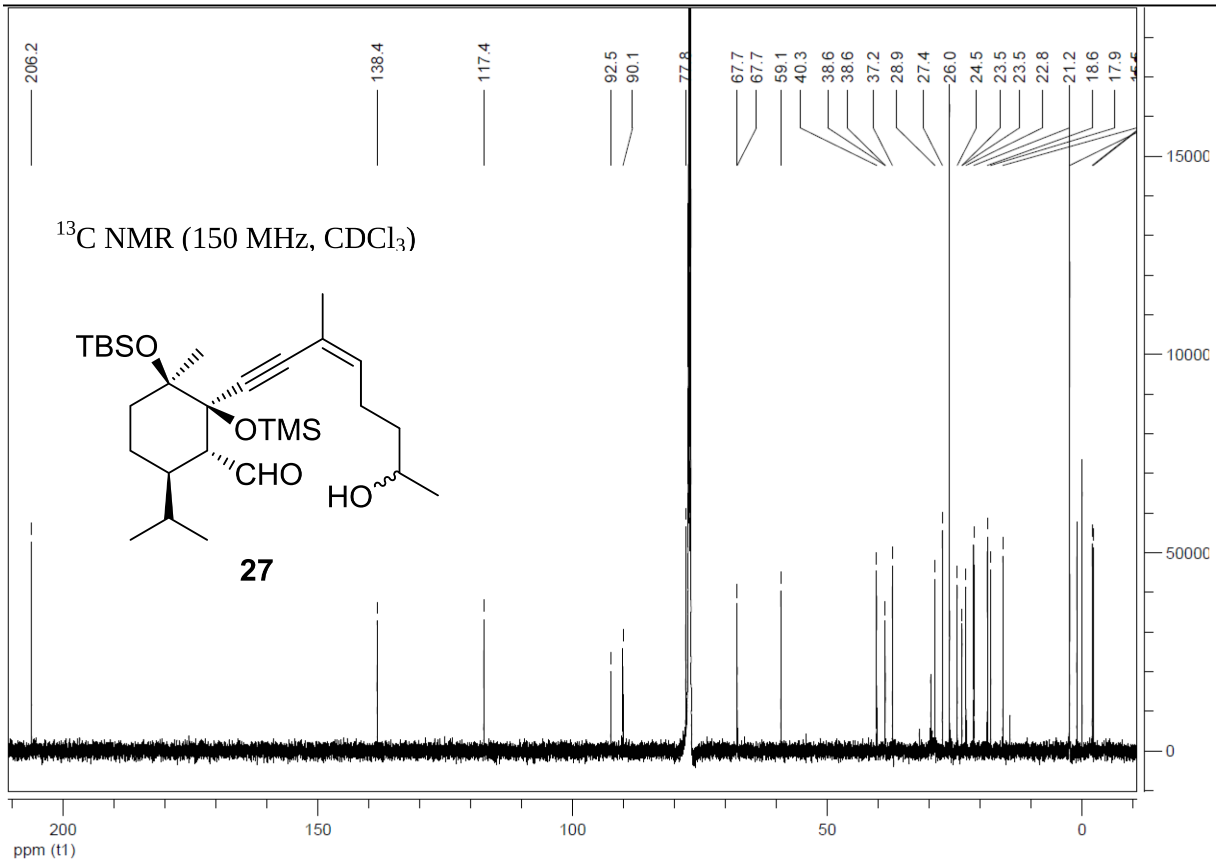
¹³C NMR (150 MHz, CDCl₃)
TBSO
OTMS
CHO
HO
27
ppm (t1)

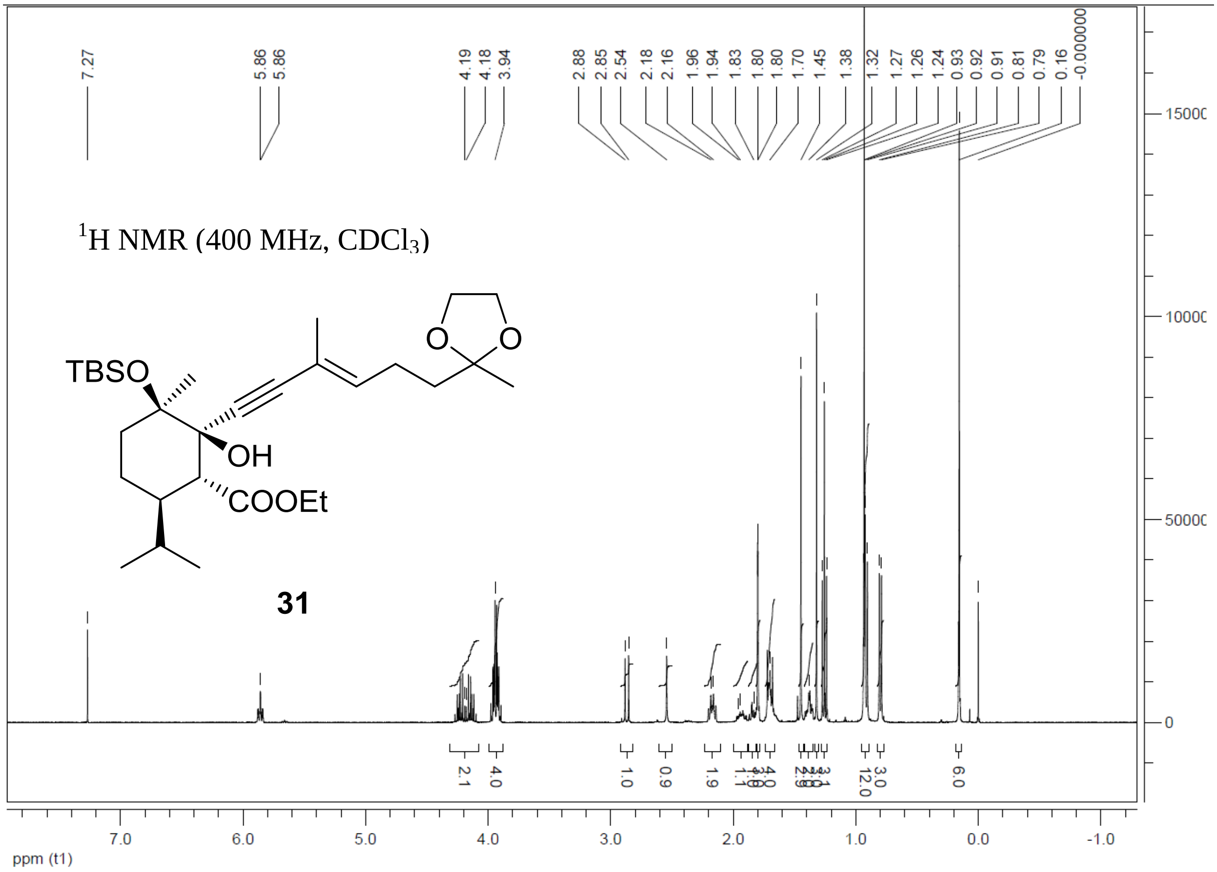

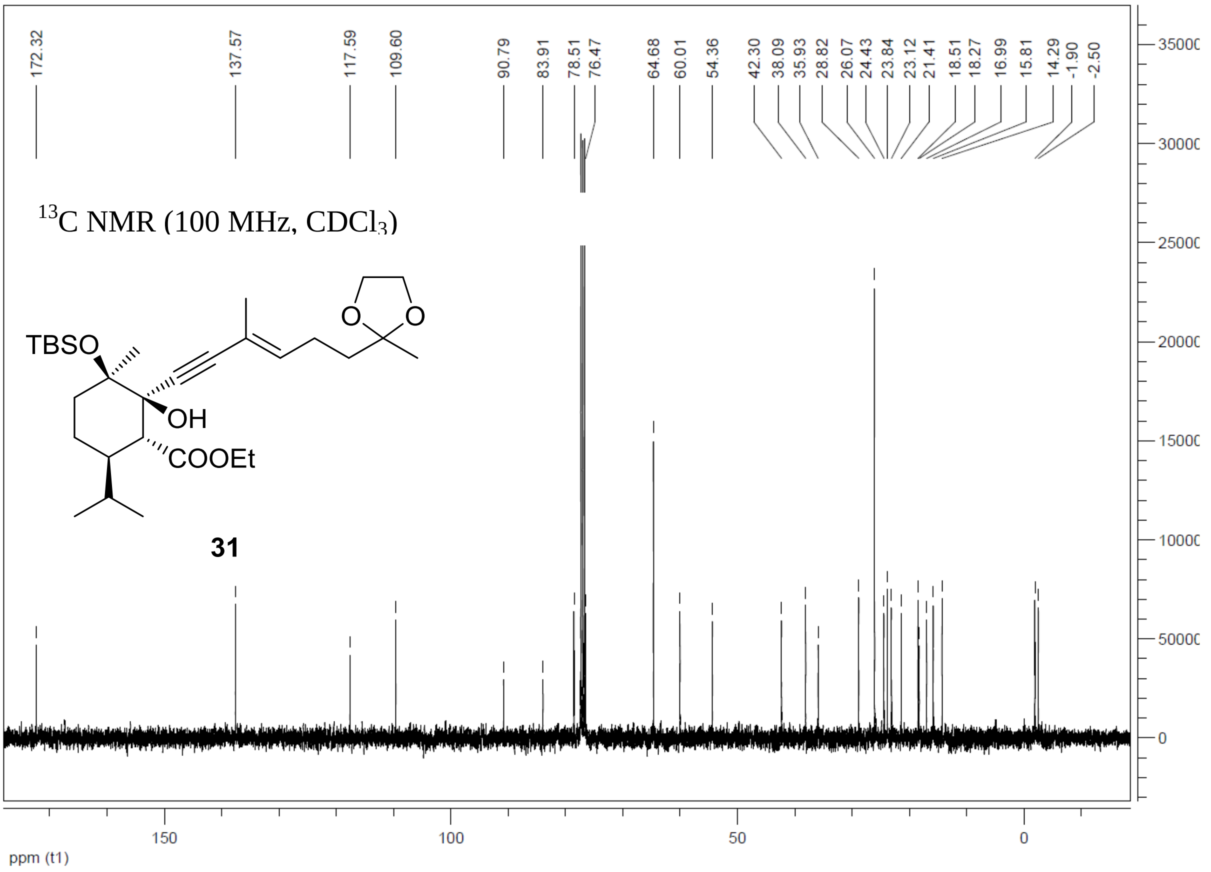

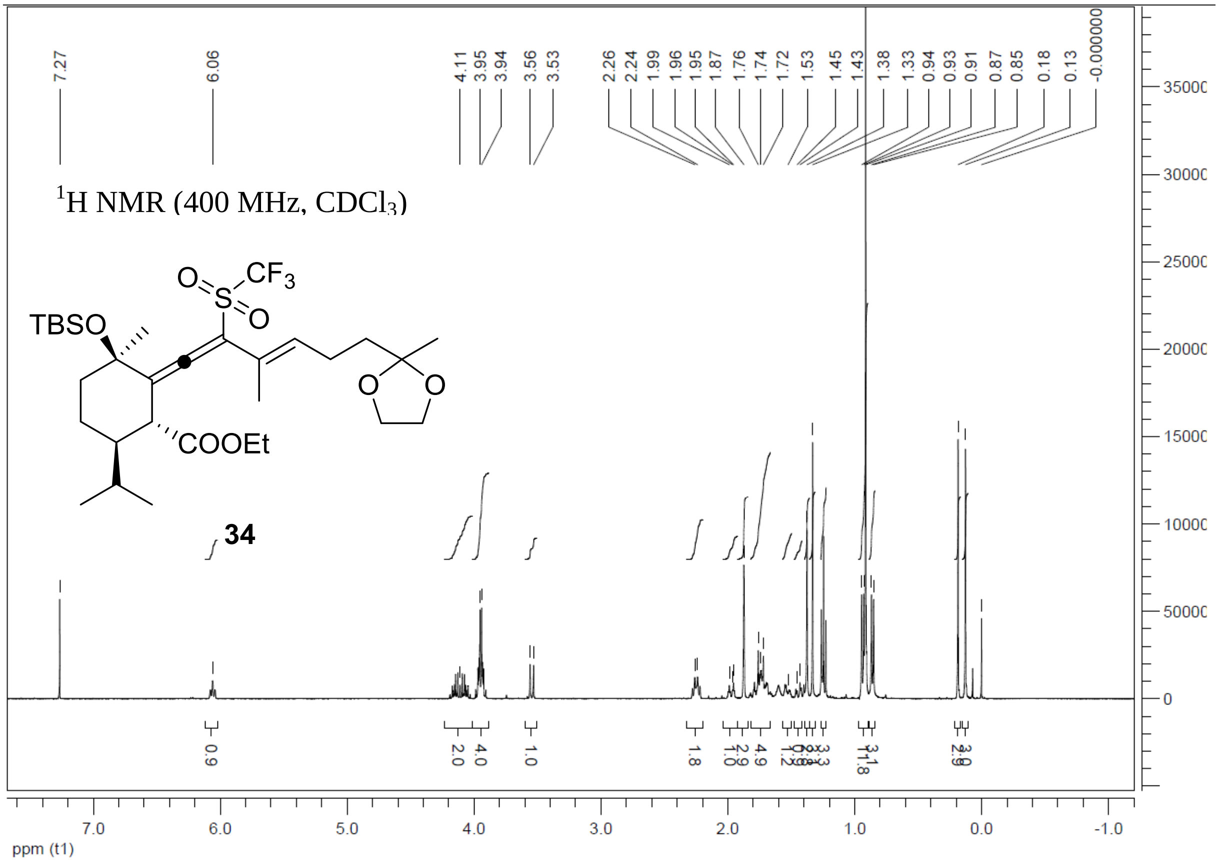

1H NMR (400 MHz, CDCl3)
TBSO
CF3
COOEt
34
ppm (t1)

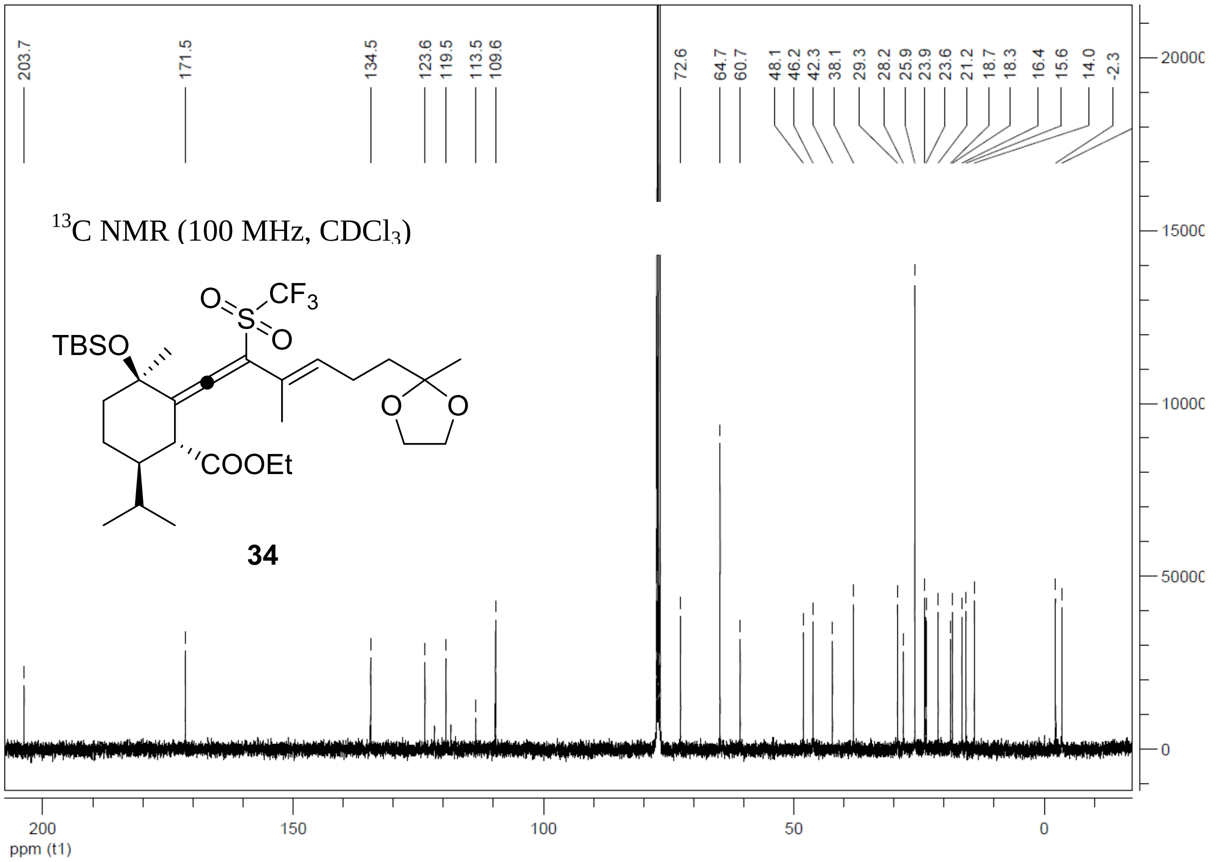

13C NMR (100 MHz, CDCl3)
TBSO
CF3
COOEt
34
ppm (t1)

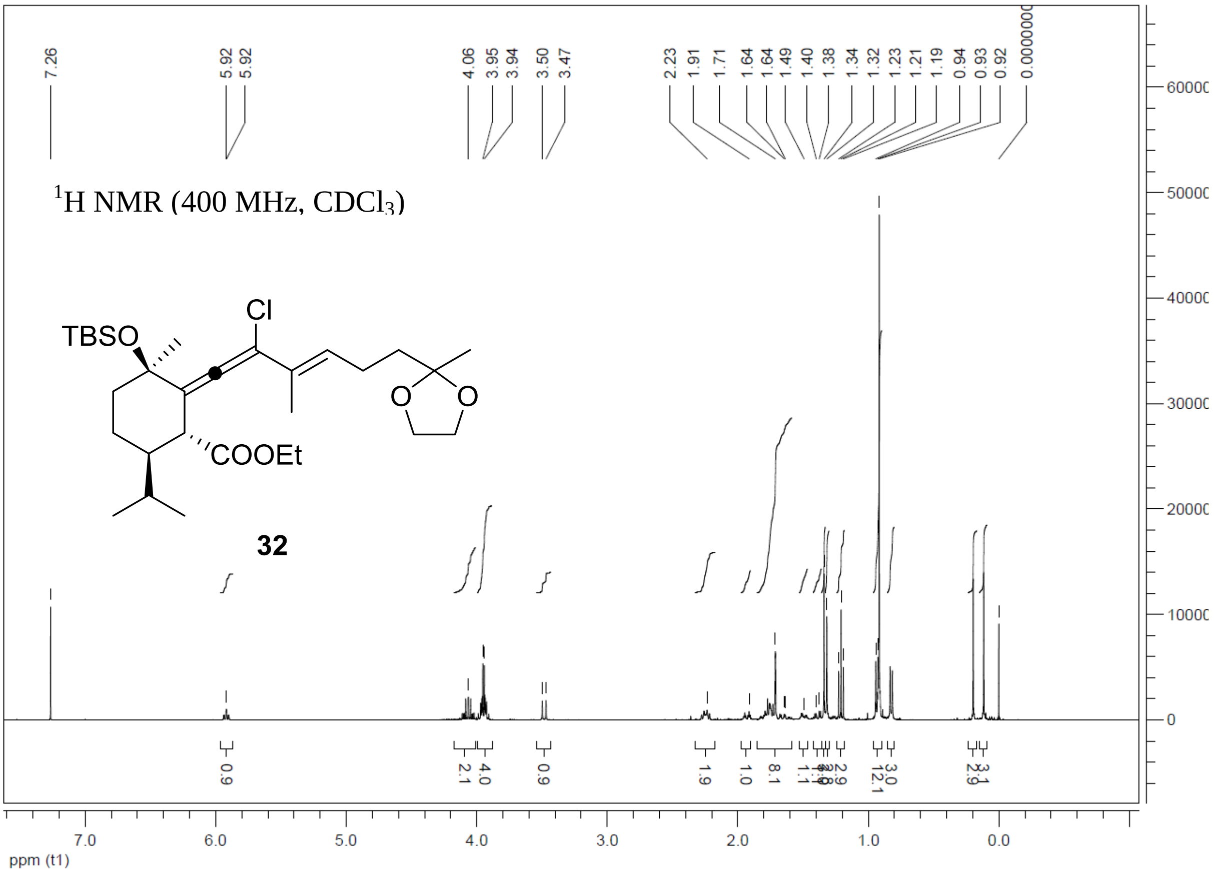
1H NMR (400 MHz, CDCl3)
TBSO
Cl
COOEt
O
O
32
7.26
5.92
5.92
4.06
3.95
3.94
3.50
3.47
2.23
1.91
1.71
1.64
1.64
1.49
1.40
1.38
1.34
1.32
1.23
1.21
1.19
0.94
0.93
0.92
0.0000000
ppm (t1)

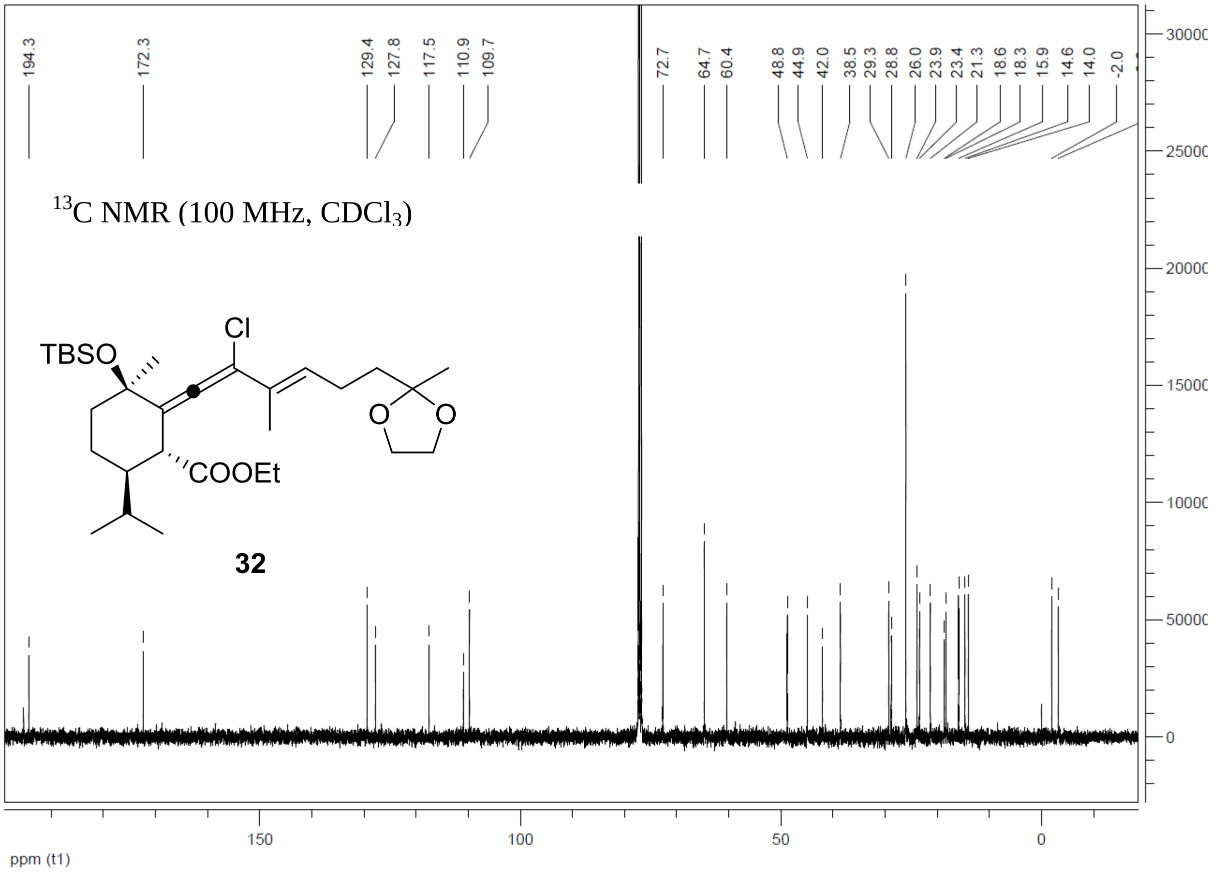
13C NMR (100 MHz, CDCl3)
TBSO
Cl
COOEt
O
O
32
194.3
172.3
129.4
127.8
117.5
110.9
109.7
72.7
64.7
60.4
48.8
44.9
42.0
38.5
29.3
28.8
26.0
23.9
23.4
21.3
18.6
18.3
15.9
14.6
14.0
-2.0
ppm (t1)

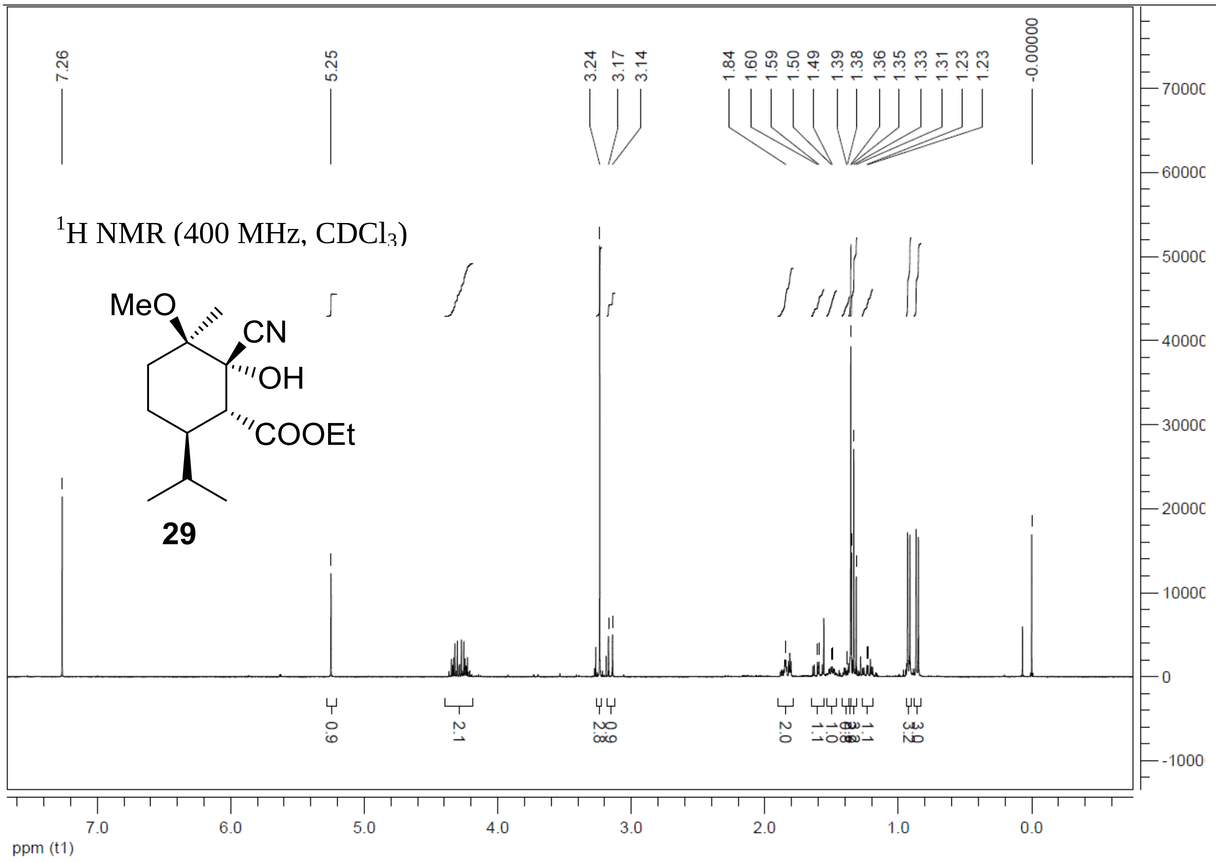

^{1}H NMR (400 MHz, CDCl$_3$)
MeO
CN
OH
COOEt
29
ppm (t1)

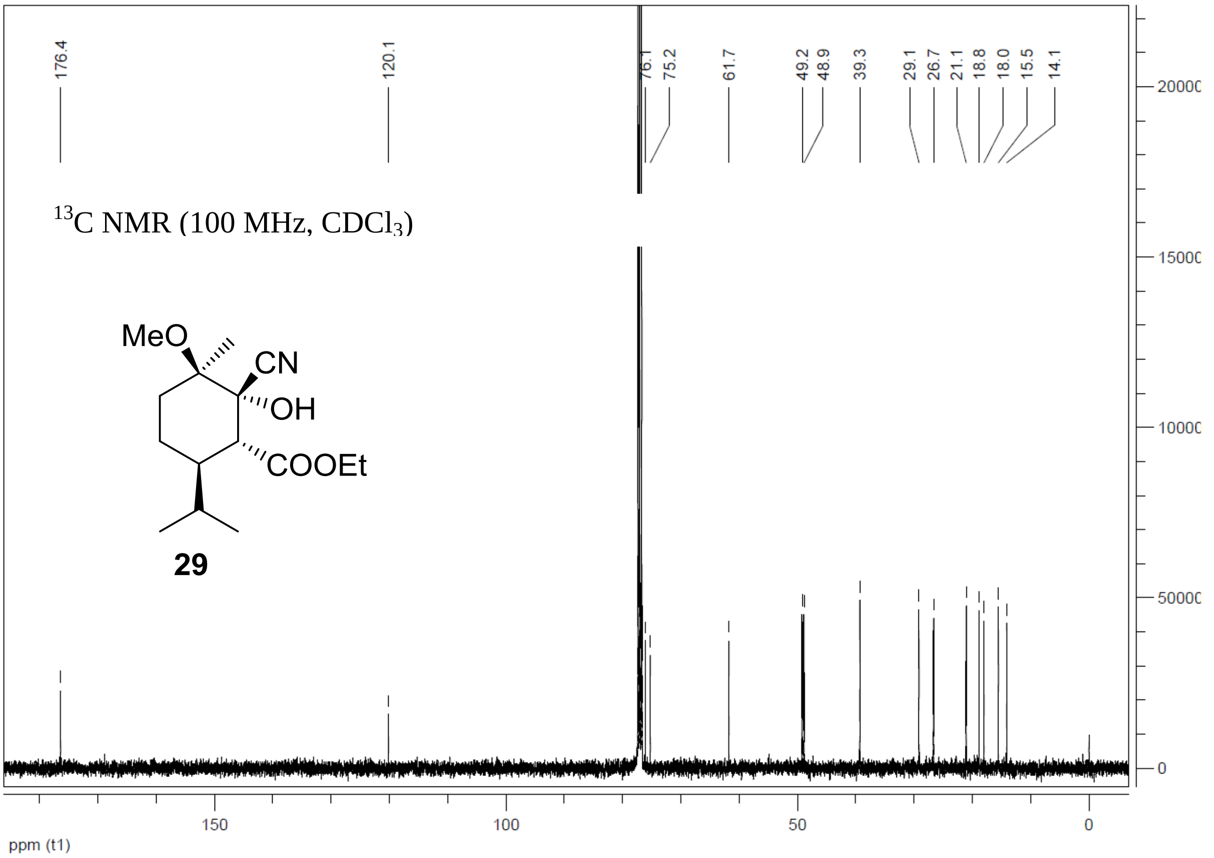

^{13}C NMR (100 MHz, CDCl$_3$)
MeO
CN
OH
COOEt
29
ppm (t1)

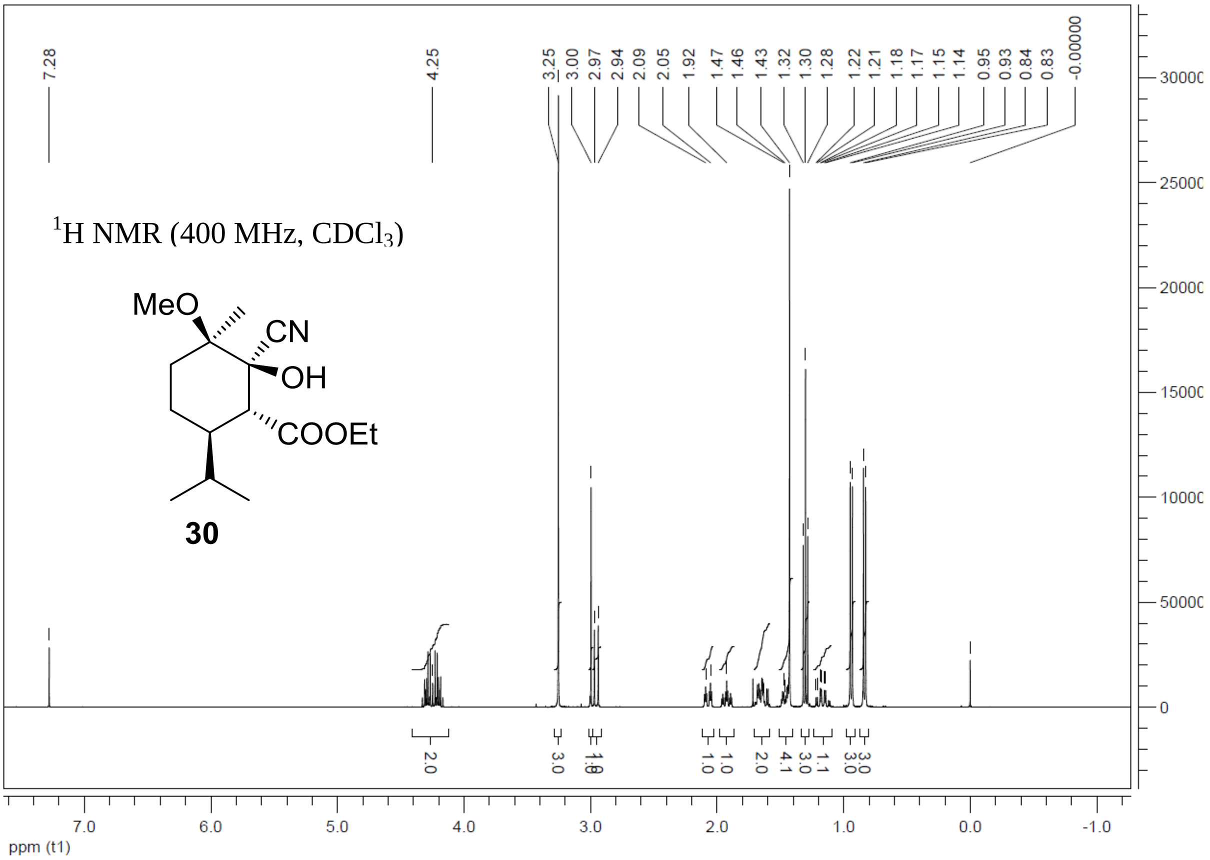

1H NMR (400 MHz, CDCl3)
MeO
CN
OH
COOEt
30
7.28
4.25
3.25 3.00 2.97 2.94 2.09 2.05 1.92 1.47 1.46 1.43 1.32 1.30 1.28 1.22 1.21 1.18 1.17 1.15 1.14 0.95 0.93 0.84 0.83 -0.00000
2.0 3.0 1.0 1.0 1.0 1.0 2.0 4.1 3.0 1.1 3.0 3.0
ppm (t1)

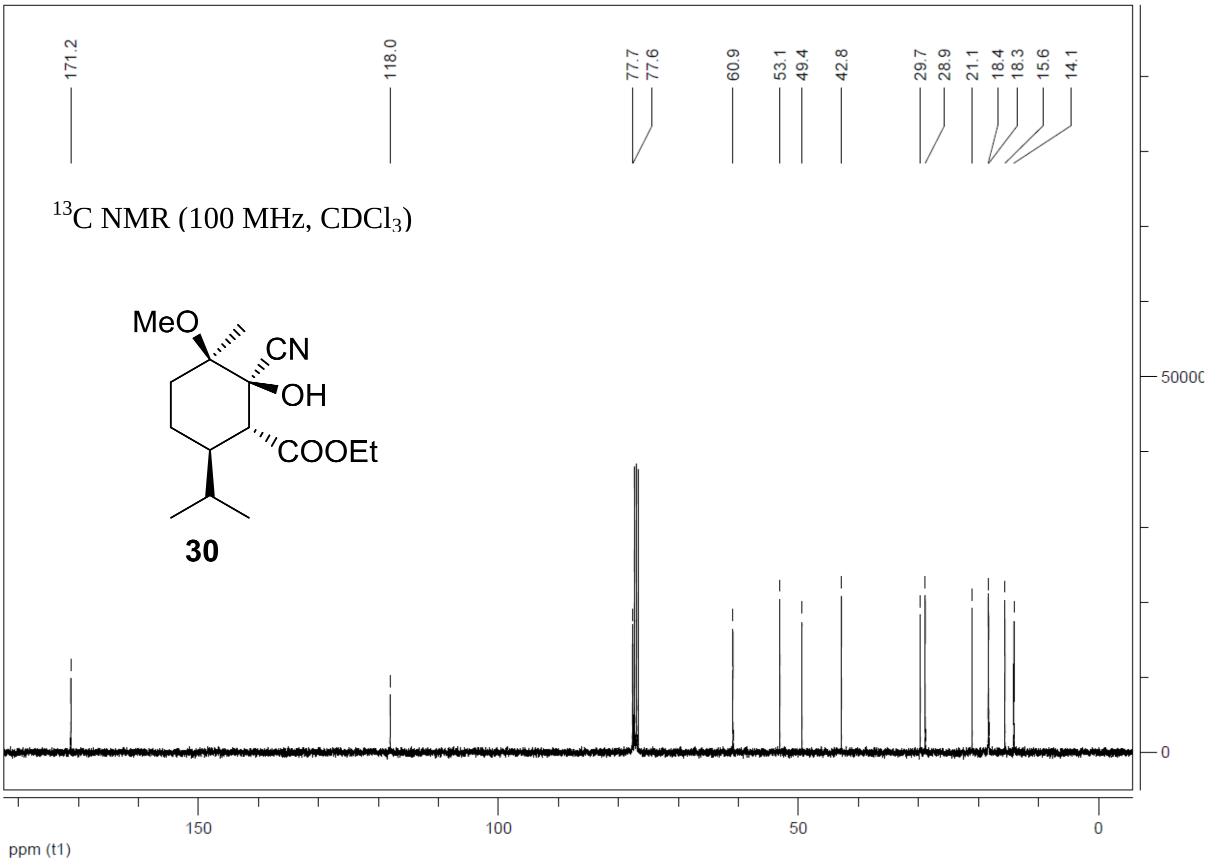

13C NMR (100 MHz, CDCl3)
MeO
CN
OH
COOEt
30
171.2
118.0
77.7 77.6
60.9
53.1 49.4
42.8
29.7 28.9 21.1 18.4 18.3 15.6 14.1
ppm (t1)
